# Intramolecular C–H Oxidation in Iron(V)-oxo-carboxylato Species Relevant in the γ-Lactonization of Alkyl Carboxylic Acids

**DOI:** 10.1021/acscatal.4c01258

**Published:** 2024-09-11

**Authors:** Andrea Álvarez-Núñez, Rudraditya Sarkar, Valeria Dantignana, Jin Xiong, Yisong Guo, Josep M. Luis, Miquel Costas, Anna Company

**Affiliations:** † Institut de Química Computacional i Catàlisi (IQCC), Departament de Química, Universitat de Girona, C/Ma Aurèlia Capmany 69, 17003 Girona, Catalonia, Spain; ‡ Chemistry Department, Carnegie Mellon University, Pittsburgh, Pennsylvania 15213, United States; § Department of Chemistry, School of Science, Gandhi Institute of Technology and Management (GITAM), Hyderabad502329, India

**Keywords:** high-valent iron oxo, γ-lactones, oxidation reactions, bioinspired chemistry, nonheme iron enzymes

## Abstract

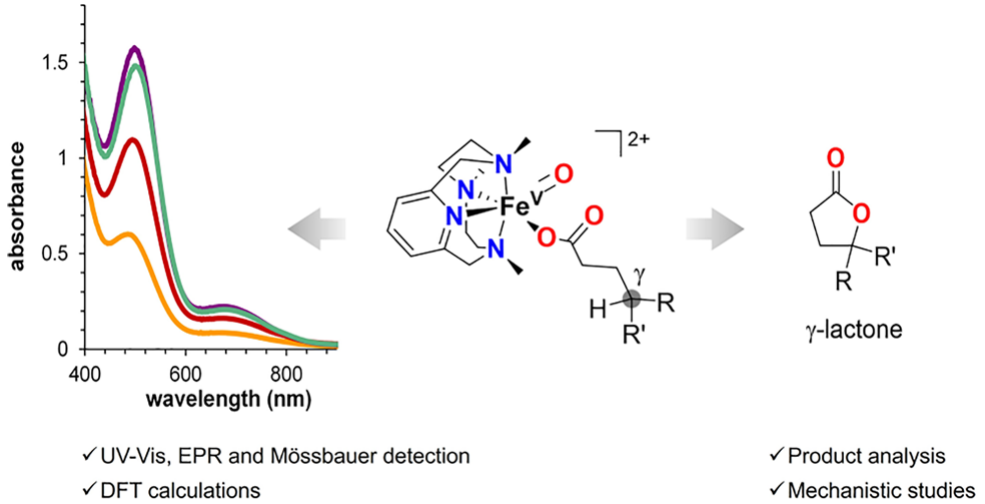

High-valent oxoiron species have been invoked as oxidizing agents in a variety of iron-dependent oxygenases. Taking inspiration from nature, selected nonheme iron complexes have been developed as catalysts to elicit C–H oxidation through the mediation of putative oxoiron(V) species, akin to those proposed for Rieske oxygenases. The addition of carboxylic acids in these iron-catalyzed C–H oxidations has proved highly beneficial in terms of product yields and selectivities, suggesting the direct involvement of iron(V)-oxo-carboxylato species. When the carboxylic acid functionality is present in the alkane substrate, it acts as a directing group, enabling the selective intramolecular γ-C–H hydroxylation that eventually affords γ-lactones. While this mechanistic frame is solidly supported by previous mechanistic studies, direct spectroscopic detection of the key iron(V)-oxo-carboxylato intermediate and its competence for engaging in the selective γ–C–H oxidation leading to lactonization have not been accomplished. In this work, we generate a series of well-defined iron(V)-oxo-carboxylato species (**2c**–**2f**) differing in the nature of the bound carboxylate ligand. Species **2c**–**2f** are characterized by a set of spectroscopic techniques, including UV–vis spectroscopy, cold-spray ionization mass spectrometry (CSI-MS), and, in selected cases, EPR and Mössbauer spectroscopies. We demonstrate that **2c**–**2f** undergo site-selective γ-lactonization of the carboxylate ligand in a stereoretentive manner, thus unequivocally identifying metal-oxo-carboxylato species as the powerful yet selective C–H cleaving species in catalytic γ-lactonization reactions of carboxylic acids. Reactivity experiments confirm that the intramolecular formation of γ-lactones is in competition with the intermolecular oxidation of external alkanes and olefins. Finally, mechanistic studies, together with DFT calculations, support a mechanism involving a site-selective C–H cleavage in the γ-position of the carboxylate ligand by the oxo moiety, followed by a fast carboxylate rebound, eventually leading to the selective formation of γ-lactones.

## Introduction

Taking inspiration from nature, selected nonheme iron complexes have been developed as catalysts to elicit site-selective and stereorententive C–H oxidation upon reaction with H_2_O_2_.^1^ Selectivity and stereospecificity exhibited by these catalysts provide strong support in favor of the intermediacy of metal-based oxidants akin to those proposed in Rieske oxygenases, namely, oxoiron(V) species.^2,3^ Pioneering works in this field were carried out by Que and co-workers, who reported stereospecific C–H hydroxylation reactions using an iron(II) complex containing the tris(2-pyridylmethyl)amine (tpa) ligand,^4^ a nitrogen-based tetradentate architecture. Several other ligand architectures have successfully led to efficient iron catalysts. In some instances, synthetically useful alkane hydroxylation reactions with predictable selectivity in complex molecules containing multiple C–H bonds have been achieved, while enantioselective transformations have been accomplished with the use of the appropriate chiral ligands.^5−7^ The structural diversity of these ligands is quite large, but a quite common feature is the presence of tetradentate nitrogen-based architectures that wrap around the metal center, leaving two labile positions in a relative *cis*-configuration for interaction with the oxidant, a key feature also observed in Rieske oxygenases.^8^

**Scheme 1:**
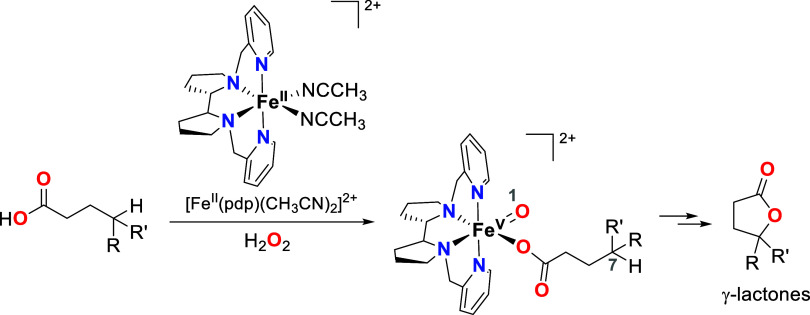
Formation of Iron(V)-oxo-carboxylato Species upon Reaction of [Fe^II^(pdp)(CH_3_CN)_2_]^2+^ with H_2_O_2_ and a Carboxylic Acid Substrate, along with the Generation of the Corresponding γ-Lactone after Intramolecular Oxidation of the Tertiary C–H Bond in the γ-Position of the Carboxylato Ligand: the Reaction Most Likely Occurs through a 1,7-HAT Mechanism^12^

The addition of carboxylic acids in these iron-catalyzed C–H oxidation reactions has proven highly beneficial for ensuring optimum product yields and chemoselectivities. Several mechanistic studies converge that carboxylic acid binds the metal center and assists the cleavage of H_2_O_2_, affording a high-valent iron(V)-oxo-carboxylato species directly responsible for substrate oxidation.^6,9^ This was first proposed by Mas-Ballesté and Que, who reported the *cis*-1,2-hydroxyacetoxylation of olefins as minor side products in the catalytic epoxidation of olefins by H_2_O_2_ in the presence of acetic acid catalyzed by [Fe^II^(tpa)(CH_3_CN)_2_]^2+^, supporting the involvement of [Fe^V^(O)(OAc)(tpa)]^2+^ as the active species.^10,11^ Interestingly, a carboxylic acid functionality present in the alkane substrate can act as a directing group, enabling the selective intramolecular γ-C–H hydroxylation, which eventually affords valuable γ-lactones. This reaction was first reported by White and co-workers using [Fe^II^(pdp)(CH_3_CN)_2_]^2+^ as catalyst, enabling the oxidation of secondary and tertiary C–H bonds (Scheme 1).^12^ The precise site selectivity and predictability of these carboxylate-directed reactions have found application in complex organic synthesis.^13−18^ Furthermore, when iron is replaced by manganese, not only secondary and tertiary C–H bonds can be activated, but also the strong primary C–H bonds of methyl groups placed in the γ position of the carboxylic acid alkane substrate can be oxidized.^19−22^ In such cases, the use of chiral ligands leads to enantio- and diastereoselective lactonization of unactivated secondary and primary C–H bonds. In any case, iron(V)-oxo-carboxylato and manganese(V)-oxo-carboxylato have been postulated as the key oxidizing species in these lactonization reactions. These species are proposed to engage in an unusual 1,7-hydrogen abstraction reaction by the oxo ligand, differing from the most commonly found intramolecular 1,5 and 1,6-HAT reactions.^23,24^ However, direct detection of the γ-C–H cleaving intermediate has not yet been accomplished.

**Scheme 2:**
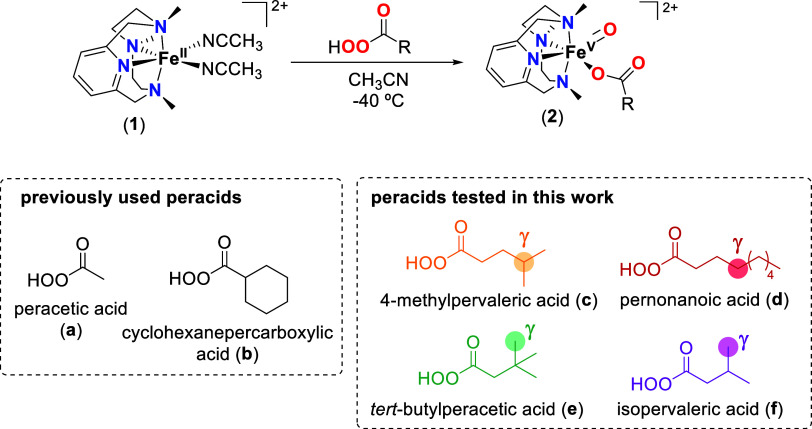
Top: Generation of Iron(V)-oxo-carboxylato Species (**2**) by Reaction of [Fe^II^(PyNMe_3_)(NCCH_3_)_2_]^2+^ (**1**) with Peracids at Low Temperatures in Acetonitrile; Bottom: Structure of the Peracids Previously Used for the Preparation of **2** and the Ones Tested in This Work

Direct spectroscopic evidence of the oxoiron(V) species^25,26^ proposed to be responsible for catalytic oxidation reactions with bioinspired nonheme catalysts is scarce and until quite recently limited to EPR studies of samples where *S* = 1/2 species assigned to Fe(V) intermediates accumulate up to 1–2%.^27−29^ Theoretical studies also suggested the viability of such oxidants.^30^ Larger accumulations enabling full spectroscopic characterization have so far been only accomplished for [Fe^V^(O)(carboxylate)(PyNMe_3_)]^2+^ (**2**, Scheme 2), where the enhanced stability of the ferryl unit may be tentatively attributed to the sterically demanding and oxidatively robust PyNMe_3_ ligand.^31^ This species was synthesized by a reaction of the iron(II) precursor [Fe^II^(PyNMe_3_)(NCCH_3_)_2_]^2+^ (**1**) with peracetic acid (**a**) or cyclohexanepercarboxylic acid (**b**) in acetonitrile at −40 °C (Scheme 2). According to detailed kinetic studies,^31^ generation of **2** occurs through a two-step process involving the one-electron oxidation of the Fe^II^ center to Fe^III^ with 0.5 equiv of the peracid, followed by its two-electron oxidation with another equivalent of peracid affording the oxoiron(V) compound. Species **2** is highly reactive, and it decomposes over minutes, even at this low temperature. In spite of its metastable character, the compound could be spectroscopically (EPR, Resonance Raman, Mössbauer and XAS spectroscopies) and theoretically characterized as an iron(V)-oxo-carboxylato species (**2**).^32^ An alternative description as an iron(IV)-oxocarboxylato species, bearing a single-electron bond between the oxo and the carboxylate ligands, has also been proposed.^33^ Stopped-flow kinetic analysis carried out with the iron(V)-oxo-acetato species **2a** (generated by the reaction of **1** with **a**) permitted us to determine second-order rate constants for hydrogen atom transfer (HAT)^31^ and oxygen atom transfer (OAT) reactions.^34^ Besides demonstrating that **2a** is the kinetically competent oxidant, kinetic analyses provided reaction rates that indicate that **2a** is the most reactive oxoiron compound described so far in these two types of reactions. Furthermore, in contrast to oxoiron(IV) complexes, which engage in HAT reactions generating long-lived carbon-centered radicals,^35,36^**2a** performs stereospecific and site-selective C–H hydroxylation through a HAT mechanism.^31^ Moreover, the system is catalytically competent, so that **2** can be considered as a model for the widely postulated active species in iron-catalyzed C–H hydroxylation and C=C epoxidation reactions.

Considering that iron(V)-oxo-carboxylato species have been proposed to be the C–H cleaving species in intramolecular γ-lactonization reactions, access to **2** provides an opportunity for interrogating this mechanistic scenario. Accordingly, in the present work, we study the ability of compound **2** to perform intramolecular oxidation of its bound carboxylato ligand by using different alkyl peracids for its generation (Scheme 2). Results disclosed herein show that, indeed, compound **2** is kinetically competent to carry out an exquisite site-selective γ-lactonization of the bound carboxylate ligand in a stereoretentive manner, thus identifying metal-oxo-carboxylato species as the powerful yet selective C–H cleaving species in catalytic γ-lactonization reactions of carboxylic acids.

## Results and Discussion

### Formation and Decay of the Iron(V)-oxo-carboxylato Species **2c**–**2f**

**Figure 1:**
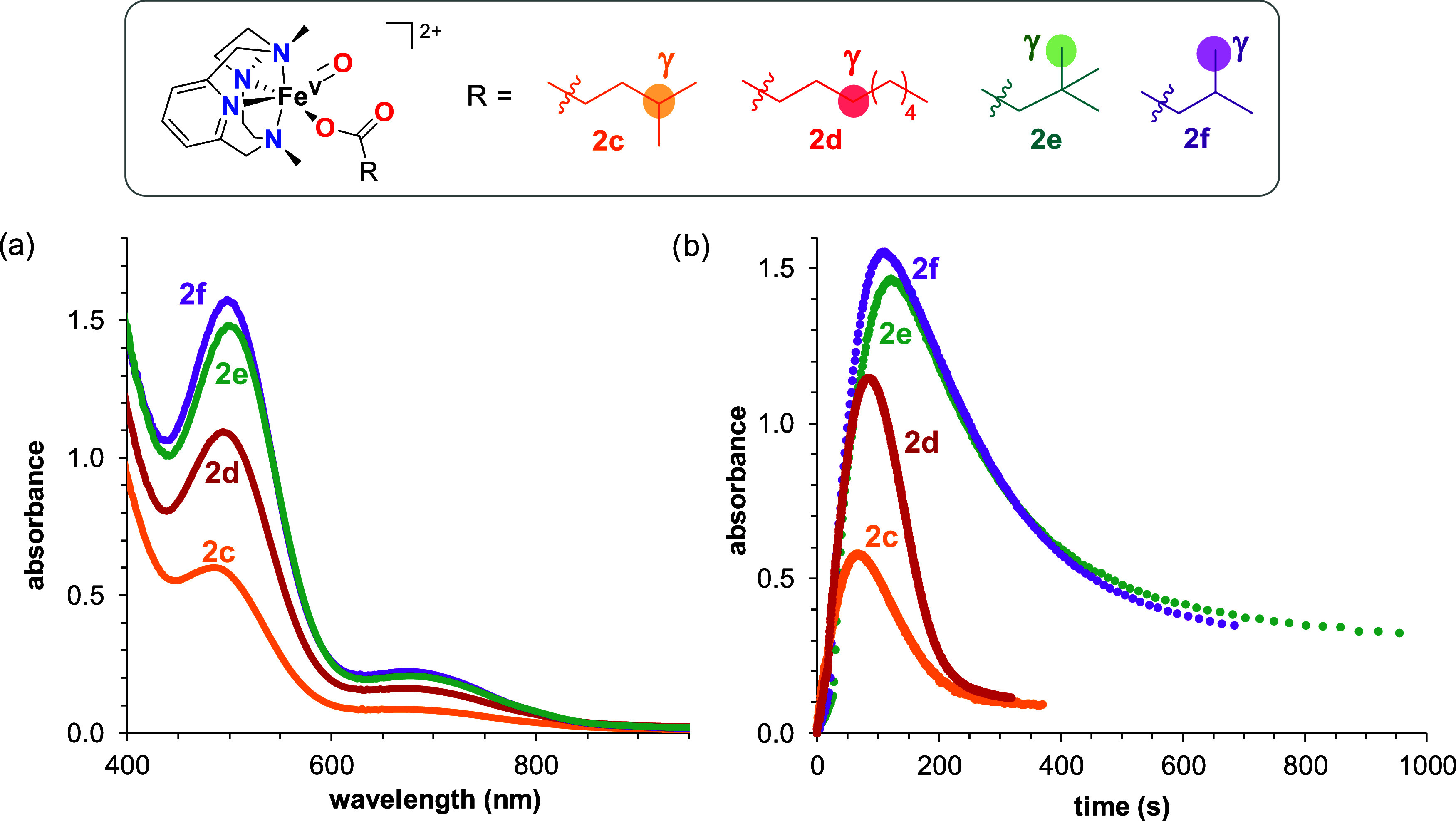
(a) UV–vis spectra of **2c**–**2f** at their maximum accumulation along the reaction of **1** (1 mM) with 4 equiv of the corresponding peracid in acetonitrile at −40 °C. (b) Kinetic trace for the formation and decay of **2c**–**2f** generated by the reaction of **1** (1 mM) with 4 equiv of the corresponding peracid in acetonitrile at −40 °C. Kinetic traces are recorded at 488 nm for **2c**, 497 nm for **2d**, 503 nm for **2e**, and 498 nm for **2f**.

Reaction of **1** toward four different peracids was explored (Scheme 2): 4-methylpervaleric acid (**c**) contains a tertiary C–H bond in the γ position of the peracid functionality; pernonanoic acid (**d**) bears secondary γ C–H bonds, while *tert*-butylperacetic acid (**e**) and isopervaleric acid (**f**) exclusively contain primary C–H bonds in the γ carbon. UV–vis monitoring of the reaction of **1** with 4 equiv of the corresponding peracid in acetonitrile at −40 °C under a N_2_ atmosphere resulted in the disappearance of the absorption band characteristic of **1** and the concomitant increase over a period of less than 3 min of an absorption band centered at around 490 nm characteristic of **2** (488 nm for **c**, 497 nm for **d**, 503 nm for **e**, 498 nm for **f**), which decayed even at this low temperature in the course of a few minutes (Figures 1 and S17–S20). By analogy to previous studies,^31,32^ these chromophoric species are assigned to the corresponding [Fe^V^(O)(OCOR)(PyNMe_3_)]^2+^ (**2c** when R = CH_2_CH_2_CH(CH_3_)_2_, **2d** when R = (CH_2_)_7_CH_3_, **2e** when R = CH_2_C(CH_3_)_3_, and **2f** when R = CH_2_CH(CH_3_)_2_). Both the lifetime and the intensity of their characteristic absorption band were highly dependent on the nature of the peracid used (Figure 1). Thus, **2e** and **2f** bearing primary C–H bonds in the γ position showed the largest accumulation (Abs_max_ ∼ 1.5) and longest half-life times at −40 °C (*t*_1/2_ ∼ 200 s), while **2c** and **2d** with tertiary and secondary γ C–H bonds, respectively, exhibited much lower accumulation (Abs_max_ = 0.6 for **2c** and Abs_max_ = 1.1 for **2d**), and they quickly decomposed (*t*_1/2_ ∼ 80 s for both compounds). Thus, the strength of the C–H bond present in the γ position of the peracid directly affects the accumulation and stability of the corresponding iron(V)-oxo-carboxylato species.

Additional experimental evidence for the formation of the iron(V)-oxo-carboxylato species was obtained by cold-spray ionization mass spectrometry (CSI-MS). MS spectra of species **2c**–**2f** at low temperatures (Figures S22–S29) were apparently quite complex, with the presence of several major peaks, including those of iron(III)-carboxylato, iron(IV)-oxo, and iron(III)-hydroxo compounds. However, signals with mass values and isotopic patterns fully consistent with the iron(V)-oxo-carboxylato species [[Fe^V^(O)(OCOR)(PyNMe_3_)](CF_3_SO_3_)]^+^ and [Fe^V^(O)(OCOR)(PyNMe_3_)]^2+^ could be identified among the most intense ones, similarly to the mass analysis previously described for **2a**.^31^ It should be noted that MS analysis cannot provide unambiguous identification of **2a** because these two mass peaks could also be assigned to the corresponding iron(III)-acylperoxo species [[Fe^III^(OOCOR)(PyNMe_3_)](CF_3_SO_3_)]^+^ or [Fe^III^(OOCOR)(PyNMe_3_)]^2+^, which have exactly the same mass and charge. Interestingly, analogous peaks containing one extra oxygen atom were also significant. These may correspond to the exchange of the carboxylate ligand by the deprotonated peracid (which is present in excess), that is, [[Fe^V^(O)(OOCOR)(PyNMe_3_)](CF_3_SO_3_)]^+^ and [Fe^V^(O)(OOCOR)(PyNMe_3_)]^2+^. MS/MS analyses confirm this formulation as their main fragment corresponds to the iron(IV)-oxo species [[Fe^IV^(O)(PyNMe_3_)](CF_3_SO_3_)]^+^ or [Fe^IV^(O)(PyNMe_3_)]^2+^, indicating the extrusion of a peracid molecule via homolytic Fe–O OOCOR lysis from the parent ion, thus favoring their formulation as iron(V)-oxo species. Interestingly, these peaks quickly disappeared upon warming the solution to room temperature so that they are unequivocally related to the metastable species **2c**–**2f** detected by UV–vis spectroscopy.

### Mössbauer and EPR Spectroscopy of Compounds **2e** and **2f**

**Figure 2:**
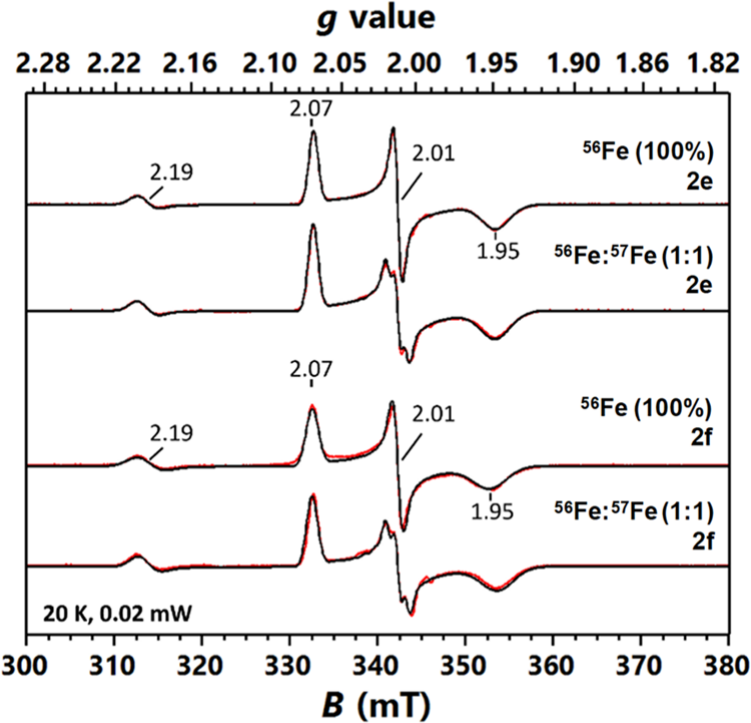
X-band EPR spectra of **2e**, **2f**, and their corresponding ^57^Fe enriched samples (^56^Fe:^57^Fe 1:1) recorded at 20 K in perpendicular mode between 300 and 380 mT. Red lines show experimental spectra, and black lines correspond to their simulations. The spectral simulation parameters are listed in the main text and in Table S2 found in the SI.

According to UV–vis spectroscopy, compounds **2e** and **2f** significantly build up in solution with an Abs_max_ of 1.5 at −40 °C (starting with 1 mM solutions of **1**). Their accumulation could be further increased when these species were generated at −60 °C in an acetone/acetonitrile 3:1 solvent mixture, affording maximum absorbances around 2.0 (Figure S16). Given the apparent higher accumulation of **2e** and **2f** under these reaction conditions, Mössbauer and EPR spectroscopies were recorded. Even though high percentages of the iron(V) species were expected under these optimized conditions for the preparation of these two compounds, both spectroscopies showed that the samples contained roughly 40% Fe^V^ compound. Specifically, the EPR samples containing **2e** or **2f** prepared by using ^56^Fe showed a dominant *S* = 1/2 species having *g* = [2.07, 2.01, 1.95], which represented ∼40% of the iron species in the samples based on spin quantification and sample iron content and could be assigned to **2e** or **2f** (Figure 2). The *g* values of these species are essentially identical to those of **2b** published previously,^32^ thus suggesting they also originate from an iron(V) species. To provide further support for this assignment, we measured EPR data on the samples prepared using a 1:1 ^56^Fe:^57^Fe 1:1. The ^57^Fe nuclear hyperfine splitting was clearly observed at the *g* = 2.01 resonance, corresponding to the magnitude of a principal *A* value as |*A*_*g*=2.01_| = 52 MHz for **2e** or 57 MHz for **2f** based on spectral simulations. This *A* value is very similar to that previously determined for **2b** (|*A*_*g*=2.01_| = 62 MHz).^32^ Overall, the EPR spectral features of **2e** and **2f** are highly similar to those of **2b**, thus confirming that **2e** and **2f** are two new iron(V) species supported by the PyNMe_3_ ligand. In addition to the major *S* = 1/2 iron(V) species, a minor *S* = 1/2 having *g* = [2.20, 2.20, 1.93] in the sample containing **2e** or *g* = [2.20, 2.20, 1.99] in the sample containing **2f** was also observed, which represented <5% of the total iron species in the sample. This minor species was also observed in the previous study and could be assigned to a low-spin iron(III)-acylperoxo species. Lastly, a *g* = 4.3 signal, corresponding to high-spin iron(III) species, was also observed (Figure S30). To solidify the assignment that **2e** and **2f** are iron(V) species, we also performed Mössbauer analysis. By using the previously published Mössbauer parameters of **2b**,^32^ the Mössbauer data from the samples containing **2e** or **2f** can be reasonably simulated to reveal that ∼45% of total iron in the samples were represented by iron(V) species with an isomer shift δ ∼ −0.06 mm/s (see the SI and Figure S31 for a detailed discussion).

### Intramolecular γ-Lactonization

The ability of **2c**–**2f** to carry out intramolecular oxidation of the carboxylate ligand was studied by analyzing the oxidized products formed upon decomposition of **2c**–**2f**. After the full decay of the ∼490 nm absorption band, the reaction mixture was quenched at −40 °C with the addition of 40% aqueous sodium bisulfite and analyzed by gas chromatography (see the SI for experimental details). Interestingly, 2 TON of the γ-lactone derived from 4-methylpervaleric acid were obtained after the self-decay of **2c** at −40 °C, and 1 TON of γ-nonalactone was obtained in the case of **2d** (Table S1). Turnover numbers increased when 8 equiv (instead of 4 equiv) of peracid were used for the generation of **2** giving 5 TON of lactone from **2c** and 2 TON from **2d**. Importantly, no other organic products, aside from the corresponding carboxylic acid, were identified in the reaction mixtures, and blank experiments in the absence of Fe showed no lactone product. Disappointingly, decomposition of **2e** and **2f** at −40 °C did not lead to the formation of γ-lactones, indicating that under these conditions, the iron(V)-oxo-carboxylato compound is not able to cleave primary C–H bonds. Notwithstanding, when the reaction of **1** with **e** or **f** was carried out at room temperature in acetonitrile or at −20 °C in a fluorinated solvent such as 2,2,2-trifluoroethanol, significant amounts (0.2–1.0 TON) of γ-lactones were observed (Table S1). Even though under these reaction conditions accumulation of **2e** and **2f** is rather poor, these are the most likely species involved in the observed lactonization reactions, suggesting that under the appropriate reaction conditions, the iron(V)-oxo-carboxylato species can afford the activation of strong primary C–H bonds. Although the combination of **1** and peracids constitutes a rather poor catalytic system with very modest turnover numbers, the observed γ-lactonization upon decomposition of trapped species **2c**–**2f** demonstrates that these compounds serve as models for the metal(V)-oxo-carboxylato species widely postulated to be the key reactive intermediates in the oxidation/lactonization of C–H bonds catalyzed by Fe and Mn complexes.

**Figure 3:**
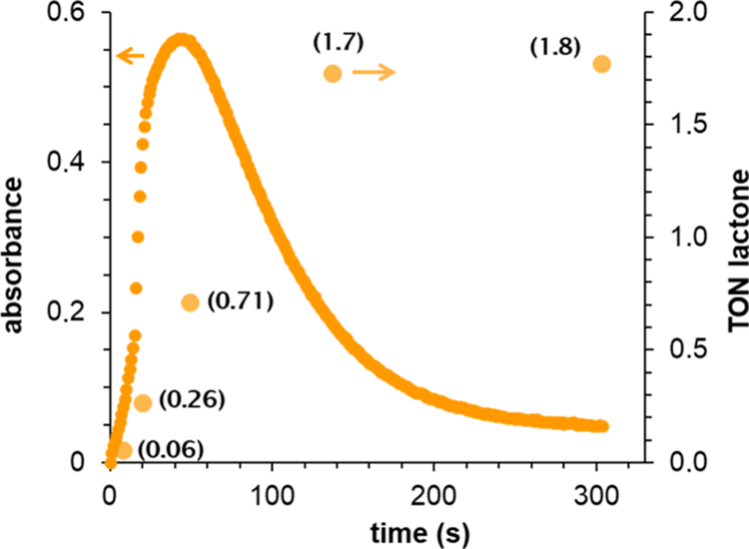
Kinetic trace at 488 nm corresponding to the formation and decay of **2c** obtained by the reaction of **1** with 4 equiv of **c** in acetonitrile at −40 °C (solid line) along with the amount of γ-lactone detected at different reaction times during the formation/decay of **2c** (dots). Similar results are obtained with **2d** (see Figure S21 in the SI).

In order to confirm that the lactonization reaction is directly related to the iron(V)-oxo-carboxylato species and does not originate from a background reaction, the formation of the γ-lactone over time was monitored along with the formation and decay of **2c** and **2d** (Figures 3 and S21). At the initial stages of the formation of the 490 nm band characteristic of **2c** and **2d**, γ-lactone was already present in the reaction mixture, and its amount concomitantly increased during the accumulation and decay of the oxoiron(V) species. For example, in the case of **2c**, at the point of its maximum accumulation, 0.71 TON of lactone (40% of the total) had already been formed. This value increased up to 1.7 TON (94% of the total) when 70% of the absorption band of **2c** had already decayed. Once **2c** and **2d** had completely disappeared, lactone formation was also stopped. As these species are unstable, their decomposition must occur from the initial stages of the reaction, which agrees with the presence of lactone from the very beginning of the reaction. These experiments show that the formation and decay of **2c** and **2d** correlate with lactone formation, so that the iron(V)-oxo-carboxylato species is directly involved in the generation of this product.

**Figure 4:**
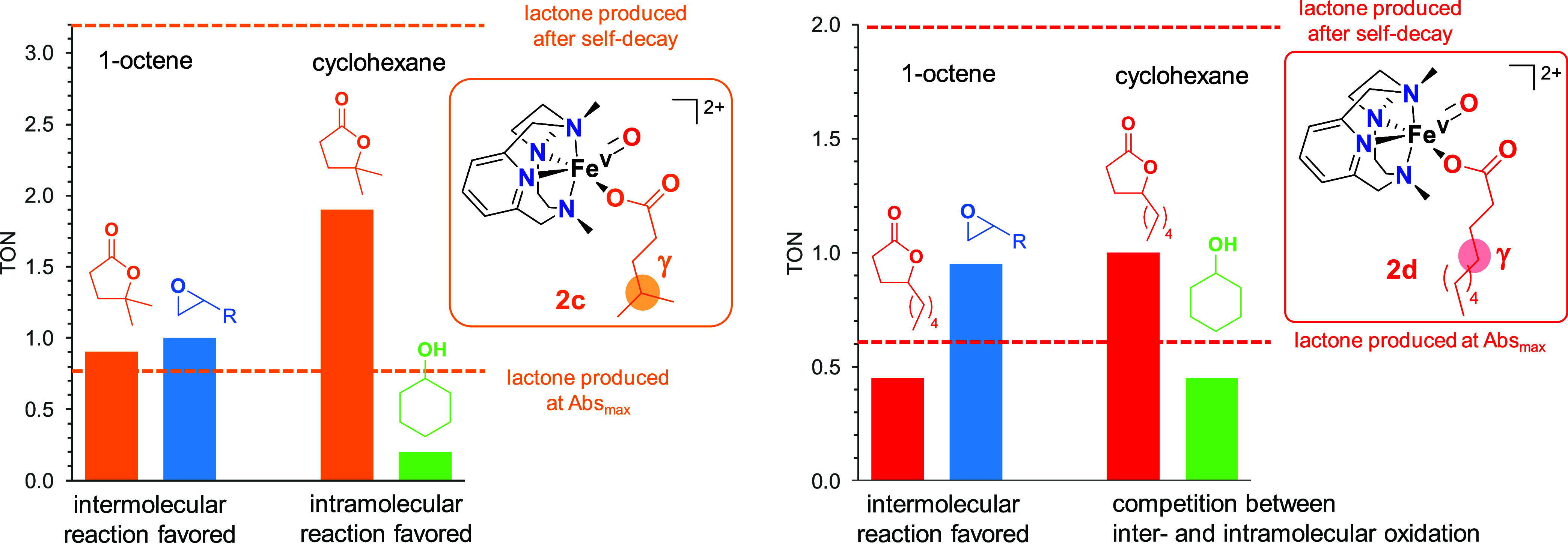
Distribution of organic products after decomposition of **2c** (left) and **2d** (right) in the presence of 1-octene or cyclohexane.

We have previously shown that the iron(V)-oxo-acetato species **2a** is able to efficiently carry out hydroxylation of C–H bonds and epoxidation of olefins.^31,34^ Thus, further evidence for the involvement of **2c** and **2d** in the lactonization reaction was gathered through competition experiments with external substrates. In order to do so, **2c** and **2d** were generated, and 100 equiv of cyclohexane or 20 equiv of 1-octene were added at the maximum accumulation of these species. As expected, the addition of 1-octene to **2c** and **2d** caused the instantaneous decay of their characteristic absorption band at ∼490 nm and resulted in the generation of around 1 TON of the corresponding epoxide product in both cases (Figure 4). Interestingly, the amount of γ-lactone stopped as soon as 1-octene was added, so that only ∼1 TON of γ-lactone was detected for **2c** and ∼0.5 TON for **2d**, corresponding to the amount of this product produced just before the addition of the alkene substrate. Clearly, the iron(V)-oxo-carboxylato species preferentially oxidizes the external olefin rather than the intramolecular γ C–H bond. The situation was different when cyclohexane was used as an external substrate. In both **2c** and **2d**, γ-lactone production continued after the addition of cyclohexane, albeit significant amounts of cyclohexanol were observed, specially in the case of **2d** (around 0.5 TON). This suggests that for **2d**, there is a competition between the oxidation of the secondary C–H bond of the cyclohexane substrate and the intramolecular oxidation of the secondary C–H bond in the γ-position of the peracid. Instead, for **2c**, oxidation of the secondary C–H bonds of cyclohexane is minimal, and the system rather prefers the intramolecular oxidation of the tertiary C–H bond in the γ-position of the peracid. This is in agreement with the weaker bond dissociation energy of tertiary C–H bonds than secondary ones. Overall, these competition experiments indicate that the same species, namely, oxoiron(V)-carboxylato, is responsible for the intra- and intermolecular oxidation reactions.

### Experimental Mechanistic Studies on the γ-Lactonization

**Scheme 3:**
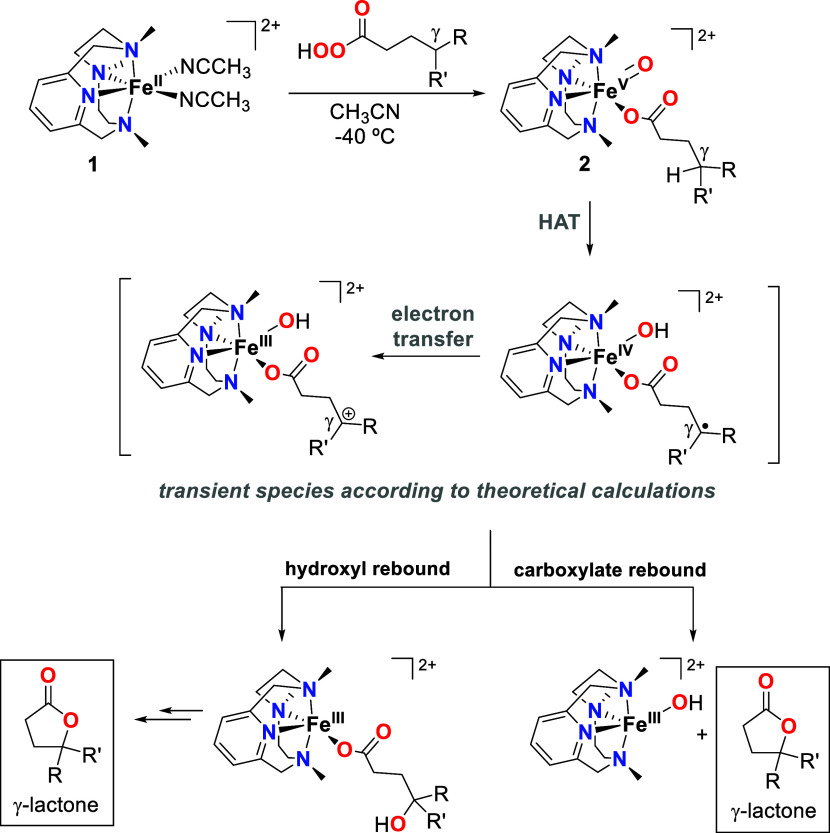
Proposed Mechanism for the Lactonization Reactions Carried Out by the Iron(V)-oxo-carboxylato Species (**2**)

The aliphatic C–H lactonization most likely occurs through a mechanism analogous to that of C–H hydroxylation of external alkane substrates carried out by the iron(V)-oxo-carboxylato species (Scheme 3). Thus, we hypothesize that the intramolecular reaction occurs through a HAT step at the γ C–H bond, followed by a fast rebound of the newly formed alkyl radical with either the hydroxyl or the carboxylato ligand at the iron center, affording the corresponding γ-lactone. Of note, theoretical calculations (see below) indicate that the C–H cleavage event might not be a canonical HAT. Instead, the process is globally described as an asynchronous hydride transfer that consists of an initial HAT followed by an electron transfer prior to the formation of the final lactone.

**Scheme 4:**
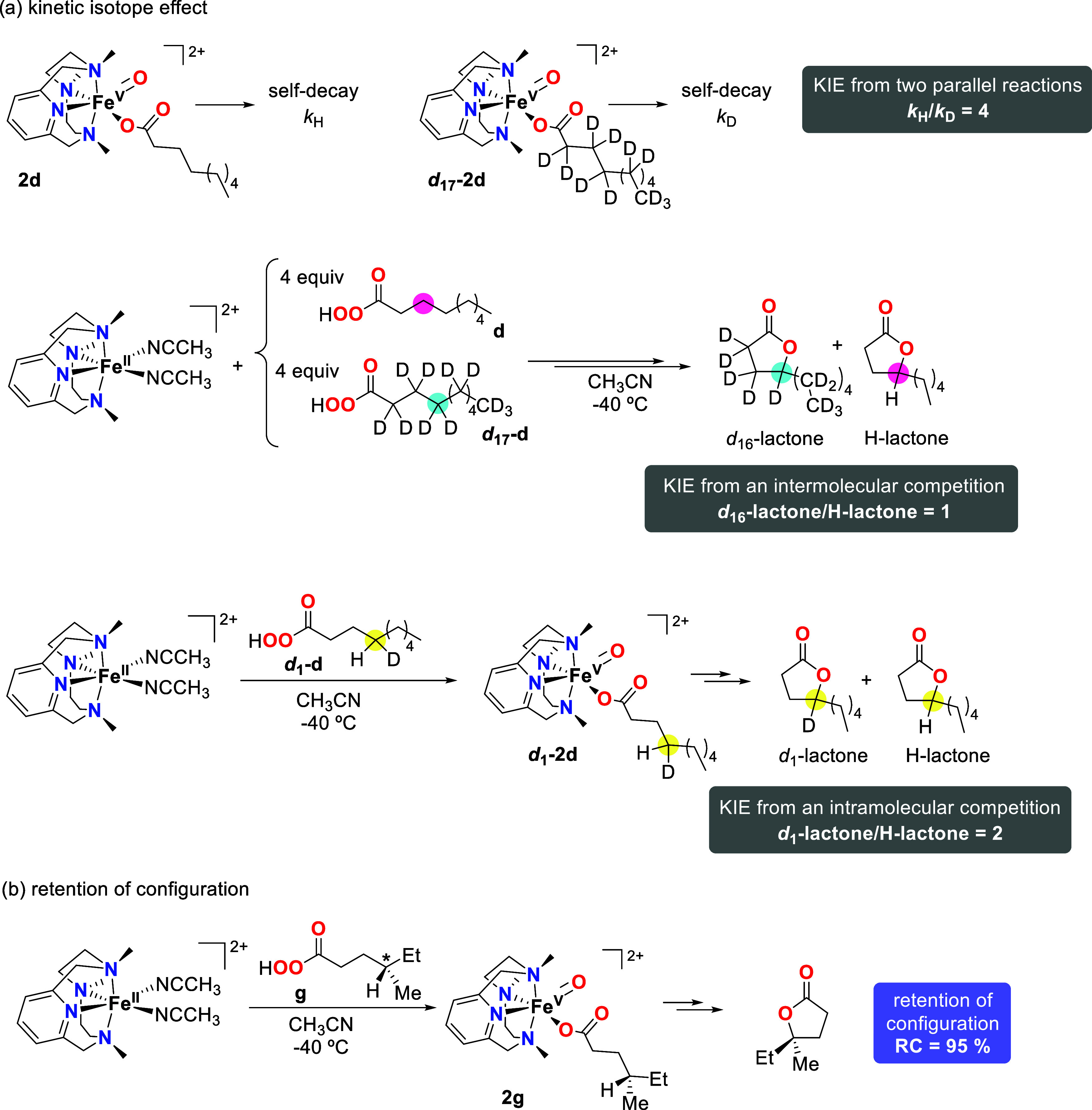
Mechanistic Studies to Unravel the Reaction Mechanism for the Lactonization Reactions (a) Summary of three different experiments to measure the kinetic isotope effect (KIE) in the lactonization reaction. (b) Use of peracid **g** containing a chiral carbon center in the γ-position for the generation of the corresponding iron(V)-oxo-carboxylato species (**2g**) to determine the retention of configuration along the lactonization reaction.

With the objective of obtaining more information about the C–H cleavage step, kinetic isotope effects (KIE) were measured (Scheme 4a). First, KIE was determined by measuring the observed decay rate of **2d** (*k*_H_) and its deuterated counterpart ***d***_**17**_**-2d** (*k*_D_), generated by the reaction of **1** with pernonanoic-*d*_17_ acid (***d***_**17**_**-d**) (Figure S33). The ratio between the observed decay rates of the two parallel reactions (*k*_H_/*k*_D_) afforded a KIE value of 4, which agrees with the KIE values previously reported for C–H oxidations occurring through the mediation of oxoiron(V) species.^37^ Second, a KIE was determined by an intermolecular competition in which **1** reacted with 4 equiv of **d** and ***d***_**17**_**-d** at the same time. Product analyses after the self-decay of the formed iron(V)-oxo-carboxylato species showed equimolar quantities of deuterated and nondeuterated lactones, affording a KIE of 1. The lack of KIE in this intermolecular competition experiment suggests that an irreversible coordination of the peracid to the iron center takes place before the C–H breaking event. Thus, both **d** and ***d***_**17**_**-d** irreversibly bind to the metal, and no distinction between the deuterated and nondeuterated peracid is made. Finally, a KIE was determined by an intramolecular competition between activation of a C–H and a C–D bond in a single substrate. In particular, pernonanoic acid singly deuterated at the γ-position (***d***_**1**_**-d**) was used for the generation of **2** (see the SI for details of its synthesis). The corresponding iron(V)-oxo-carboxylato species (***d***_**1**_**-2d**) was generated, and the lactone products obtained after its self-decay were analyzed (Figure S35). The ratio between deuterated lactone (cleavage of the C–H bond) and the nondeuterated one (cleavage of the C–D bond) afforded an intramolecular KIE of 2. Even though this value is significantly lower than the KIE of 4 determined using observed rate constants (see above) and the origin of these differences is not understood at the moment, both values suggest that the C–H cleavage is the rate-determining step of the reaction.

Analysis of the reaction rate for the self-decay of **2d** as a function of the temperature affords activation energies for the rate-determining step (see the Eyring plot in Figure S32). Thus, an activation barrier with a relatively large activation enthalpy (Δ*H*^‡^ = 15 kcal·mol^–1^) and a very small and negative activation entropy (Δ*S*^‡^ = −2 cal·K^–1^ mol^–1^) was determined. The low Δ*S*^‡^ agrees well with an intramolecular process with a preorganized reactant so that reorganization is minimized in the transition state. In line with this result, much larger Δ*S*^‡^ values were previously determined for the intermolecular processes involving the reaction of **2a** with cyclohexane (Δ*S*^‡^ = −18 cal·K^–1^ mol^–1^)^31^ or 1-octene (Δ*S*^‡^ = −21 cal·K^–1^ mol^–1^).^34^

In order to evaluate the lifetime of the species formed after C–H cleavage, the generation of the oxoiron(V) species was carried out using (*S*)-4-methylperhexanoic acid (**g**), which contains a chiral tertiary carbon in the γ position (Scheme 4b). Analysis of the organic products after the self-decay of **2g** (Figure S14) showed the production of 1.4 TON of the corresponding lactone with 95% retention of configuration. This result indicates that the species formed after the C–H breaking event is very short-lived and does not have enough time to epimerize, so that the ligand (either hydroxide or carboxylate) rebound occurs very fast. Such a fast rebound has been previously observed for the intermolecular reactions carried out by **2a**(31,35) and also in iron and manganese-catalyzed hydroxylation reactions that presumably occur through the mediation of oxometal(V) species as key oxidants.^38−44^

**Scheme 5:**
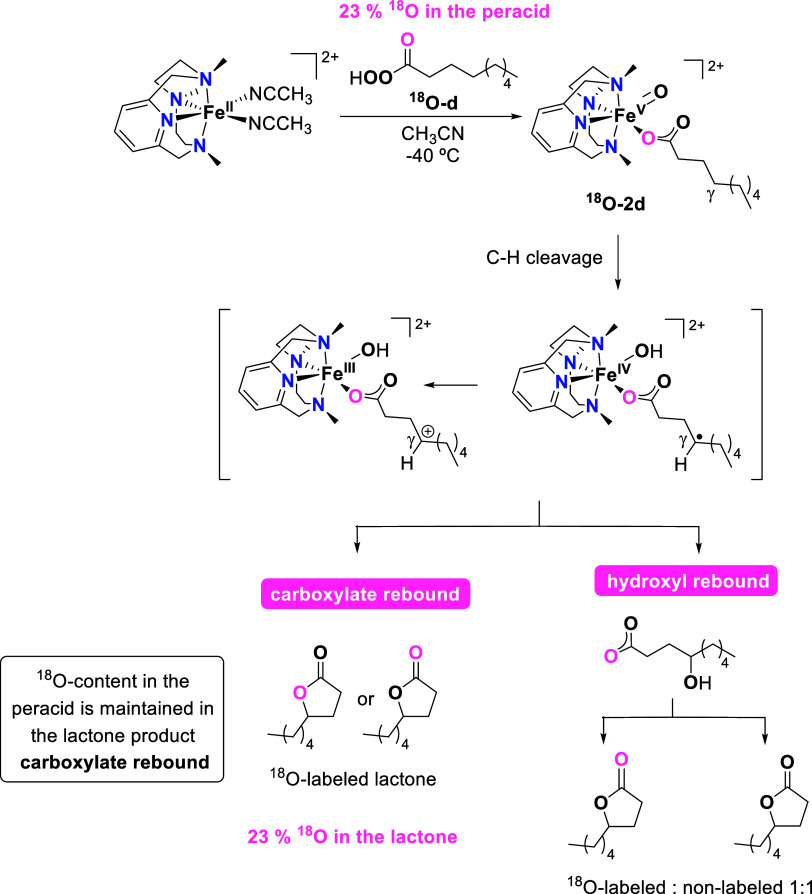
^18^O-labeling Experiment Designed to Distinguish between Carboxylate or Hydroxyl Rebound after C–H Cleavage

After C–H cleavage, the γ-carbon can undergo rebound with either the hydroxyl or carboxylate ligand. In order to distinguish between these two pathways, we synthesized pernonanoic acid with 23% ^18^O-label in the oxygen atom of the carbonyl group, C_8_H_17_C^18^OOOH (^**18**^**O-d**) (see the SI for its synthesis). Reaction of **1** with this peracid in CH_3_CN at −40 °C led to the generation of the corresponding iron(V)-oxo-carboxylato species (^**18**^**O-2d**), as ascertained by UV–vis spectroscopy (Scheme 5). Upon self-decay, this species afforded γ-nonalactone with 23% ^18^O-content (Figure S37). As the level of ^18^O-labeling of the peracid is maintained in the lactone product, this is a clear indication that a carboxylate rebound occurs after C–H cleavage. If the hydroxyl rebound were the preferred pathway, the ^18^O-content of the lactone product would be half of that of the peracid (Scheme 5). Interestingly, previous reports in the literature on manganese- and iron-catalyzed γ-lactonizations of alkyl carboxylic acids using H_2_O_2_ as an oxidant show that the preference for hydroxyl or carboxylate rebound is highly dependent on the particular system. Thus, ^18^O-labeling experiments in an iron-catalyzed γ-lactonization of tertiary C–H bonds show that the hydroxyl rebound is the favored pathway,^12^ while in an equivalent enantioselective manganese-catalyzed functionalization of methylenic units, the two pathways are in competition.^19^ Finally, the lactonization of primary C–H bonds by a Mn catalyst follows a mechanism in which the primary carbon-centered radical species is rapidly trapped by the carboxylate ligand without the intermediacy of a γ-hydroxy acid.^20^

### Theoretical Calculations

**Figure 5:**
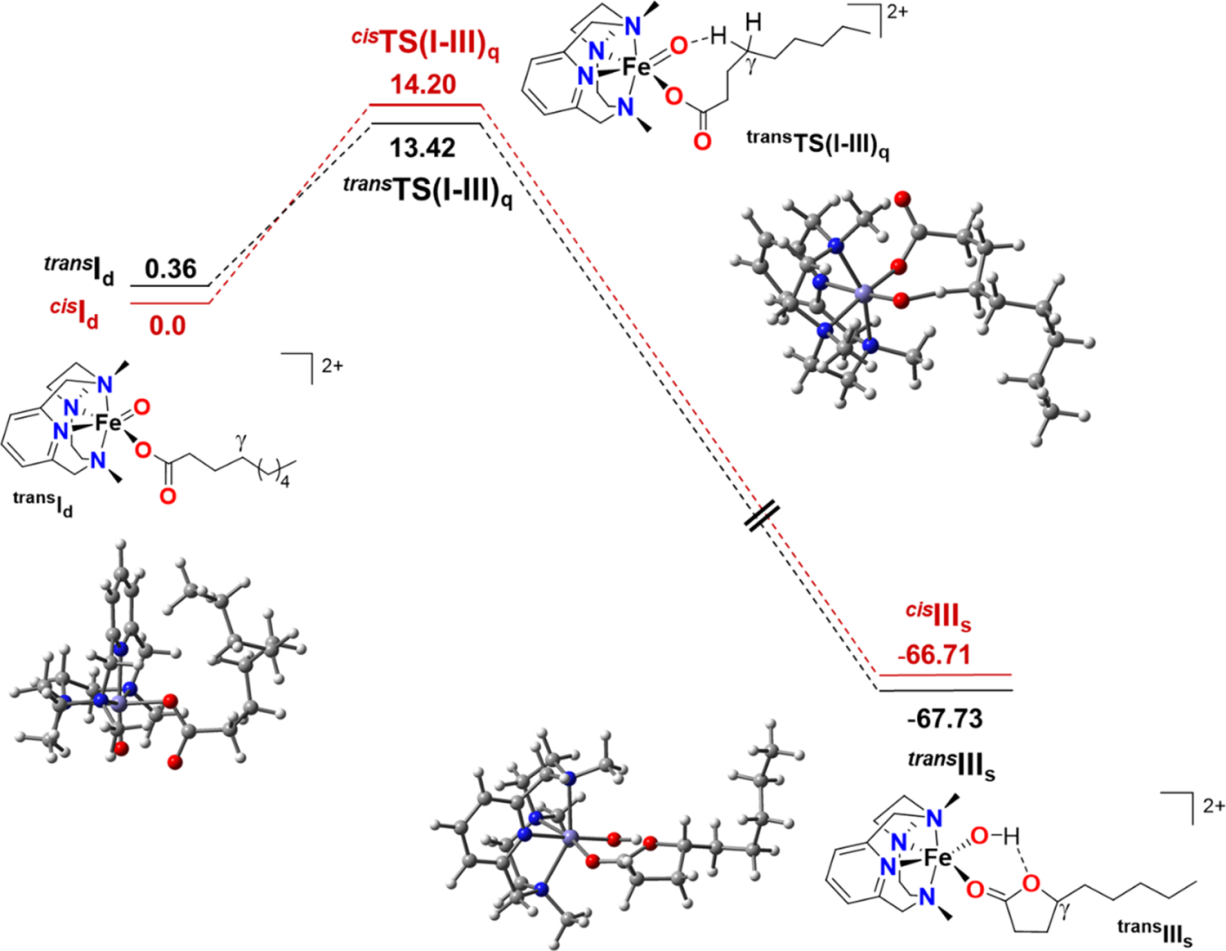
Reaction profile for the γ-C–H lactonization of pernonanoic acid computed at the B3LYP-D3BJ/Def2TZVP/SMD(acetonitrile)//B3LYP-D3BJ/Def2SVP/SMD (acetonitrile) level of theory for compound **2d**. Gibbs energies are given in kcal·mol^–1^. Subscripts d, q, and s represent spin states *S* = 1/2, *S* = 3/2, and *S* = 5/2, respectively. The depicted compounds correspond to the *trans* isomers.

DFT calculations at the B3LYP-D3BJ/Def2TZVP/SMD(acetonitrile)//B3LYP-D3BJ/Def2SVP/SMD(acetonitrile) level of calculation were undertaken to analyze the activation of the γ-C–H bond carried out by the iron(V)-oxo-carboxylato species (see Figure 5 and the SI for more details). Calculations were performed for **2d** considering that this species can exist as two geometrical isomers as previously observed for structurally related iron(IV)-oxo species:^45^ one isomer contains the oxo group *trans* with respect to the pyridine ring, while in the other isomer, the oxo ligand and the pyridine moiety are in a relative *cis* disposition. DFT calculations predict that the ground states of both **2d** isomers (^**cis**^**I**_**d**_ and ^**trans**^**I**_**d**_) have a doublet spin multiplicity, in agreement with experimental results.^31,32^ The global C–H oxidation at the γ-carbon carried out by the iron-oxo moiety requires overcoming a doublet → quartet spin-crossing and a Gibbs energy barrier (Δ*G*^‡^) of 14.2 kcal·mol^–1^ for ^**cis**^**I**_**d**_, and 13.1 kcal·mol^–1^ for ^**trans**^**I**_**d**_, the latter being 0.4 kcal·mol^–1^ higher in Gibbs energy than the *cis* isomer. More specifically, DFT calculations predict that the Gibbs energy barriers are due to large activation enthalpies Δ*H*^‡^ (13.8 kcal·mol^–1^ for ^**cis**^**TS(I–III)**_**q**_ and 13.1 kcal·mol^–1^ for ^**trans**^**TS(I–III)**_**q**_) and small activation entropies Δ*S*^‡^ (−1.9 cal·mol^–1^·K^–1^ for ^**cis**^**TS(I–III)**_**q**_ and +0.2 cal·mol^–1^·K^–1^ for ^**trans**^**TS(I–III)**_**q**_). These DFT results are in good agreement with the corresponding experimental values of 15.5 kcal·mol^–1^, 15.1 kcal·mol^–1^, and −2 cal·mol^–1^·K^–1^ for the Gibbs energy barrier, activation enthalpy, and activation entropy, respectively. In the ^**trans**^**TS(I–III)**_**q**_ and ^**cis**^**TS(I–III)**_**q**_ transition states, the substrate approaches sideways (the transition state Fe–O–H angle is 114.3 and 115.3°, respectively), and therefore the reaction proceeds via a π channel, i.e., through the π_xz_* Fe–O molecular orbital.^46−48^

Interestingly, the spin density measured in ^**cis/trans**^**TS(I–III)**_**q**_ is in agreement with a hydrogen atom transfer (see Table S4). For the *trans* isomer, the IRC path connects the ^**trans**^**TS(I–III)**_**q**_ with a nonstable intermediate electronic structure that corresponds to the product of a canonical HAT, with a spin density of 0.9 on the γ-carbon and 2.0 on the Fe, which nicely agrees with an *S* = 1 Fe^IV^–OH complex and a radical γ-carbon (^**trans**^**IRC1**_**q**_, Tables S4 and S8). However, the IRC progresses downhill 21 kcal·mol^–1^ from ^**trans**^**IRC1**_**q**_ to another nonstable electronic structure with a spin density of 2.8 in the Fe and a positive charge of 0.9 in the carbon chain, which corresponds to a *S* = 3/2 Fe^III^–OH compound with a cation in the alkyl chain (^**trans**^**IRC2**_**q**_, Tables S4 and S8). Although ^**trans**^**IRC2**_**q**_ is not a stable minimum, we have found stable conformers with the same electronic structure and a slightly lower energy (^**trans**^**II**_**q**_). Therefore, our computational results suggest that the C–H cleavage might be globally described as an asynchronous hydride transfer, which involves an initial HAT followed by an electron transfer from the γ-carbon to the metal. Such a mechanistic scenario has some precedent in the literature, and it has been proposed for C–H oxidation in cyclopropane-containing hydrocarbons catalyzed by manganese complexes.^49^ Finally, the IRC pathway for the *trans* isomer evolves downhill from ^**trans**^**IRC2**_**q**_ to the strongly exergonic formation of the experimentally detected γ-lactone coordinated to the iron(III) center (^**trans**^**III**_**q**_) through a carboxylate rebound. As expected, the corresponding *S* = 5/2 species is more stable; therefore, the formation of the γ-lactone involves a spin-crossing to the ^**trans**^**III**_**s**_ compound. The barrierless formation of the final lactone through a carboxylate rebound is in full agreement with the ^18^O-isotope labeling experiments and the experimentally observed retention of the configuration of the γ-carbon in the studied lactonization reactions (see above). Overall, these results align with the very low barrier for a carboxylate rebound previously calculated for a manganese complex that catalyzes the γ-lactonization of unactivated primary C–H bonds.^20^ Therefore, all of the computational and experimental shreds of evidence indicate that the carboxylate rebound process has no energy barrier or an energy barrier negligible with respect to the C–H cleavage, in complete agreement with the previous literature.^20^

For the *cis* isomer, the IRC profile from the ^**cis**^**TS(I–III)**_**q**_ structure toward the lactone product also presents two plateaus related to two intermediate nonstable electronic structures before reaching the final product (^**cis**^**IRC1**_**q**_ and ^**cis**^**IRC2**_**q**_, Tables S4 and S8). Again, ^**cis**^**IRC1**_**q**_ corresponds to the product of a canonical HAT, while the less energetic one (^**cis**^**IRC2**_**q**_) corresponds to a Fe^III^–OH compound with a cation in the alkyl chain. We have optimized stable conformers with the same electronic structure and slightly lower energy than that of ^**cis**^**IRC2**_**q**_ (^**cis**^**II**_**q**_). Interestingly, for the *cis* isomer, the IRC evolves downhill to the final lactone product (^**cis**^**III**_**s**_) through a hydroxyl rebound instead of a carboxylate rebound. Of note, the ^**cis**^**TS(I–III)**_**q**_ is 0.8 kcal·mol^–1^ higher in energy than ^**trans**^**TS(I–III)**_**q**_. In addition, the ^18^O-isotopic labeling experiments described above discard the implication of this path.

## Conclusions

In this work, a series of iron(V)-oxo-carboxylato species (**2**) containing carboxylate ligands of different natures has been generated by the reaction of the ferrous complex **1** and peracids, and they have been characterized spectroscopically. Interestingly, their self-decay leads to the intramolecular oxidation of the C–H bond in the γ-position of the alkyl chain of the carboxylate group, affording the corresponding γ-lactones. This decomposition pathway gives a rationale for the observed influence of the degree of substitution of the γ-carbon on the accumulation and stability of **2**, so that weaker secondary and tertiary C–H bonds in this position afford less stable iron(V)-oxo-carboxylato species than primary C–H bonds in this position. By tracing the lactone production over time and through a series of competition experiments with external substrates, the direct implication of **2** in the lactonization process is demonstrated.

The mechanism of the lactonization reaction was disclosed by theoretical and experimental analyses. The first step corresponds to an intramolecular rate-determining C–H cleavage in **2**, which, according to our theoretical calculations, might be globally described as an asynchronous hydride transfer that consists of an initial HAT followed by an electron transfer and the barrierless formation of the final lactone. This process involves the formation of a nonstable iron(IV)-hydroxo species with a radical γ-carbon, which then leads to the exergonic formation of an iron(III)-hydroxo species with a positive charge on the alkyl chain. Inter- and intramolecular KIE experiments fully agree with this picture, and the intramolecular nature of the C–H cleavage is fully consistent with the low Δ*S*^‡^ determined from an Eyring analysis and DFT calculations. The second step of lactonization is a fast carboxylate rebound process, as ascertained by ^18^O-labeling experiments and supported by theoretical calculations. This fast rebound translates into an almost complete retention of configuration when chiral γ-carbons are present in the carboxylate ligand.

Overall, this work constitutes the first example of a well-identified iron(V)-oxo-carboxylate species that performs a selective intramolecular γ-lactonization process, thus serving as a model for the widely postulated metal(V)-oxo-carboxylato species proposed to be the key reactive intermediates in the γ-oxidation/lactonization of C–H bonds catalyzed by iron and manganese complexes. Trapping and studying the nature and properties of these key reaction intermediates may serve to further improve catalyst design and increase the efficiency of these synthetically relevant C–H oxidation processes.

## Supporting Information Available

Materials and methods, synthesis of peracids, general procedure for the generation of **2c**–**2g**, reaction with external substrates and product analyses, CSI-MS experiments, Mössbauer and EPR spectroscopy of **2e** and **2f**, Eyring plot, KIE experiments, and synthesis of other organic products and additional information on theoretical calculations (PDF)
