# Supplementary material for: Intramolecular C–H Oxidation in Iron(V)-oxo-carboxylato Species Relevant in the γ-Lactonization of Alkyl Carboxylic Acids
Source: ACS Catal. 2024 Sep 11;14(18):14183–94. doi: 10.1021/acscatal.4c01258 (PMC11420956; doi:10.1021/acscatal.4c01258)
Supplement: Supplementary file 1 — cs4c01258_si_001.pdf [file cs4c01258_si_001.pdf]

Intramolecular C–H oxidation in iron(V)-oxo-carboxylato species relevant in the γ-lactonization of alkyl carboxylic acids

Andrea Álvarez-Núñez,[a] Rudraditya Sarkar,[a,c] Valeria Dantignana,[a] Jin Xiong,[b] Yisong Guo,[b] Josep M. Luis,[a]* Miquel Costas [a]* Anna Company[a]*

[a] Institut de Química Computacional i Catàlisi (IQCC), Departament de Química, Universitat de Girona, C/ Mª Aurèlia Capmany 69, 17003 Girona, Catalonia, Spain.
E-mail: josepm.luis@udg.edu; miquel.costas@udg.edu; anna.company@udg.edu

[b] Chemistry Department, Carnegie Mellon University, Pittsburgh, PA 15213, USA

[c] Department of Chemistry, School of Science, Gandhi Institute of Technology and Management (GITAM), Hyderabad-502329, India

Table of contents

1. Materials and methods 3

2. Synthesis of the peracids..4

3. Generation of the [Fe^{V}(O)(OC(O)R)($PyNMe_3$)]$^{2+}$, reaction with external substrates and product analyses 10

3.1. Relevant GC-FID and GC-MS chromatograms..12

3.2. Generation of 2e and 2f at -60 ºC 17

3.3. Generation of 2c-2f at -40 ºC 17

3.4. Monitoring lactone formation over time for 2d..19

4. Cryospray-MS experiments (CSI-MS)...20

5. Mössbauer and EPR spectroscopy of compounds 2e and 2f..............................28

6. Eyring plot 30

7. Isotope experiments 31

7.1. KIE determined from decay rates of two parallel reactions.....................................31

7.2. KIE determined from an intramolecular competition 31

7.3. ^{18}O labelling experiments 33

8. Synthesis of other organic products...35

8.1. Synthesis of nonanoic-4-d_1 acid 35

8.2. Synthesis of 5-ethyl-5-methyldihydrofuran-2-one..44

8.3. Synthesis of (*S*)-4-methylhexanoic acid 46

8.4. Synthesis of nonanoic-^{18}O acid 47

9. DFT calculations 49

9.1. Computational details for the electronic structure calculations 49

9.2. Spin density..50

9.3. Key geometrical and IR parameters...51

9.4. Electronic energies, thermal and entropic corrections, and Gibbs energies 52

9.5. Cartesian coordinates of the optimized geometries of $^{cis}I_d$, $^{trans}I_d$, $^{cis}I_q$, $^{cis}I_{q,IRC}$, $^{trans}I_q$, $^{cis}I_{s,}$ $^{trans}I_s$, $^{cis}TS(I\text{-}III)_q$, $^{trans}TS(I\text{-}III)_q$, $^{cis}TS(I\text{-}III)_d$, $^{trans}TS(I\text{-}III)_d$, $^{cis}II_d$, $^{trans}II_d$, $^{cis}IRC1_q$, $^{cis}IRC2_q$, $^{cis}II_q$, $^{trans}IRC1_q$, $^{trans}IRC2_q$, $^{trans}II_q$, $^{cis}II_s$, $^{trans}II_s$, $^{cis}TS(II\text{-}III)_s$,$^{cis}III_d$, $^{trans}III_d$, $^{cis}III_q$, $^{trans}III_q$, $^{trans}III_{q,IRC}$, $^{cis}III_s$, and $^{trans}III_s$ 58

10. References 115

1. Materials and methods

Materials. Reagents and solvents used were of commercially available reagent quality. Solvents were purchased from Sigma-Aldrich and Scharlab and used without further purification. Preparation and handling of air-sensitive materials were carried out in a N_2 drybox (Jacomex) with O_2 and H_2O concentrations <1 ppm. $PyNMe_3$ and $[Fe^{II}(CF_3SO_3)_2(PyNMe_3)]$ (which is the precursor of **1**, $[Fe^{II}(PyNMe_3)(NCCH_3)_2]^{2+}$) were prepared following previously described procedures.[1]

Physical methods. NMR experiments were performed on a Bruker Ultrashield ASCEND Nanobay 400 MHz spectrometer. High resolution mass spectra (HR−MS) were recorded on a Bruker MicrOTOF-Q IITM instrument using ESI or Cryospray ionization sources at Serveis Tècnics of the University of Girona. Samples were introduced into the mass spectrometer ion source by direct infusion using a syringe pump and were externally calibrated using sodium triflate. A cryospray attachment was used for CSI−MS (cryospray mass spectrometry). The temperature of the nebulizing and drying gases was set at −60 °C. The instrument was operated in positive ion mode. UV−vis spectroscopy was performed with an Agilent 50 Scan (Varian) UV−vis spectrophotometer with 1 cm quartz cells. Low temperature control was achieved with a cryostat from Unisoku Scientific Instruments, Japan. GC product analyses were performed on an Agilent 7820A gas chromatograph equipped with an HP-5 capillary column 30 m x 0.32 mm xzaa< 0.25 µm and a flame ionization detector. Enantiomer resolution was achieved using J&W CYCLOSIL-B column. GC-MS analysed were performed on an Agilent 7890A gas chromatograph equipped with an HP-5 capillary column interfaced with an Agilent 5975C mass spectrometer.

2. Synthesis of the peracids

Caution: Organic peracids are thermally sensitive and can spontaneously explode when drying. We have experienced several cases of spontaneous decomposition. Preparation of these compounds in small amounts and protecting measures are needed.

General procedure.[2] The appropriate carboxylic acid (100 mg) was mixed with methanesulfonic acid (~212 μL, 4.7 equiv) at room temperature. Then H_2O_2 50 % (~54 μL, 1.4 equiv) was added dropwise to the mixture, which was subsequently stirred at 40 ºC for 4.5 hours. Afterwards, ice was added along with a saturated aqueous solution of $(NH_4)_2SO_4$ (1 mL) and the mixture was extracted with dichloromethane (2 x 1 mL). The combined organic layers were washed with an aqueous solution of $(NH_4)_2SO_4$ (2 x 2 mL). Afterwards the organic fraction was dried with $MgSO_4$, and the solution was filtered through Celite©. Finally, the solution was dried using a stream of N_2 affording a transparent oil corresponding to the desired peracid. The purity of the peracid was established by ^{1}H-NMR, by comparing the integrals of the peracid peaks with those of the starting carboxylic acid. The peracid products are thermally sensitive, so that they need to be stored at -84 ºC to minimize decomposition.

4-methylpervaleric acid (c): 4-methylvaleric acid was used as starting carboxylic acid. Purity: 73 %; Yield: 78 %. ^{1}H-NMR ($CDCl_3$, 400 MHz, 298 K) δ, ppm**:** 11.36 (s, 1H), 2.43 (t, 2H), 1.65-1.54 (m, 3H), 0.93-0.90 (m, 6H).

Pernonanoic acid (d): Nonanoic acid was used as starting carboxylic acid. Purity: 85 %; Yield: 85 %. ^{1}H-NMR ($CDCl_3$, 400 MHz, 298 K) δ, ppm**:** 11.36 (s, 1H), 2.42 (t, J = 8 Hz, 2H), 1.73-1.62 (m, 2H), 1.36-1.22 (m, 10H), 0.88 (t, J = 8 Hz, 4H).

***tert*-Butyl peracetic acid (e):** *tert*-Butyl acetic acid was used as starting carboxylic acid. Purity: 90 %; Yield: 61 %. ^{1}H-NMR ($CDCl_3$, 400 MHz, 298 K) δ, ppm**:** 11.40 (s, 1H), 2.31 (s, 2H), 1.06 (9H).

Isopervaleric acid (f): Isopervaleric acid was used as starting carboxylic acid. Purity: 83 %, Yield: 73 %. ^{1}H-NMR ($CDCl_3$, 400 MHz, 298 K) δ, ppm**:** 11.38 (s, 1H), 2.31 (d, J = 4 Hz, 2H), 2.25-2.10 (m, 1H), 1.01 (d, J = 4 Hz, 6H).

Pernonanoic acid-d_{17} (d_{17}-d): Nonanoic acid-d_{17} was used as starting carboxylic acid. Purity: 100 %; Yield: 67 %. ^{1}H-NMR ($CDCl_3$, 400 MHz, 298 K) δ, ppm**:** 11.38 (s, 1H).

Pernonanoic-4-d_1 acid (d_1-d): Nonanoic-4-d_1 acid was used as starting carboxylic acid. Purity: 70 %; Yield: 80 %. ^{1}H-NMR ($CDCl_3$, 400 MHz, 298 K) δ, ppm**:** 11.38 (s, 1H), 2.44 (t, J = 7.5 Hz, 2H), 1.71 (m, 2H), 1.30 (m, 15H), 0.91 (t, 5H)

Pernonanoic-^{18}O acid (^{18}O-d): Nonanoic-^{18}O acid (see section 8.4 for its preparation) was used as starting carboxylic acid. Purity: 86 %; Yield: 50 %. ^{1}H-NMR ($CDCl_3$, 400 MHz, 298 K) δ, ppm: 11.35 (s, 1H), 2.42 (t, *J* = 8 Hz, 2H), 1.74-1.62 (m, 2H), 1.37-1.22 (m, 12H), 0.91 (t, 3H). As nonanoic-^{18}O acid and non-labelled H_2O_2 are used for the preparation of this peracid, the ^{18}O isotope is exclusively located in the carbonyl oxygen atom of **^{18}O-d**. According to ESI-MS analysis, the prepared **^{18}O-d** contains 23 % ^{18}O (Figure S8).

(*S*)-4-methylperhexanoic acid (g): (*S*)-4-methylhexanoic acid was used as starting carboxylic acid. Purity: 100 %; Yield: 63 %.^{1}H-NMR ($CDCl_3$, 400 MHz, 298 K) δ, ppm: 2.37 (m, 2H), 1.70 (m, 1H), 1.29 (m, 4H), 0.90 (m, 6H).

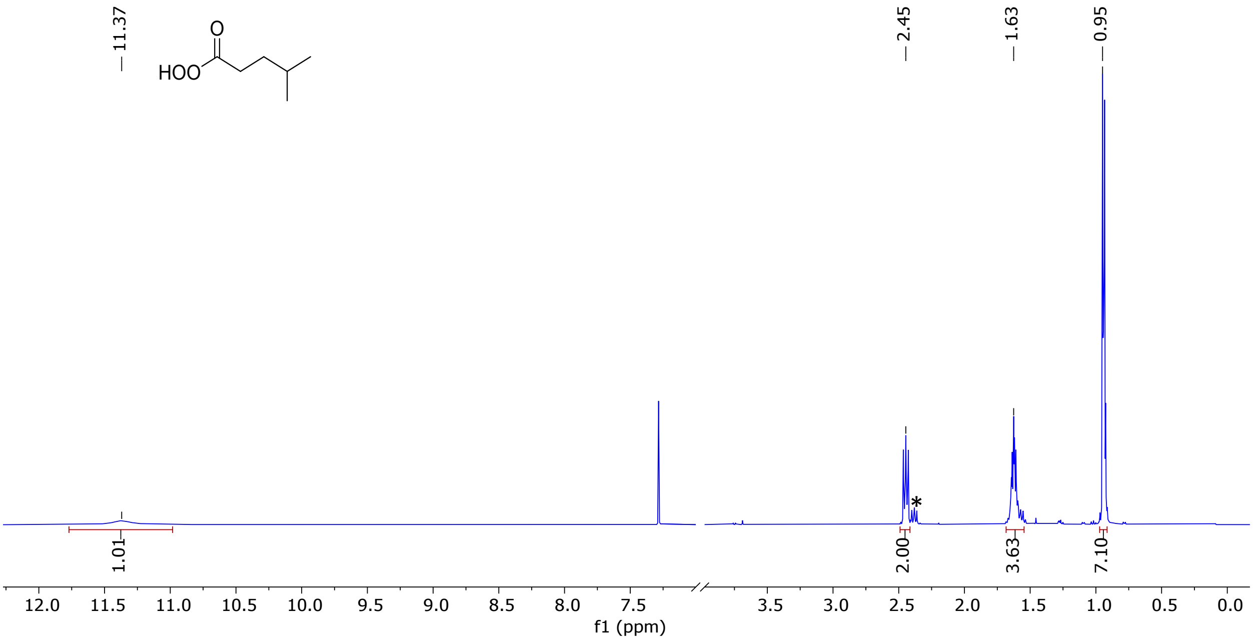

Figure S1. ^{1}H-NMR spectrum of 4-methylpervaleric acid (**c**) in $CDCl_3$ at 298 K, purity: 73 %. **Peaks corresponding to the acid.*

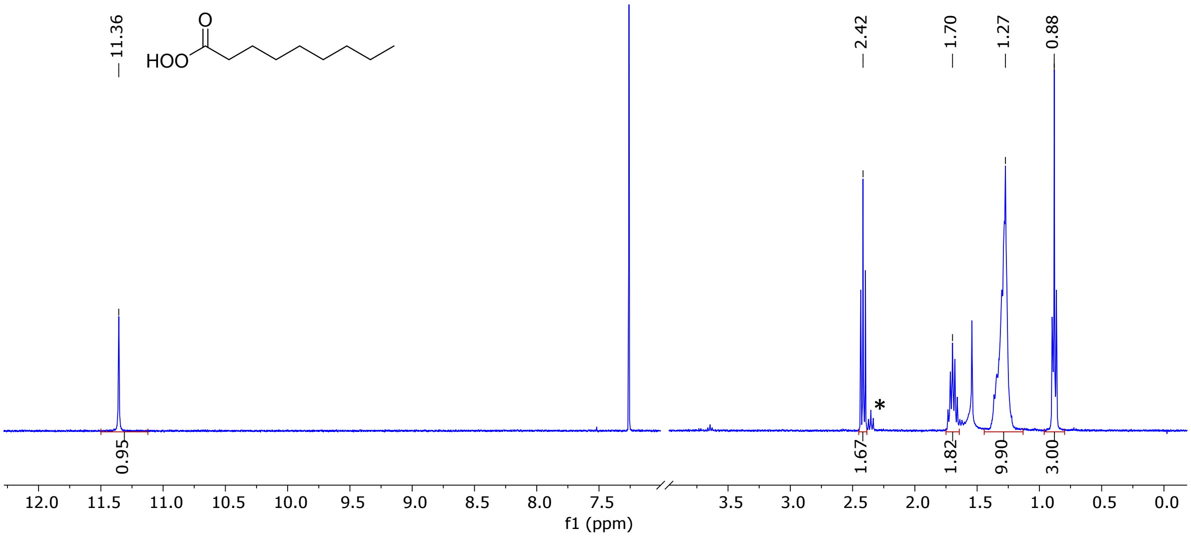

Figure S2. ^{1}H-NMR spectrum of pernonanoic acid (**d**) in $CDCl_3$ at 298 K, purity: 85%. **Peaks corresponding to the acid*.

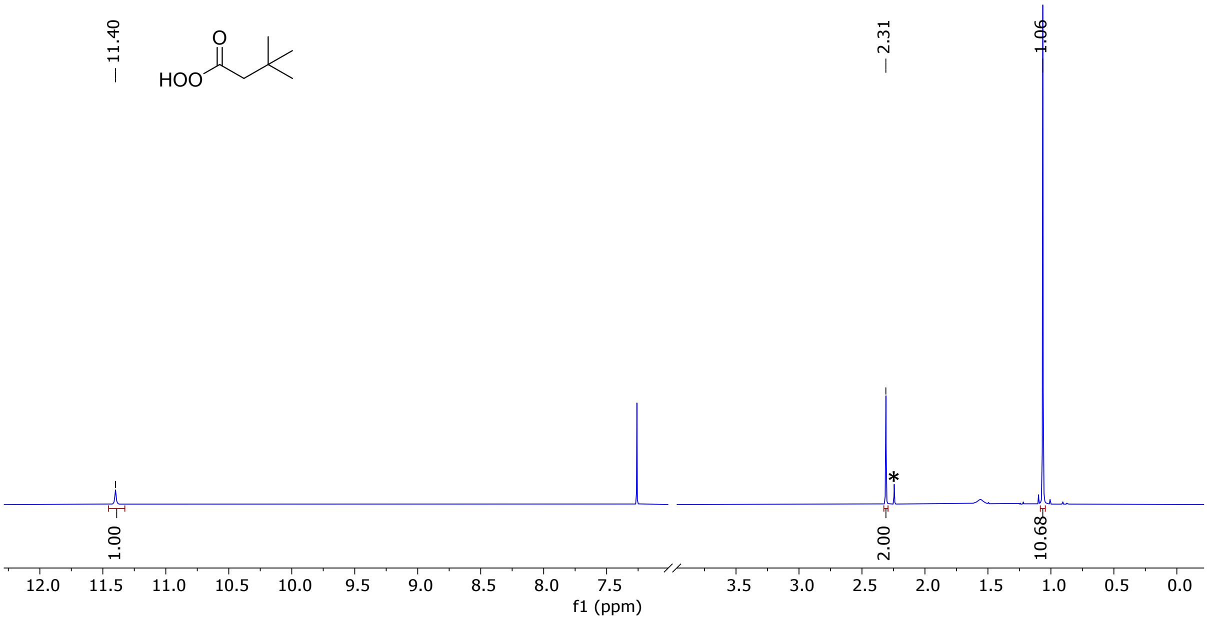

Figure S3. ^{1}H-NMR spectrum of *tert*-butyl peracetic acid (**e**) in $CDCl_3$ at 298 K, purity: 90 %. **Peaks corresponding to the acid.*

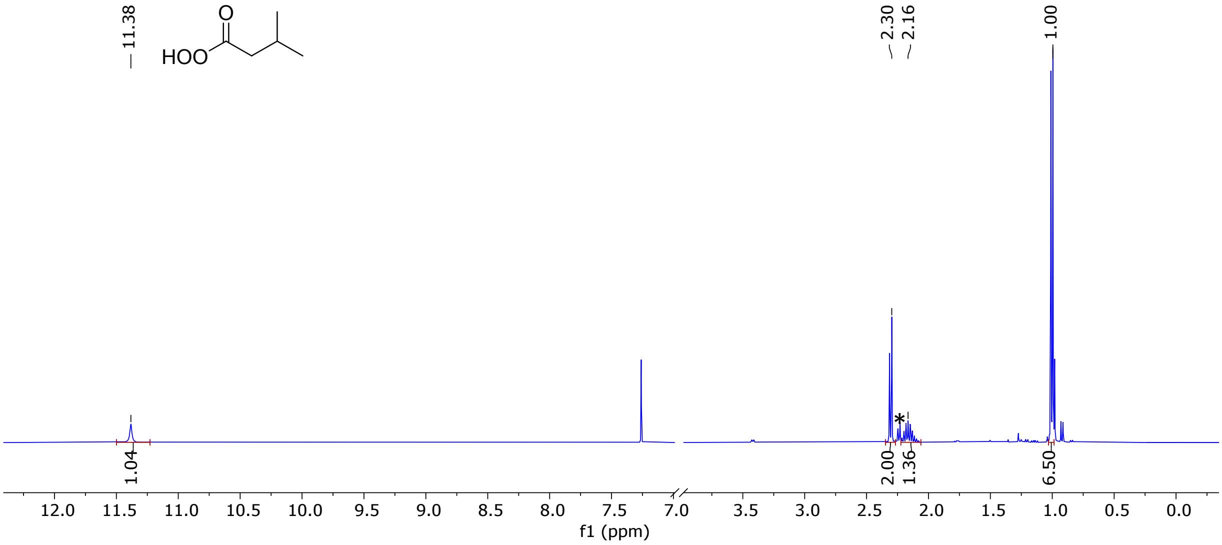

Figure S4. ^{1}H-NMR spectrum of isopervaleric acid (**f**) in $CDCl_3$ at 298 K, purity: 83 %. **Peaks corresponding to the acid.*

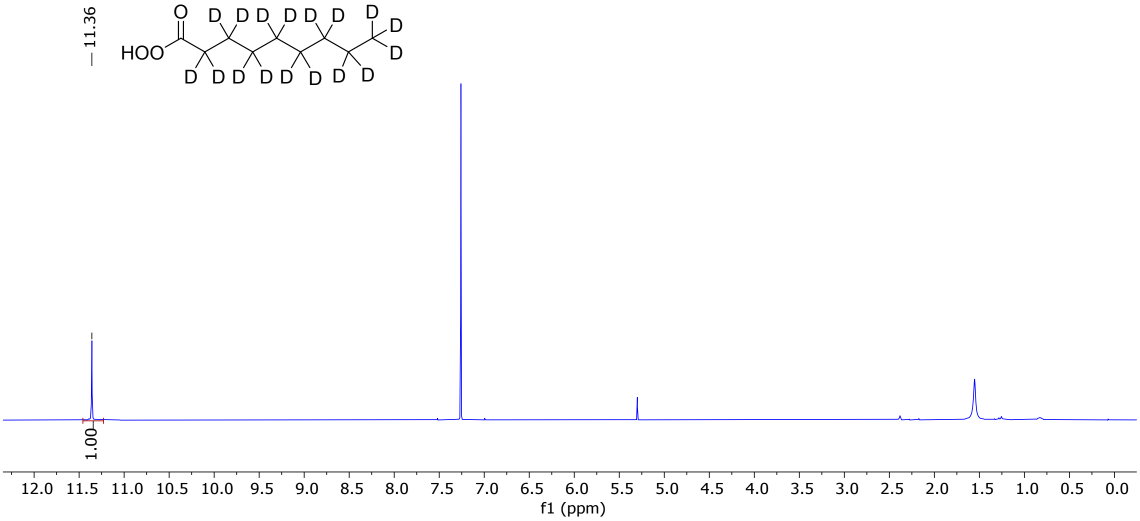

Figure S5. ^{1}H-NMR spectrum of pernonanoic-d_{17} acid (***d*$_{17}$-d**) in $CDCl_3$ at 298 K, purity: 100 %.

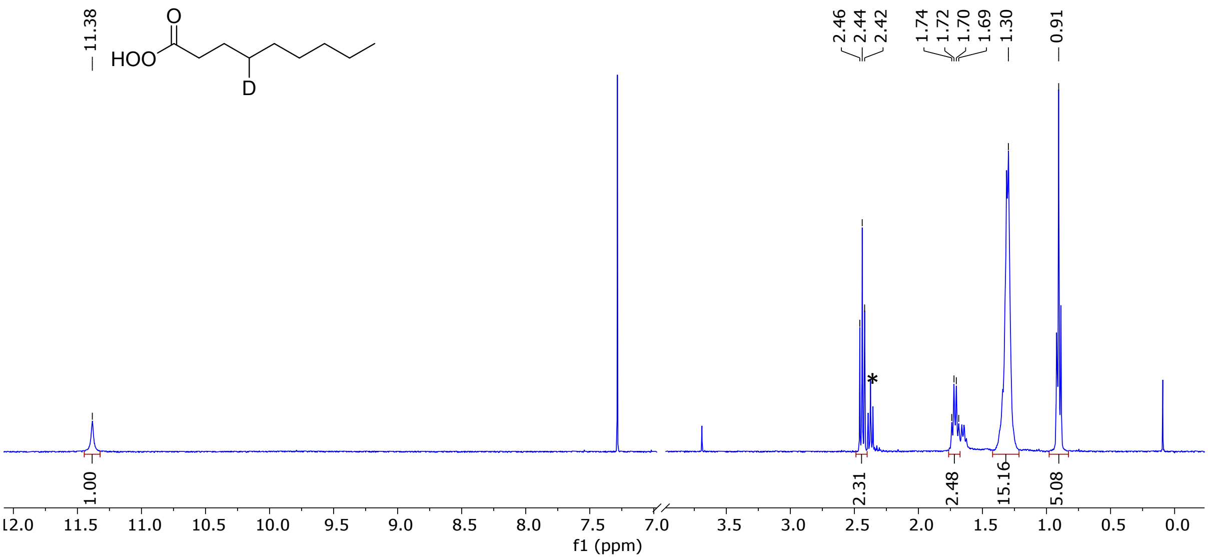

Figure S6. ^{1}H-NMR spectrum of pernonanoic-4-d_1 acid (***d*₁-d**) in $CDCl_3$ at 298 K, purity: 70 %. **Peaks corresponding to the acid*

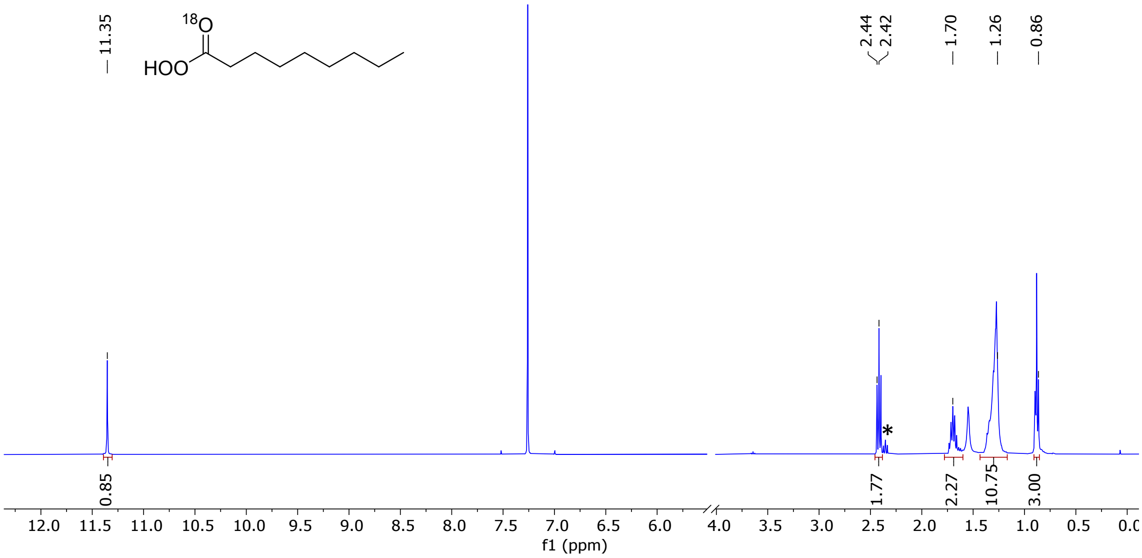

Figure S7. ^{1}H-NMR spectrum of pernonanoic-^{18}O acid (**^{18}O-d**) in $CDCl_3$ at 298 K, purity: 86 %. **Peaks corresponding to the acid*

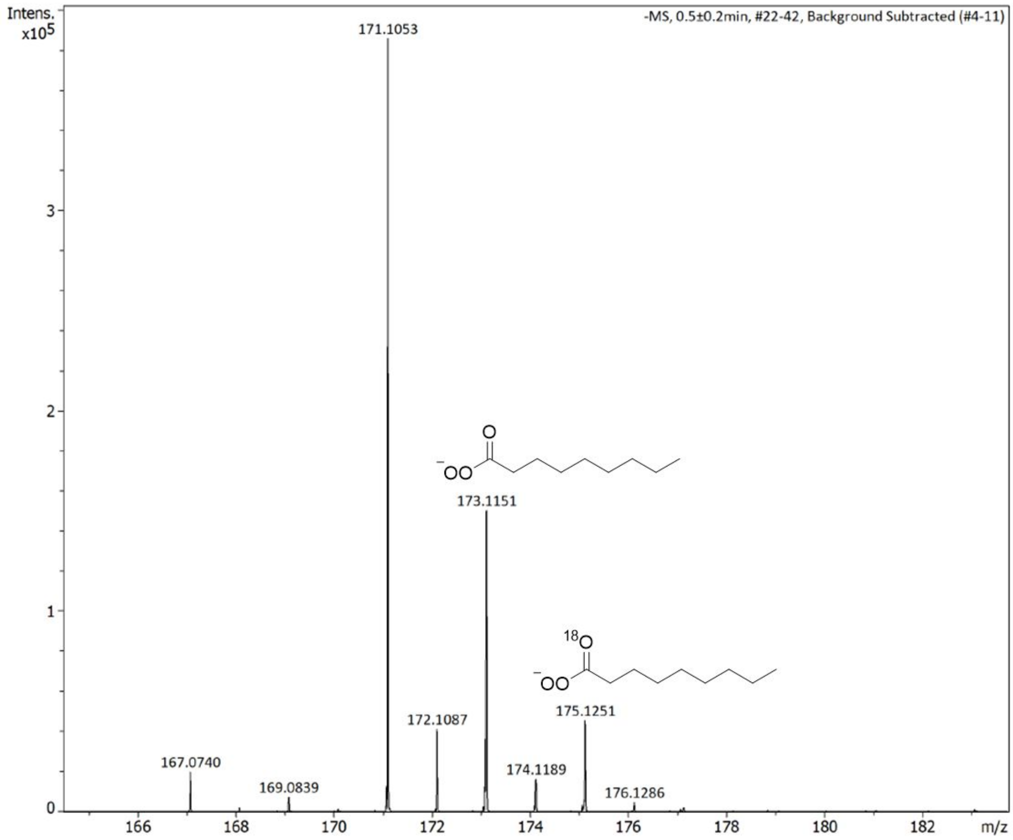

Figure S8. ESI-MS spectrum of pernonanoic-^{18}O acid (**^{18}O-d**). The calculated ^{18}O-content according to the isotopic pattern is 23 %.

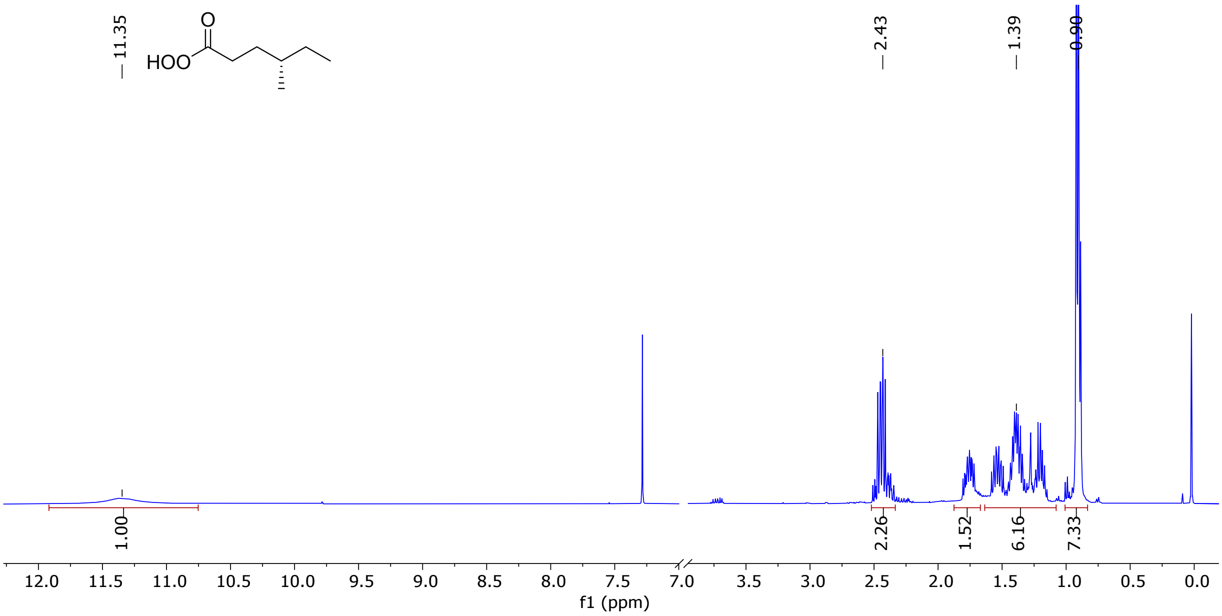

Figure S9. ^{1}H-NMR spectrum of (*S*)-4-methylperhexanoic acid (**g**) in $CDCl_3$ at 298 K, purity: 100 %.

3. Generation of the $[Fe^{V}(O)(OC(O)R)(PyNMe_3)]^{2+}$, reaction with external substrates and product analyses

Inside the glovebox, 500 μL (2 μmols) of a 4 mM solution of $[Fe^{II}(PyNMe_3)(NCCH_3)_2]^{2+}$ (**1**) (obtained by dissolving $[Fe^{II}(CF_3SO_3)_2(PyNMe_3)]$ in dry acetonitrile) and 1.5 mL of dry acetonitrile were placed in a UV-vis cuvette. Afterwards, the quartz cuvette was capped with a septum and taken out from the glovebox to subsequently be placed in the Unisoku cryostat of the UV-vis spectrophotometer and cooled down to 233 K. After reaching thermal equilibrium, 50-100 μL (8 - 16 μmol) of a 0.16 M solution of the desired peracid in dry acetonitrile were added to generate **2c-g**. The formation and decay of these species were followed by UV-vis spectroscopy, monitoring their characteristic absorption band at λ_{max} ~ 500 nm (the exact position of this band slightly changes depending on the nature of the peracid used). These species are not stable at 233 K, and they decompose at this temperature (the decay rate depends on the peracid used).

For the competition experiments with external substrates, once the absorption band of **2c** or **2d** achieved its maximal intensity, 100 equiv of cyclohexane (200 μmol) or 20 equiv of 1-octene (40 μmol) dissolved in 100-200 μL acetonitrile were added at once. The decay of the absorption band of **2c** or **2d** was significantly accelerated when any of these two substrates was added.

Analysis of the final organic products. Once **2** had fully decayed, 40% $NaHSO_3$ aqueous solution (100 μL) was added to the cuvette to quench the reaction and destroy the unreacted peracid. Then, biphenyl (100 μL from a 10 mM solution in acetonitrile) was added to the solution as an internal standard and the iron complex was removed by passing the solution through a short plug of silica and $MgSO_4$, which was subsequently rinsed with ethyl acetate (1 mL). The resulting mixture was analysed by GC and GC-MS in order to detect and quantify γ-lactones, cyclohexanol and 1,2-epoxyoctane, depending on the specific experiment. Product yields were calculated with respect to the internal standard using the response factor obtained by a calibration curve.

Table S1. Amounts of γ-lactones obtained after the self-decay of **2c**-**2f** under different reaction conditions.

compound	equiv peracid[a]	temperature	solvent	amount of lactone[b]	
**2c**	4 equiv	-40 ºC	CH_3CN	2 TON	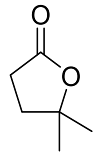
	8 equiv	-40 ºC	CH_3CN	5 TON	
**2d**	4 equiv	-40 ºC	CH_3CN	1 TON	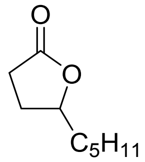
	8 equiv	-40 ºC	CH_3CN	2 TON	
**2e**	4 equiv	-40 ºC	CH_3CN	n.d.	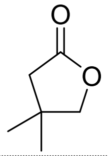
	10 equiv	-40 ºC	CH_3CN	n.d.	
	10 equiv	25 ºC	CH_3CN	0.3 TON	
	10 equiv	-20 ºC	TFE	1 TON	
**2f**	4 equiv	-40 ºC	CH_3CN	n.d.	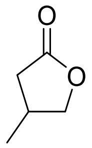
	10 equiv	-40 ºC	CH_3CN	n.d.	
	10 equiv	25 ºC	CH_3CN	0.2 TON	
	10 equiv	-20 ºC	TFE	0.6 TON	

[a]Equivalents of peracid used for the generation of **2c**-**2f** with respect to **1**.
[b]The amount of lactone produced after the self-decay of **2c**-**2f** is calculated as the mmols of lactone divided by the mmols of **1** used for the generation of **2c**-**2f**.

3.1. Relevant GC-FID and GC-MS chromatograms

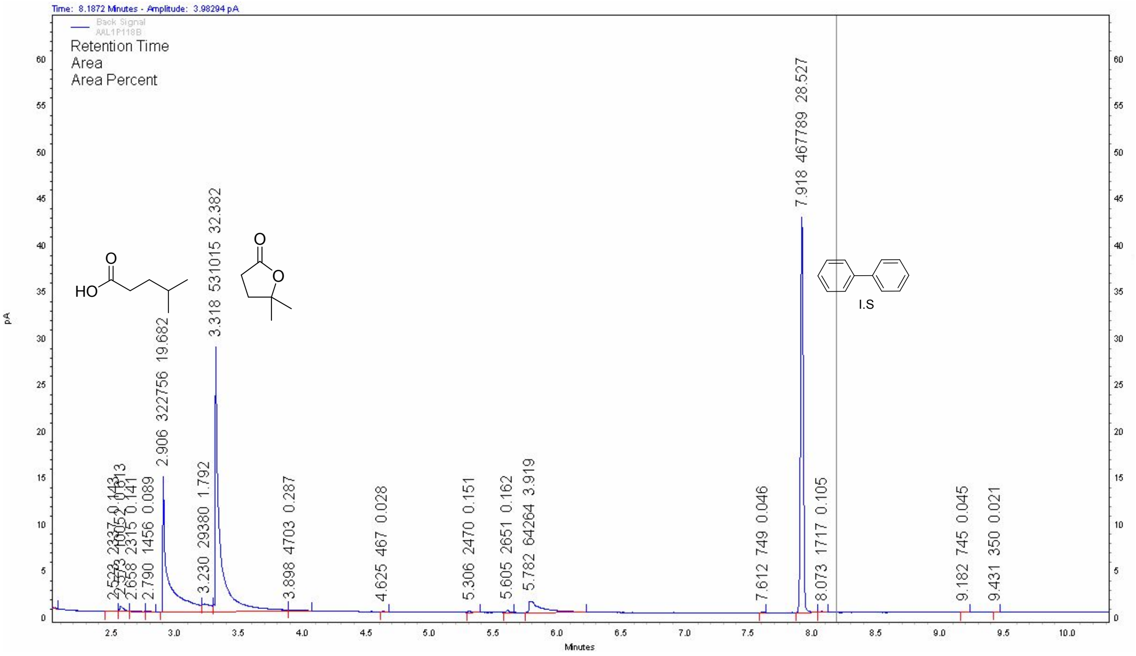

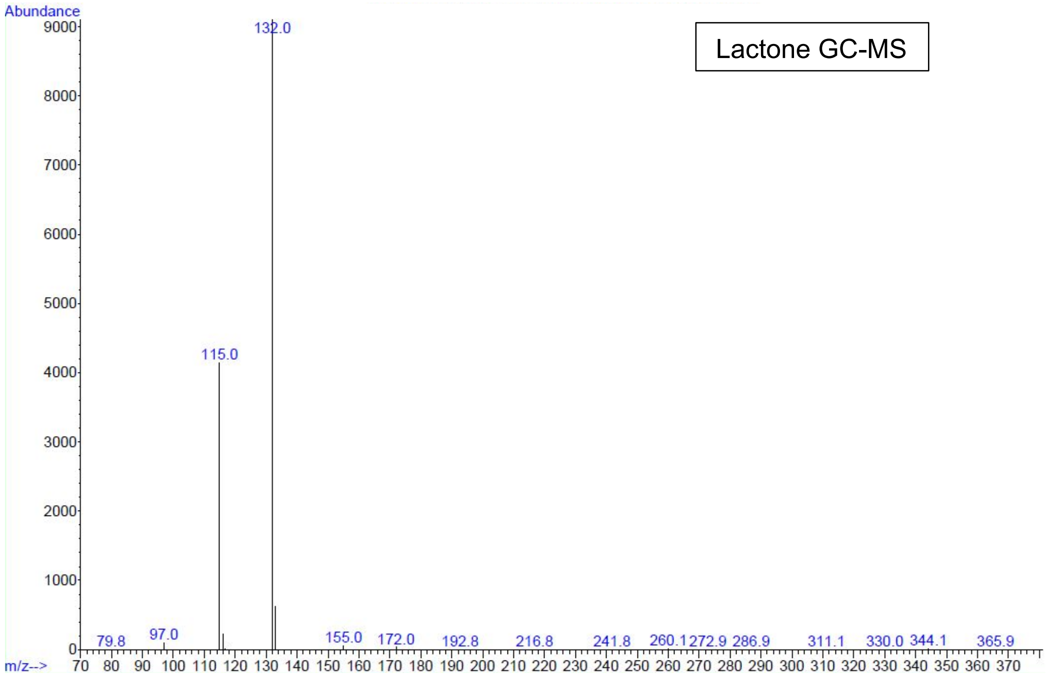

Figure S10. Top: GC-FID corresponding to the products derived from the decomposition of **2c**. Bottom: GC-MS spectrum of the lactone product.

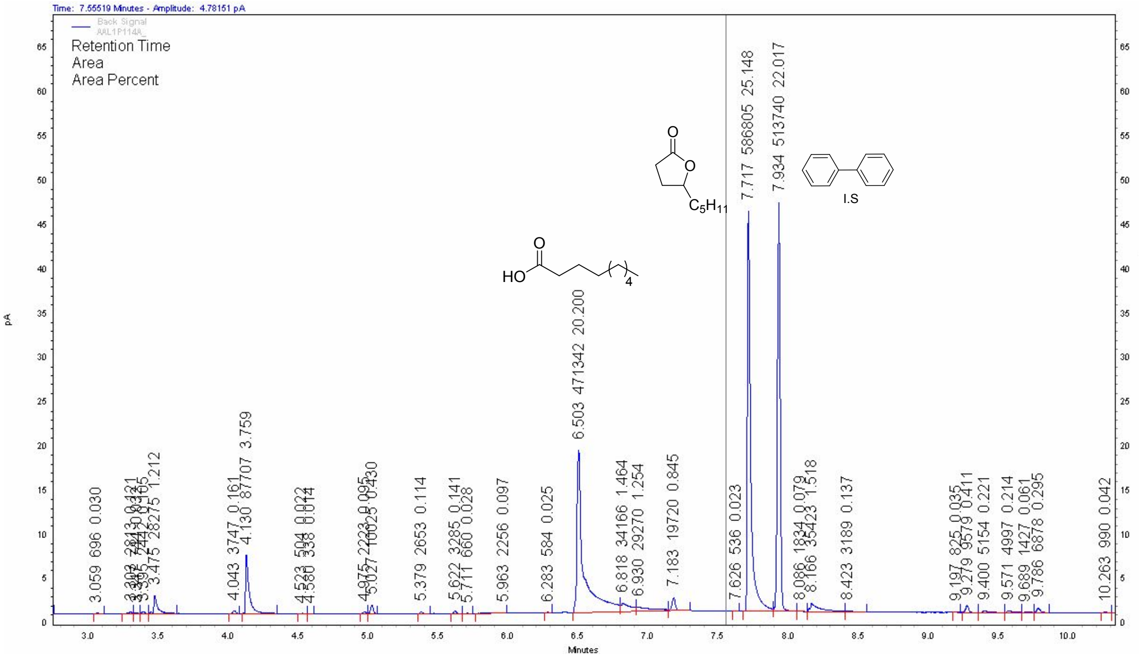

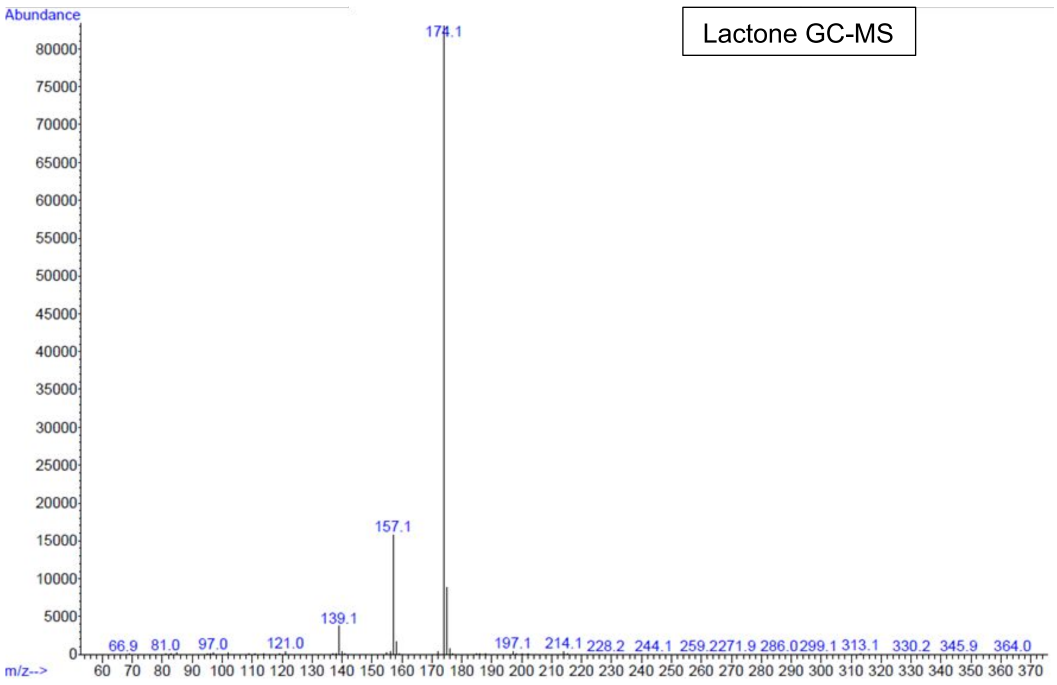

Figure S11. Top: GC-FID corresponding to the products derived from the decomposition of **2d**. Bottom: GC-MS spectrum of the lactone product.

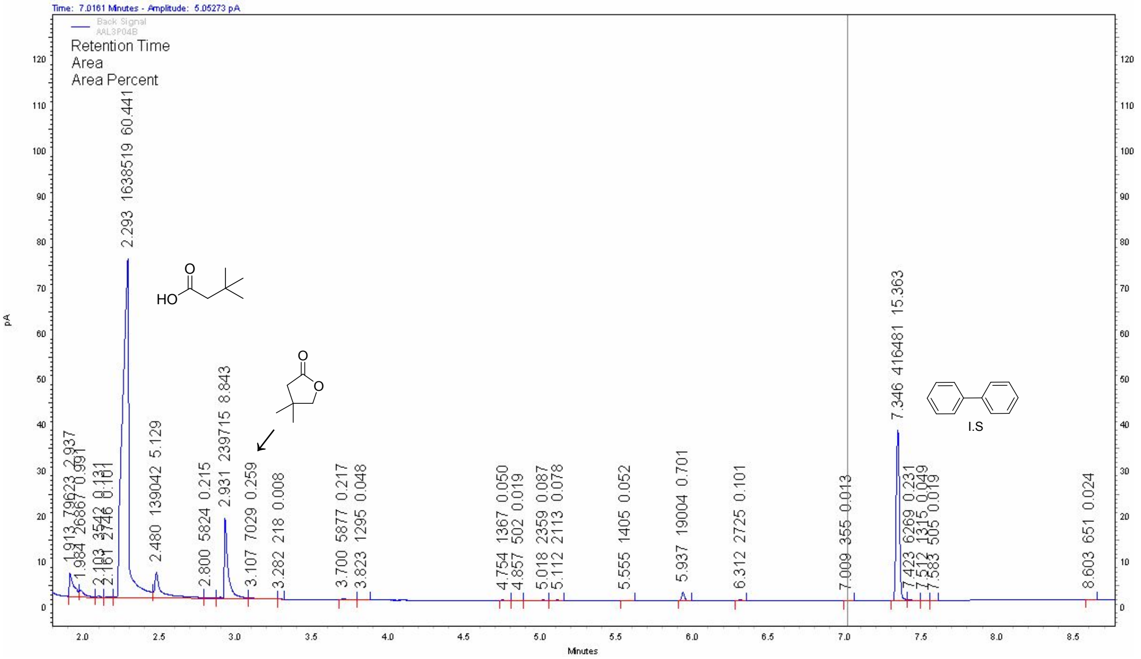

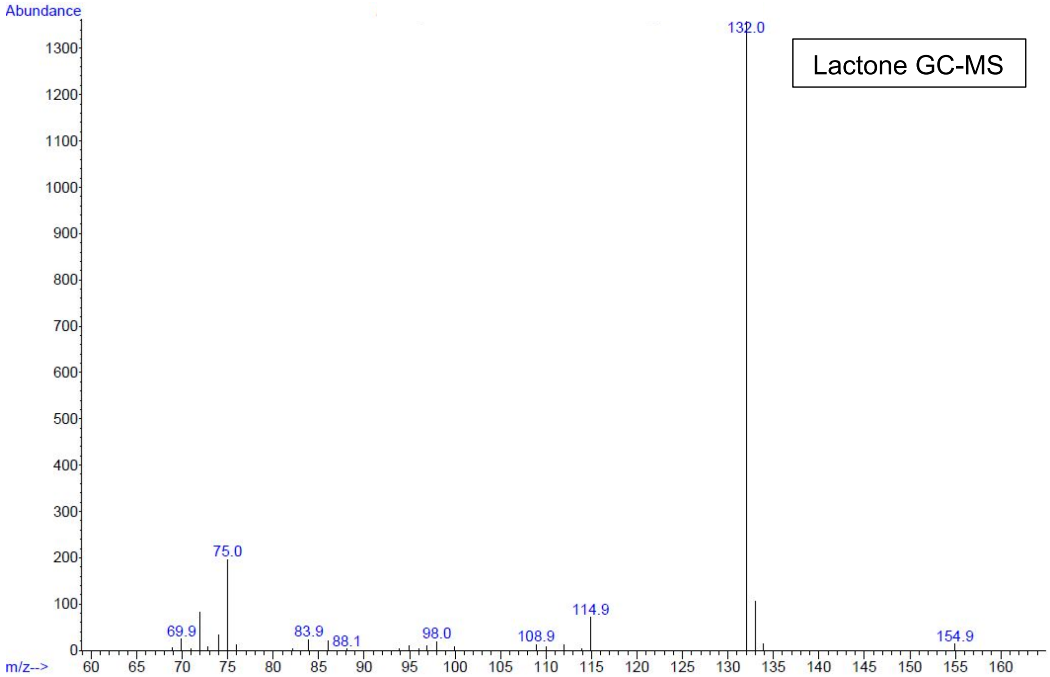

Figure S12. Top: GC-FID corresponding to the products derived from the decomposition of **2e** generated using TFE. Bottom: GC-MS spectrum of the lactone product.

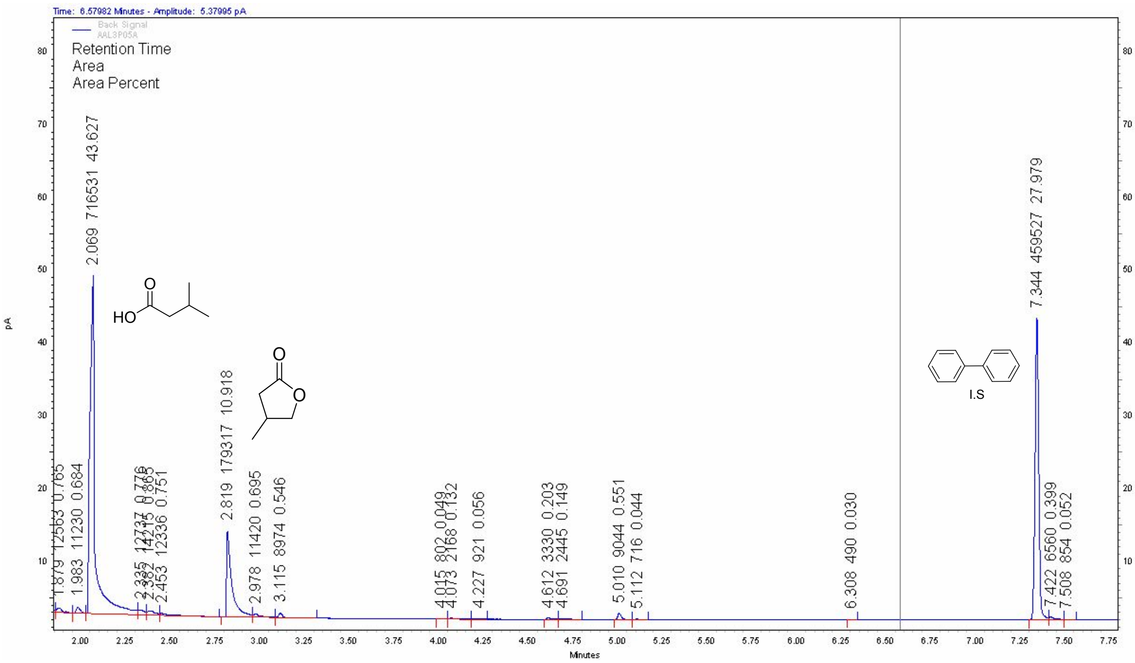

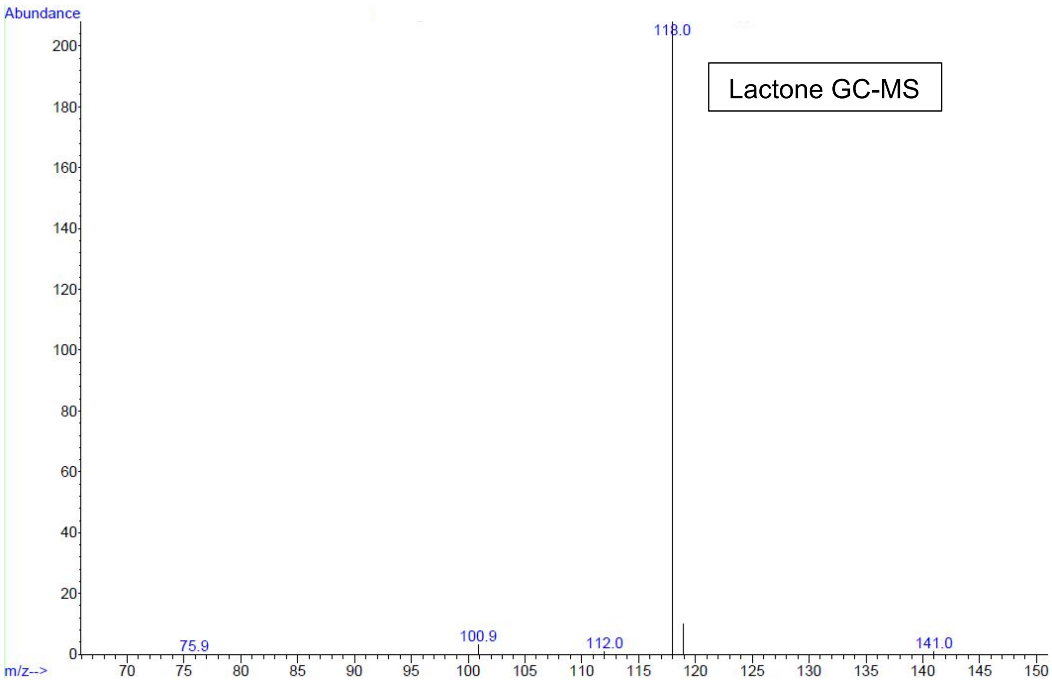

Figure S13. Top: GC-FID corresponding to the products derived from the decomposition of **2f** generated using TFE. Bottom: GC-MS spectrum of the lactone product.

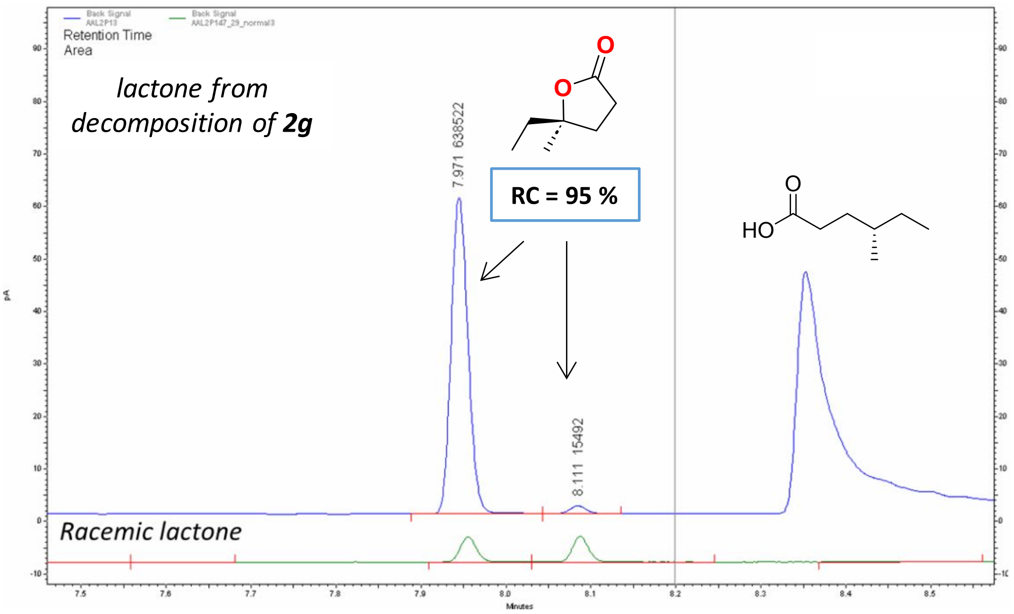

Figure S14. Chiral GC-FID corresponding to the products derived from the decomposition of **2g** (top) and the corresponding racemic lactone (bottom).

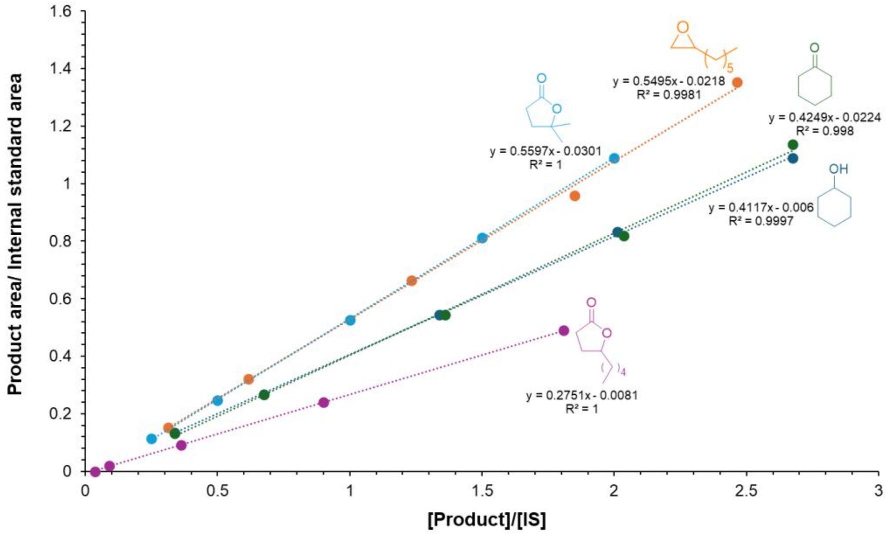

Figure S15. GC-FID calibration curves for cyclohexanol, cyclohexanone, 1,2-epoxyoctane, γ-nonalactone (derived from **2d**) and 5,5-dimethyl-dihydro-furan-2-one (derived from **2c**). For the GC quantification of γ-lactones derived from **2e** and **2f,** the response factor of 5,5-dimethyl-dihydro-furan-2-one was used.

3.2. Generation of 2e and 2f at -60 ºC

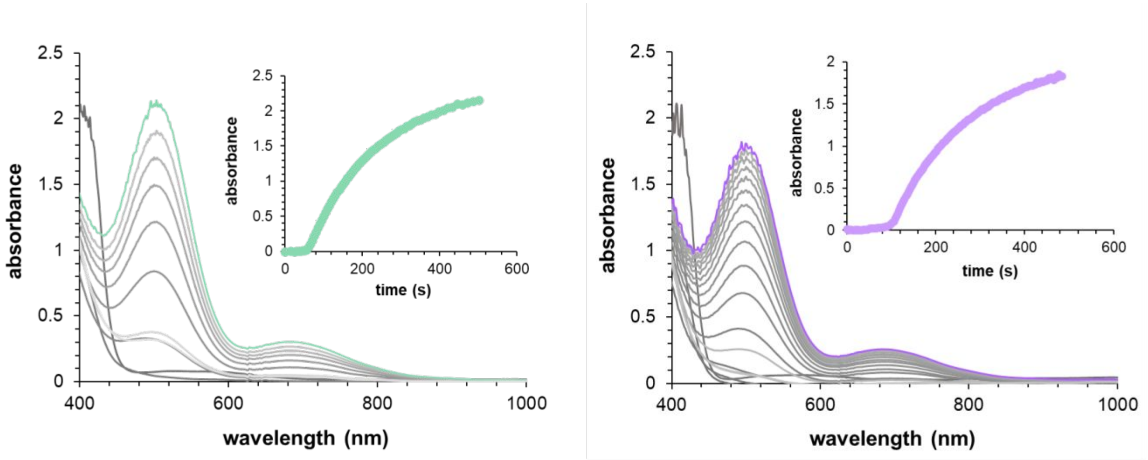

Figure S16. Left: UV-Vis spectra corresponding to the reaction of **1** (1 mM) with *tert*-butyl peracetic acid (**e**) to generate **2e** at -60 ºC in an acetone:acetonitrile 3:1 solvent mixture. Inset: Kinetic trace at 503 nm. Right: UV-Vis spectra corresponding to the reaction of **1** (1 mM) with isopervaleric acid (**f**) to generate **2f** at -60 ºC in an acetone:acetonitrile 3:1 solvent mixture. Inset: Kinetic trace at 498 nm.

3.3. Generation of 2c-2f at -40 ºC

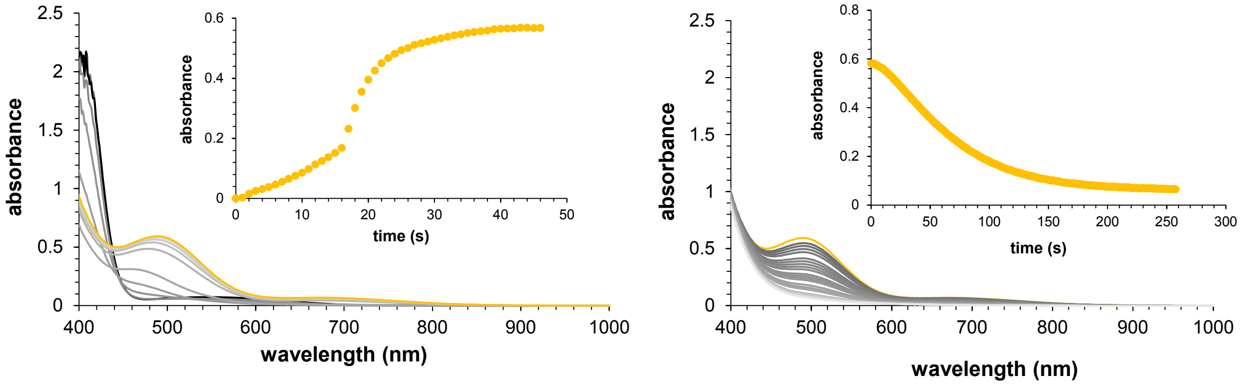

Figure S17. Left: UV-Vis spectra corresponding to the reaction of **1** (1 mM) with 4-methylpervaleric acid (**c**) to generate **2c** at -40 ºC acetonitrile. Inset: Kinetic trace at 488 nm. Right: UV-Vis spectra corresponding to the decomposition of **2c** at -40 ºC in an acetonitrile. Inset: Kinetic trace at 488 nm.

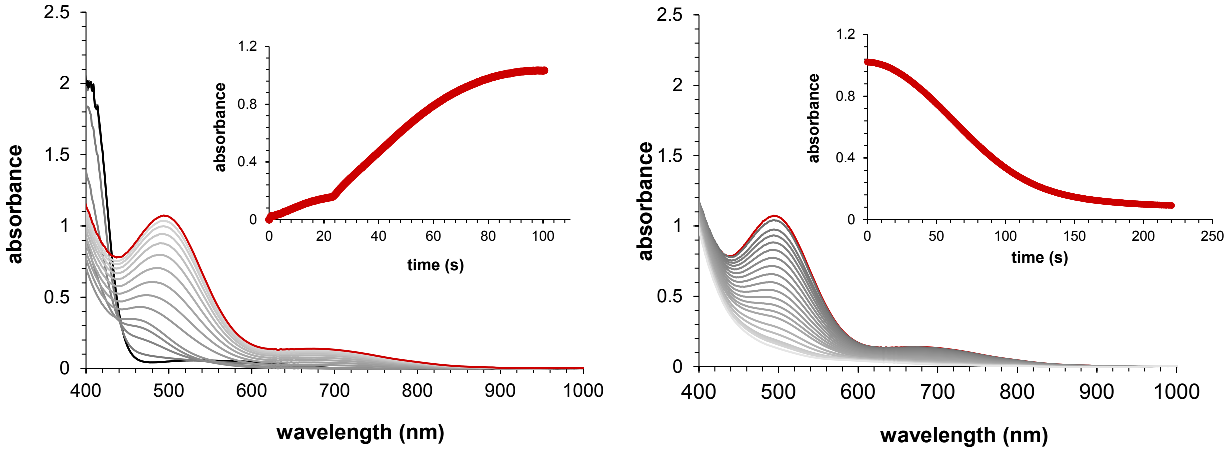

Figure S18. Left: UV-Vis spectra corresponding to the reaction of **1** (1 mM) with pernonanoic acid (**d**) to generate **2d** at -40 ºC acetonitrile. Inset: Kinetic trace at 497 nm. Right: UV-Vis spectra corresponding to the decomposition of **2d** at -40 ºC in an acetonitrile. Inset: Kinetic trace at 497 nm.

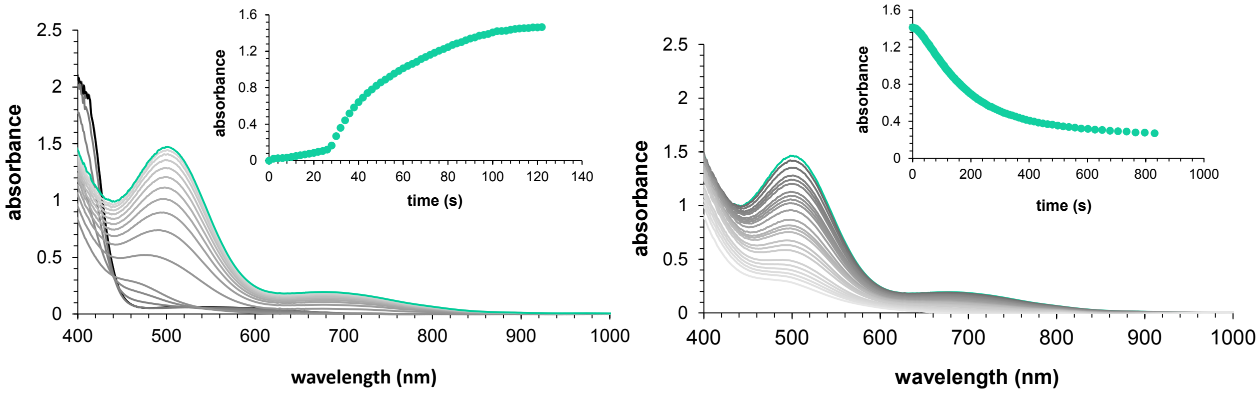

Figure S19. Left: UV-Vis spectra corresponding to the reaction of **1** (1 mM) with *tert*-butylperacetic acid (**e**) to generate **2e** at -40 ºC acetonitrile. Inset: Kinetic trace at 503 nm. Right: UV-Vis spectra corresponding to the decomposition of **2e** at -40 ºC in an acetonitrile. Inset: Kinetic trace at 503 nm.

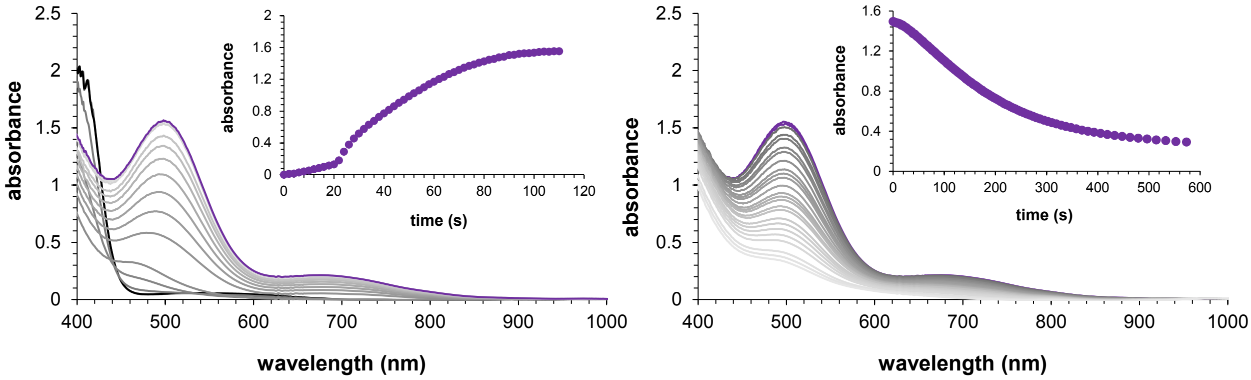

Figure S20. Left: UV-Vis spectra corresponding to the reaction of **1** (1 mM) with isopervaleric acid (**f**) to generate **2f** at -40 ºC acetonitrile. Inset: Kinetic trace at 498 nm. Right: UV-Vis spectra corresponding to the decomposition of **2f** at -40 ºC in an acetonitrile. Inset: Kinetic trace at 498 nm.

3.4. Monitoring lactone formation over time for 2d

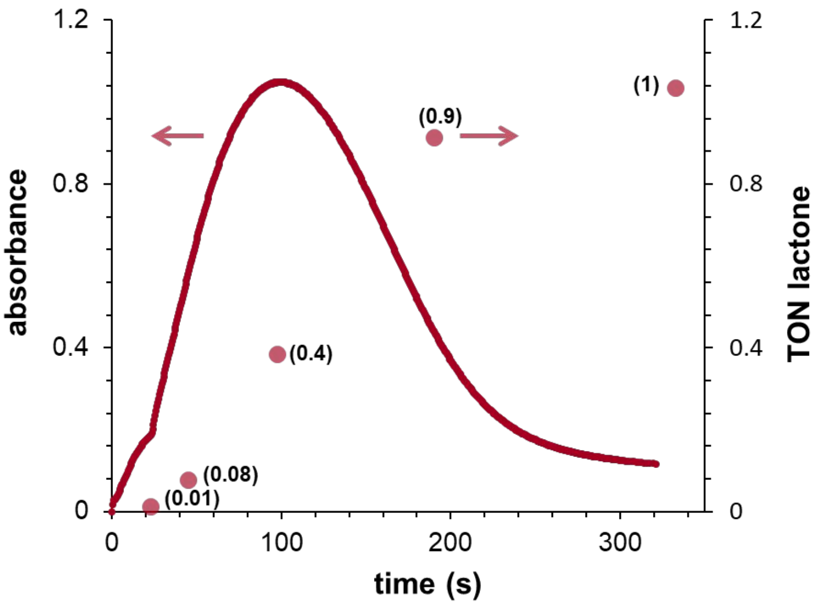

Figure S21. Kinetic trace at 497 nm corresponding to the formation and decay of **2d** obtained by reaction of **1** (1 mM) with 4 equiv of **d** in acetonitrile at -40 ºC (solid line), along with the amount of γ-lactone detected at different reaction times during the formation/decay of **2d** (dots).

4. Cryospray-MS experiments (CSI-MS)

In a UV-vis cell, species **2c-2f** were generated following the methodology described in section 3 but changing the acetonitrile solvent by an acetone:acetonitrile 3:1 mixture and setting the temperature at -60 ºC instead of -40 ºC. The mixture was injected into the MS spectrometer and the spectrum was recorded (Figure S22-S29).

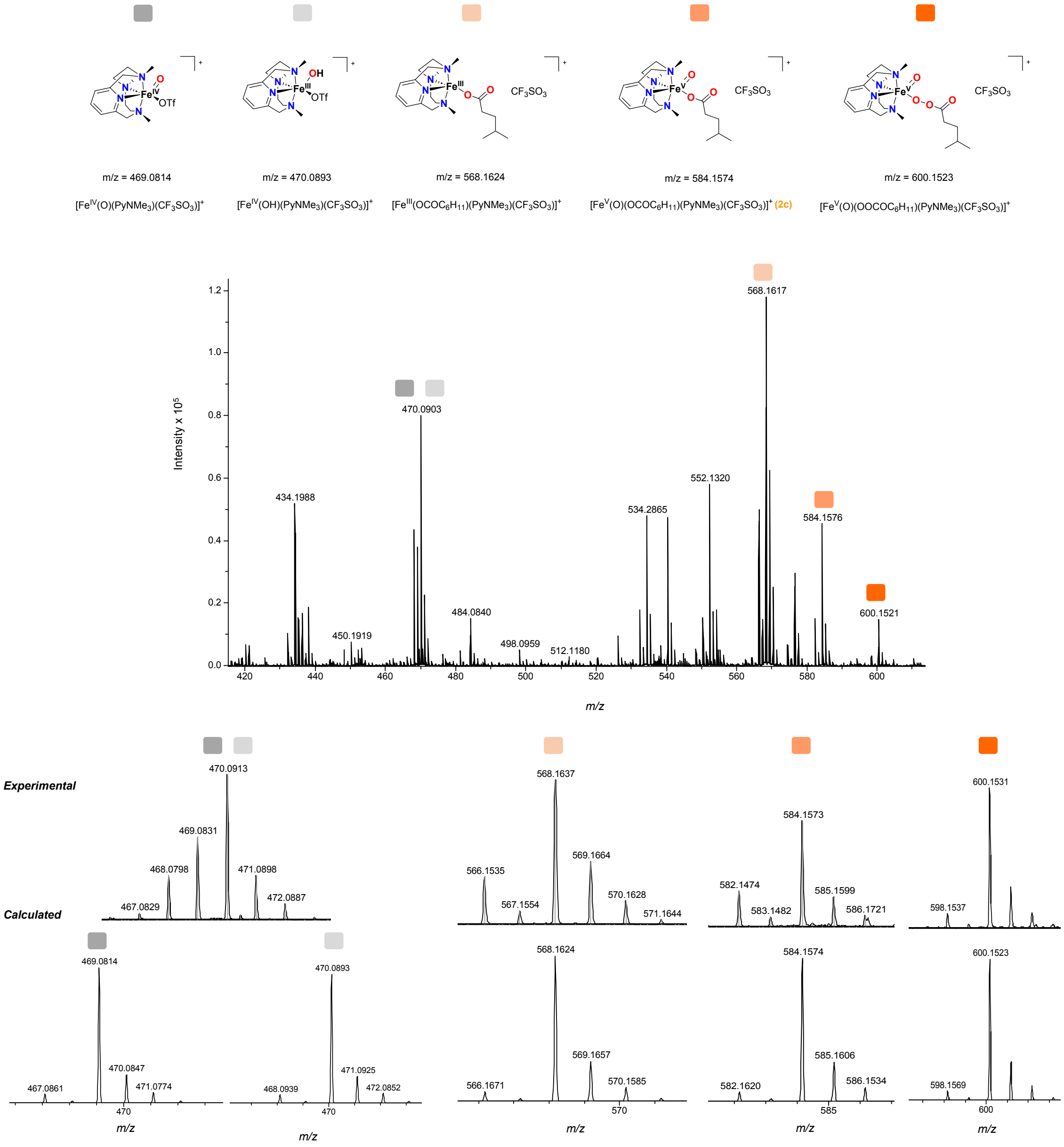

Figure S22. CSI-MS at -60 ºC in the 420 to 600 m/z region of **2c** generated by reaction of **1** with 5 equiv of 4-methylpervaleric acid in a mixture of acetone:acetonitrile 1:3.

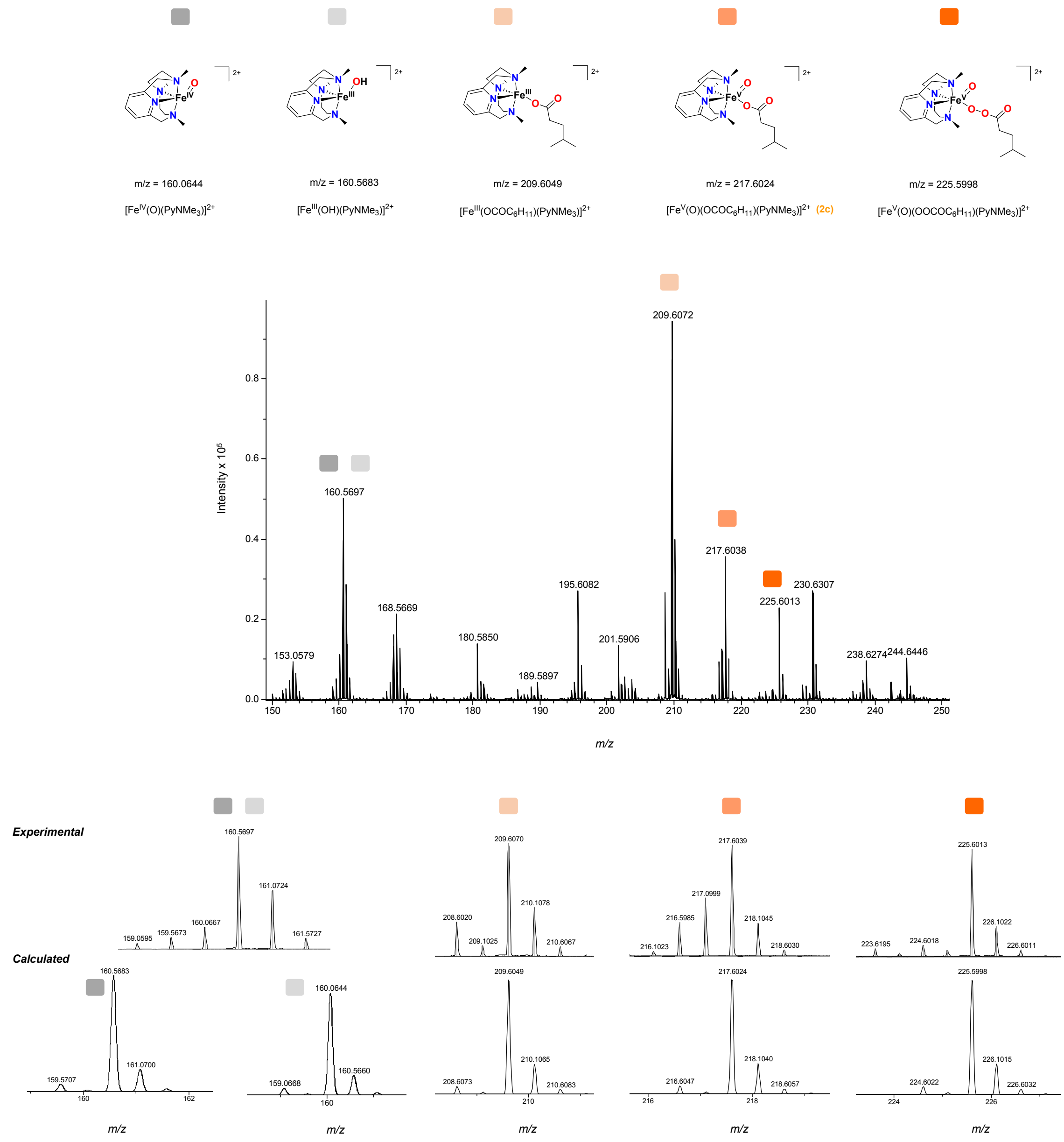

Figure S23. CSI-MS at -60 ºC in the 150 to 250 m/z region of **2c** generated by reaction of **1** with 5 equiv of 4-methylpervaleric acid in a mixture of acetone:acetonitrile 1:3.

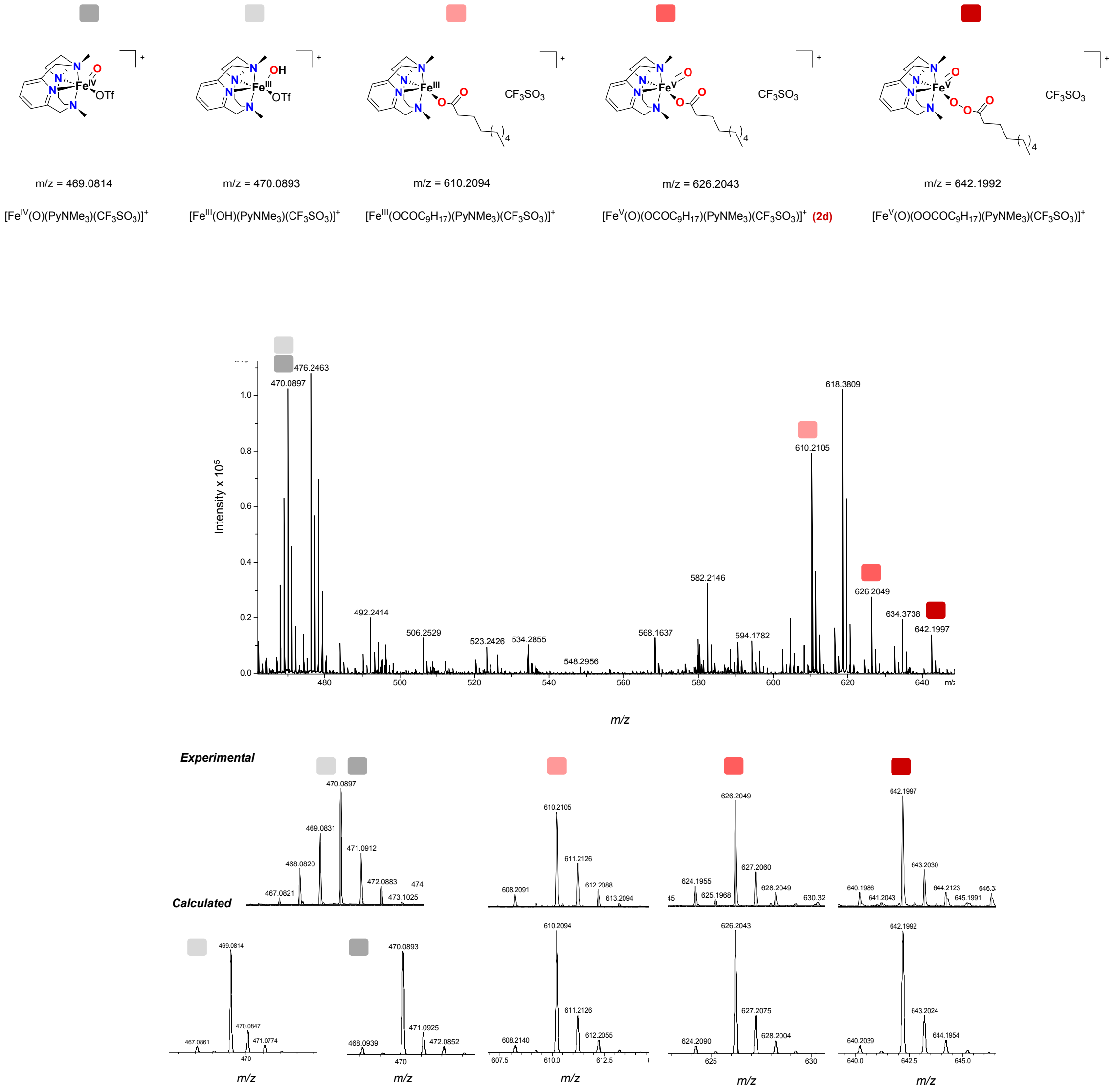

Figure S24. CSI-MS at -60 ºC in the 460 to 650 m/z region of **2d** generated by reaction of **1** with 5 equiv of pernonanoic acid in a mixture of acetone:acetonitrile 1:3.

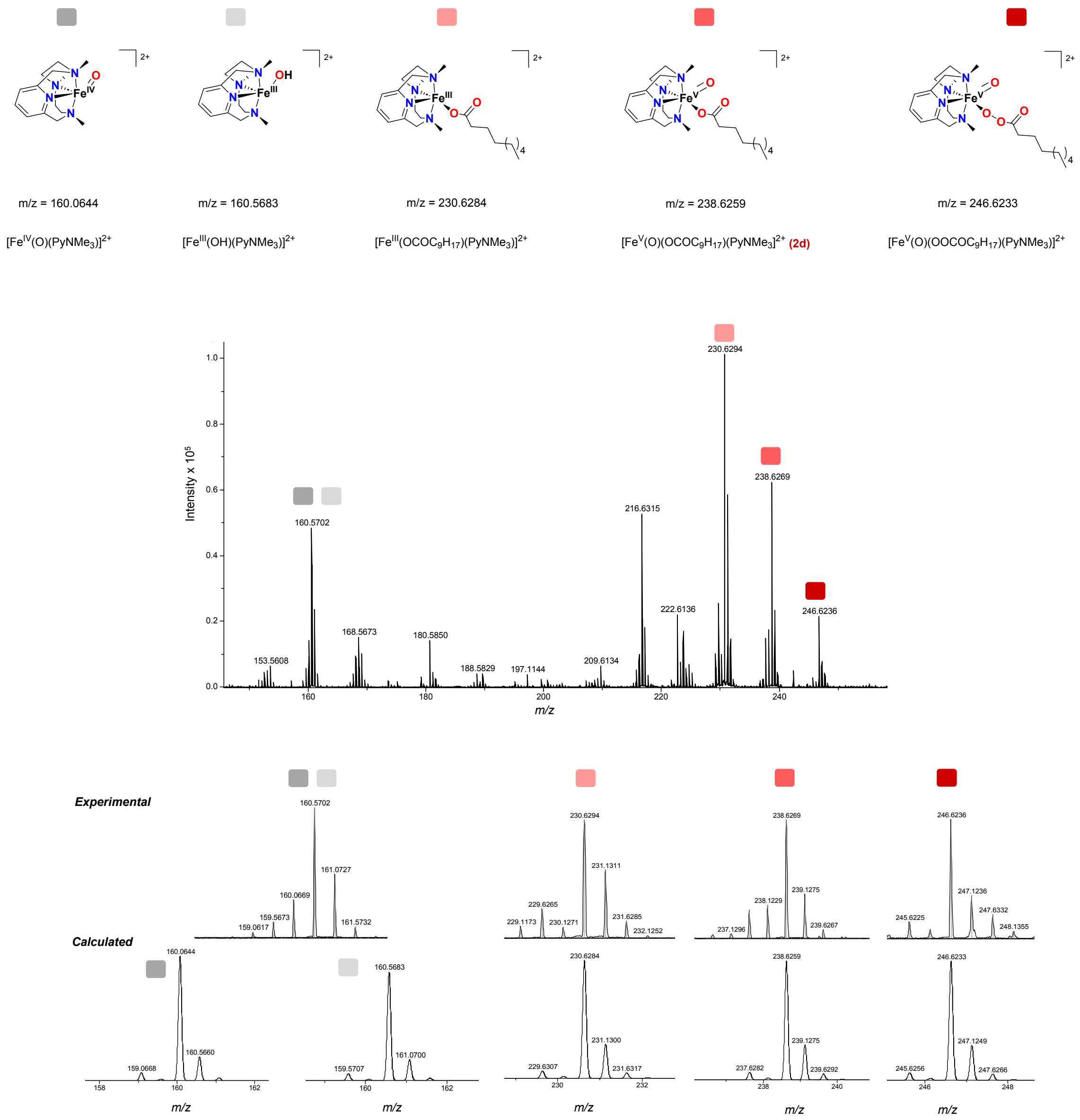

Figure S25. CSI-MS at -60 ºC in the 150 to 250 m/z region of **2d** generated by reaction of **1** with 5 equiv of pernonanoic acid in a mixture of acetone:acetonitrile 1:3.

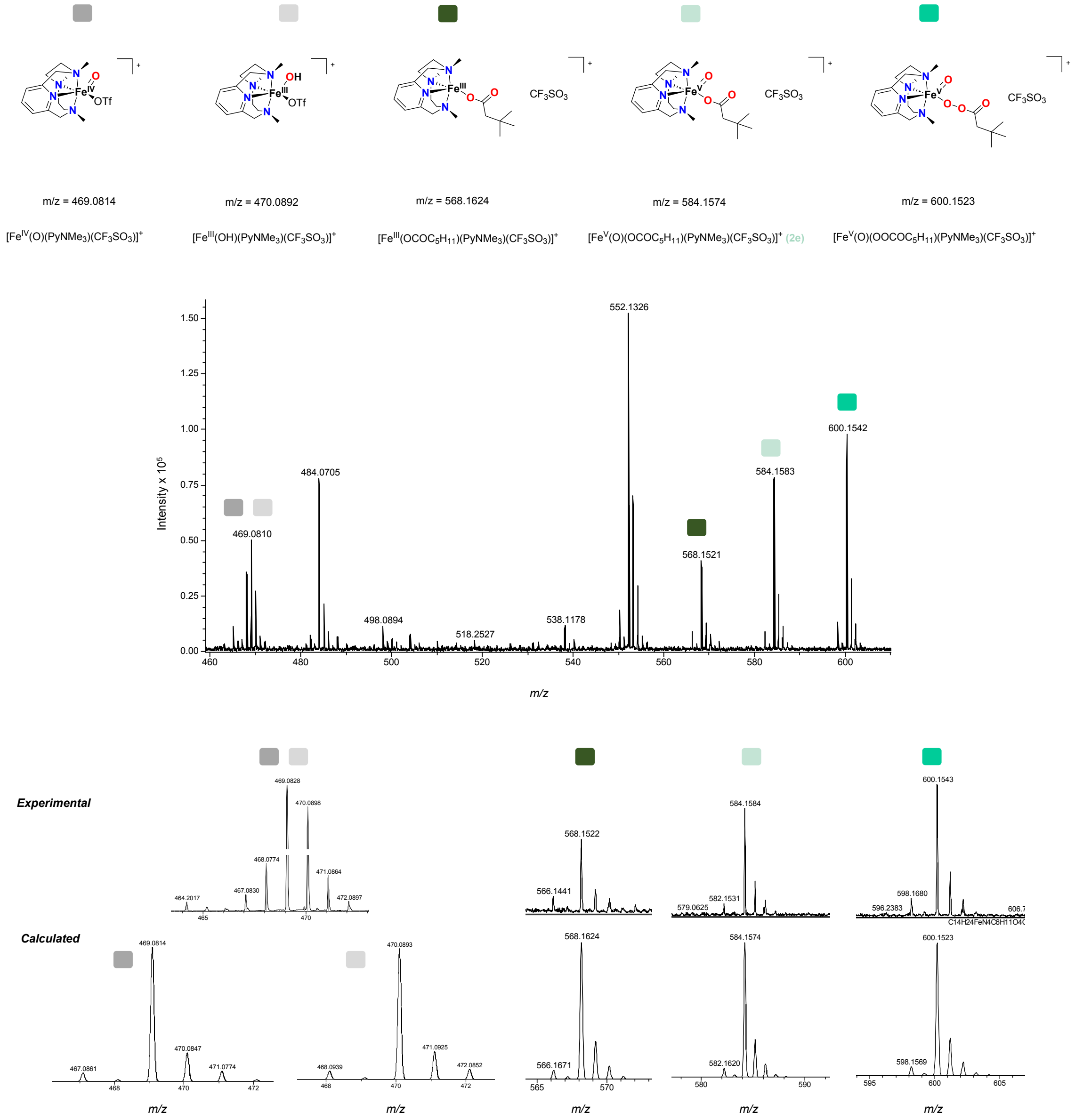

Figure S26. CSI-MS at -60 ºC in the 460 to 600 m/z region of **2e** generated by reaction of **1** with 10 equiv of *tert*-butylperacetic acid in a mixture of acetone:acetonitrile 1:3.

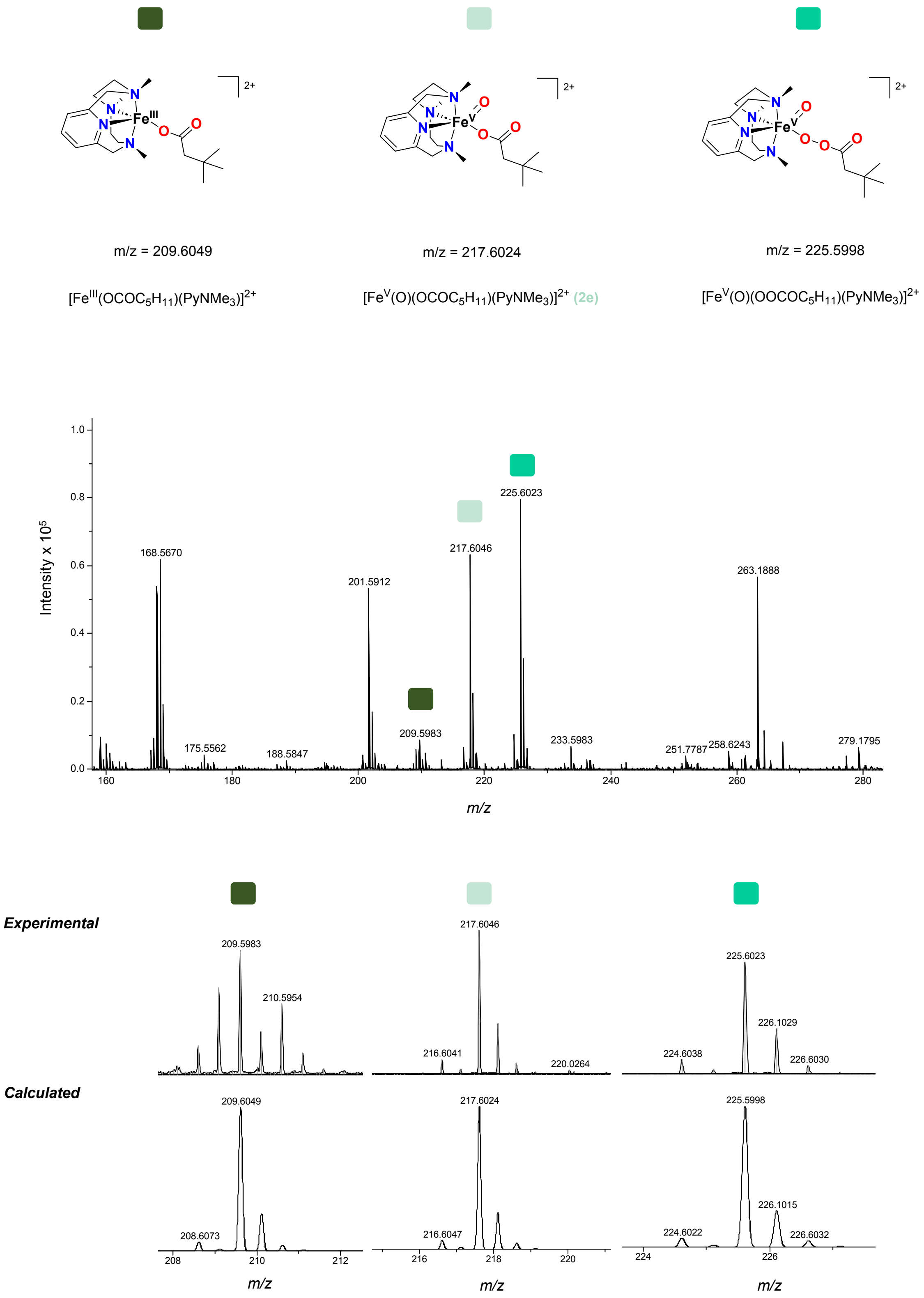

Figure S27. CSI-MS at -60 ºC in the 160 to 280 m/z region of **2e** generated by reaction of **1** with 10 equiv of *tert*-butylperacetic acid in a mixture of acetone:acetonitrile 1:3.

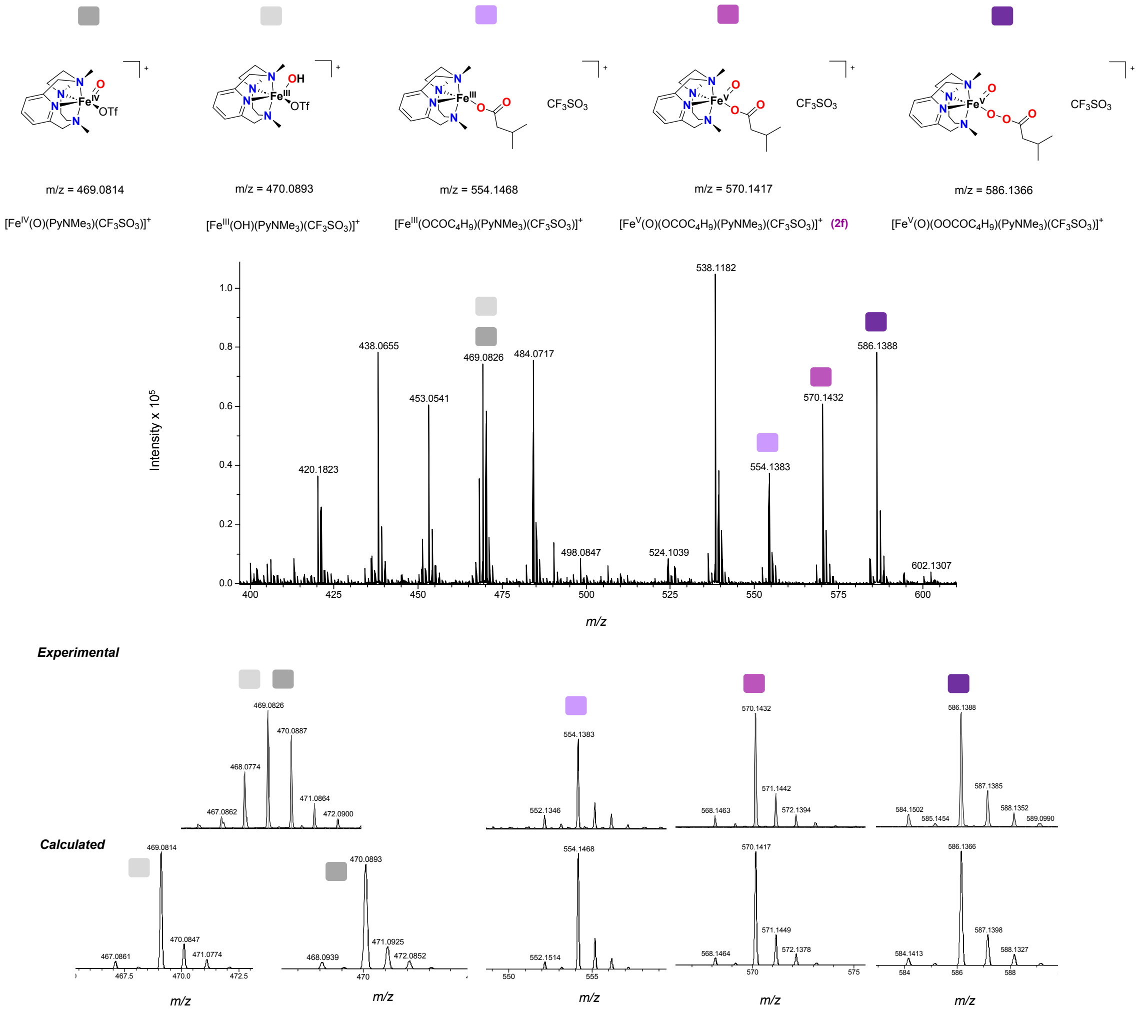

Figure S28. CSI-MS at -60 ºC in the 400 to 600 m/z region of **2f** generated by reaction of **1** with 10 equiv of isopervaleric acid in a mixture of acetone:acetonitrile 1:3.

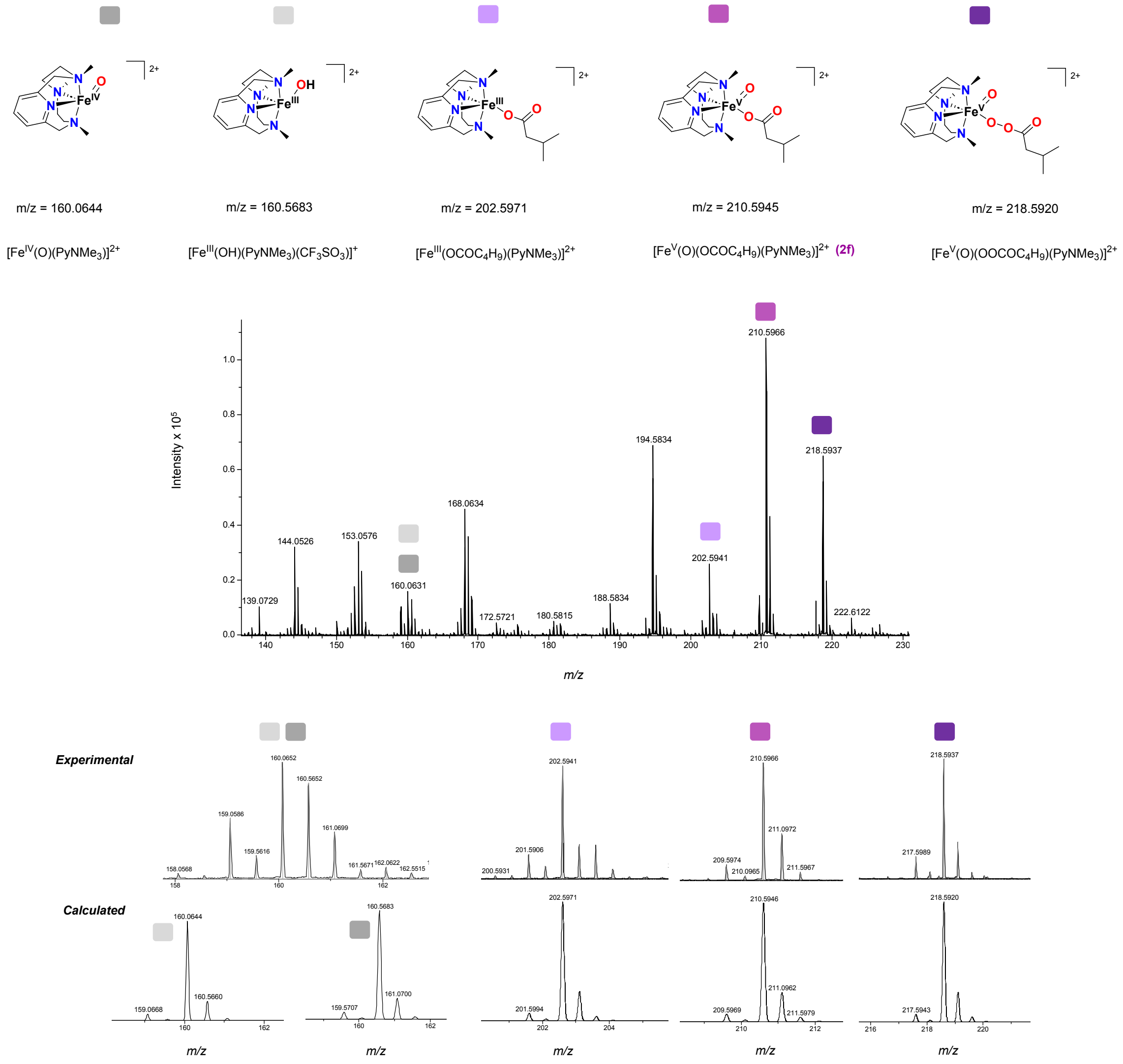

Figure S29. CSI-MS at -60 ºC in the 140 to 230 m/z region of **2f** generated by reaction of **1** with 10 equiv of isopervaleric acid in a mixture of acetone:acetonitrile 1:3.

5. Mössbauer and EPR spectroscopy of compounds 2e and 2f

Samples of **2e** and **2f** for EPR and Mössbauer measurements were prepared following the methodology described in section 3. The overall concentration of Fe in the samples was 1 mM. Samples of **2e** and **2f** 50% enriched with ^{57}Fe were prepared from 50% ^{57}Fe enriched **1**.[3]

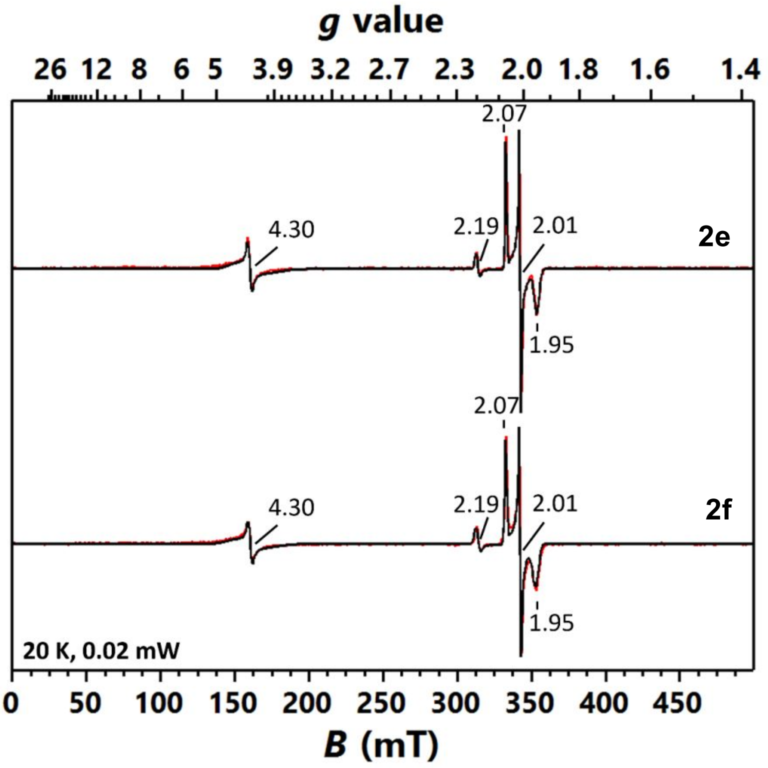

Figure S30. X-band EPR spectra of **2e** and **2f** recorded at T = 20 K in perpendicular mode between 0 and 500 mT. Red lines show experimental spectra and black lines correspond to their simulations. Simulation parameters can be found in Table S2.

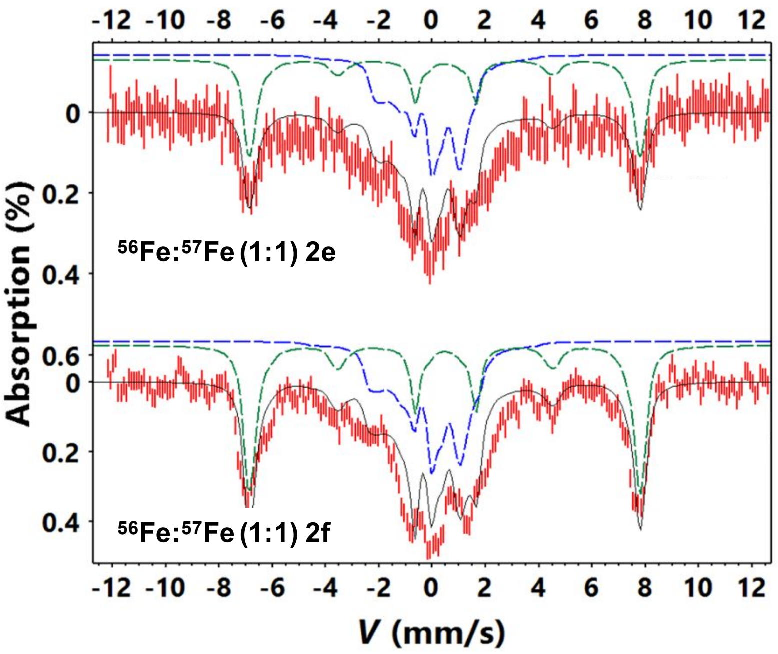

Figure S31. Mössbauer spectra of **2e** and **2f** samples 50% enriched with ^{57}Fe recorded at T = 4.2 K for B = 7.0 T applied perpendicular to the γ radiation. Red lines correspond to experimental data, black lines show overall simulations, green dotted lines show Fe^{III} components and blue lines correspond to Fe^{V} components. Simulation parameters can be found in Table S3.

Table S2. EPR simulation parameters used in Figures 2 and S22.

sample	site	*g*	σ*g*	***A*** (^{57}Fe, MHz)	conc (mM)
**2e**	Fe^{V} S = 1/2	[2.07, 2.01, 1.95]	[0.002, 0.001, 0.008]	-	0.287
	Fe^{III} S = 1/2	[2.20, 2.20, 1.93]	-	-	0.023
	Fe^{III} S = 5/2*	[2.01, 2.17, 1.80]	[-, 0.050, 0.050]	-	0.192
**^{56}Fe:^{57}Fe (1:1) 2e**	Fe^{V} S = 1/2	[2.07, 2.01, 1.95]	[0.003, -, 0.009]	[-, 52, -]	0.354
	Fe^{III} S =1/2	[2.20, 2.20, 1.93]	-	-	0.026
	Fe^{III} S = 5/2	Not measure			
**2f**	Fe^{V} S = 1/2	[2.07, 2.01, 1.95]	[0.004, 0.003, 0.010]	-	0.300
	Fe^{III} S = 1/2	[2.20, 2.19, 1.99]	-	-	0.030
	Fe^{III} S = 5/2	[2.01, 2.16, 1.83]	[0.007, 0.041, 0.036]	-	0.135
**^{56}Fe:^{57}Fe (1:1) 2f**	Fe^{V} S = 1/2**	[2.07, 2.01, 1.95]	[0.004, 0.002, 0.010]	[-, 56, -]	0.300
	Fe^{III} S = 1/2	[2.20, 2.19, 1.99]	-	-	0.031
	Fe^{III} S = 5/2	Not measure			

* D = +1 cm^{-1}, E/D = 0.333, σ(E/D) = 0.026
** D = +1 cm^{-1}, E/D = 0.333, σ(E/D) = 0.023

Table S3. Mössbauer simulation parameters used in Figure S23.

sample	site	D (cm^{-1})	E/D	δ (mm/s)	ΔE_Q (mm/s)	η	$A/g_N\beta_N$ (T)	*Area* (%)
**^{56}Fe:^{57}Fe (1:1) 2e**	Fe^{V}	-	-	-0.06	1.00	0.2	[-37.6, -5.1, -7.3]	40%
	Fe^{III}	+1	0.333	0.48	0.06	0	[-22.0, -21.1, -18.7]	29%
**^{56}Fe:^{57}Fe (1:1) 2f**	Fe^{V}	-	-	-0.06	1.00	0.2	[-40.5, -5.1, -7.3]	40%
	Fe^{III}	+1	0.333	0.48	0.06	0	[-22.0, -21.1, -18.7]	39%

6. Eyring plot

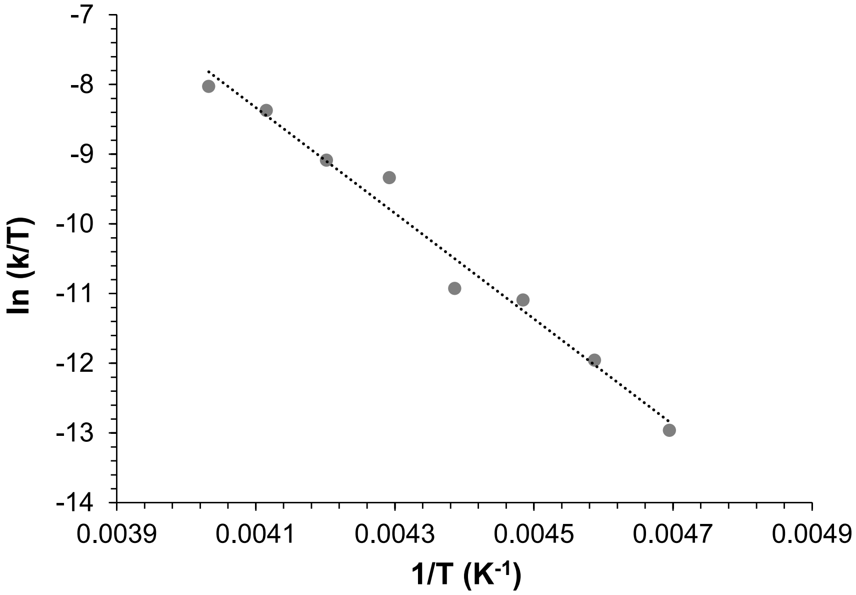

Figure S32. Eyring plot corresponding to the self-decay of **2d** in an acetone:acetonitrile 3:1 solvent mixture.

7. Isotope experiments

7.1. KIE determined from decay rates of two parallel reactions

To calculate the KIE from two parallel reactions, the self-decay of **2d** was fitted to a single exponential decay, obtaining an observed rate constant (k^{H}_{obs}). The same procedure was followed with its deuterated counterpart (***d*$_{17}$-2d**) affording k^{D}_{obs}. The ratio between both kinetic constants (k^{H}_{obs}/k^{D}_{obs}) affords the KIE.

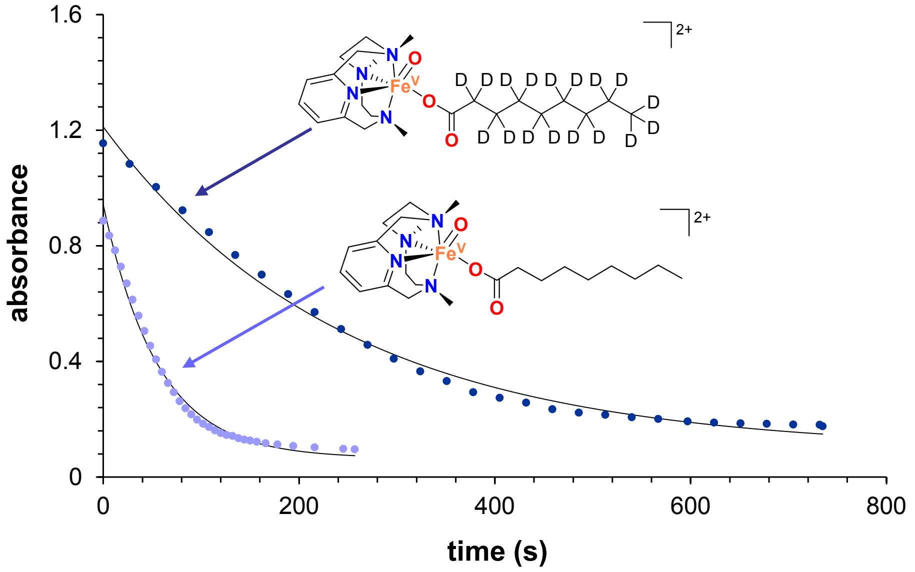

Figure S33. Kinetic trace at 497 nm corresponding to the decay of **2d** and ***d*$_{17}$-2d** by reaction of **1** (1 mM) with 4 equiv of **d** and ***d*$_{17}$-d**.

7.2. KIE determined from an intramolecular competition

For the determination of the KIE from an intramolecular competition, the iron(V)-oxo-carboxylato species was generated using singly-deuterated pernonanoic acid at the γ-position, namely pernonanoic-4-d$_1$ acid (***d*$_1$-d**). The self-decay of the resulting iron(V)-oxo-carboxylato species (***d*$_1$-2d**) affords both the deuterated and the non-deuterated γ-lactone. GC-MS analyses indicates that 67 % of the resulting γ-nonalactone is monodeuterated, which corresponds to a KIE value of 2.

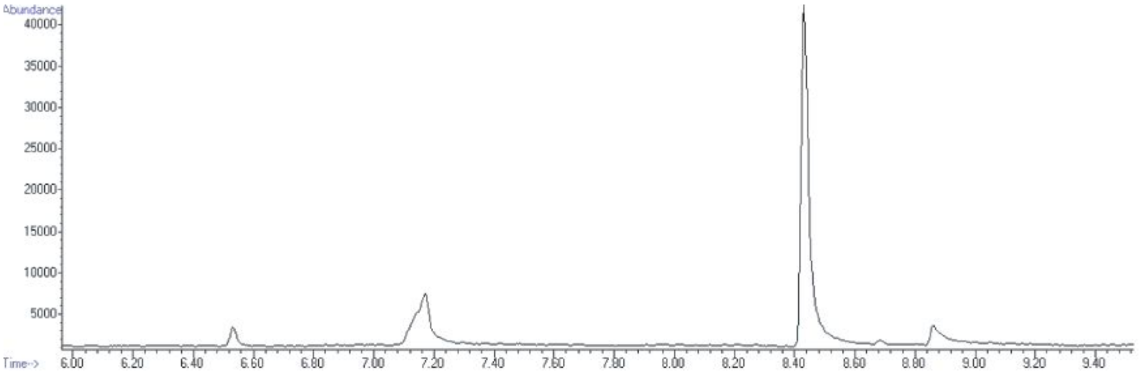

Figure S34. GC-MS (CI mode) chromatogram corresponding to the γ-nonalactone (retention time = 8.50 min) resulting from the decomposition of ***d*$_1$-2d**.

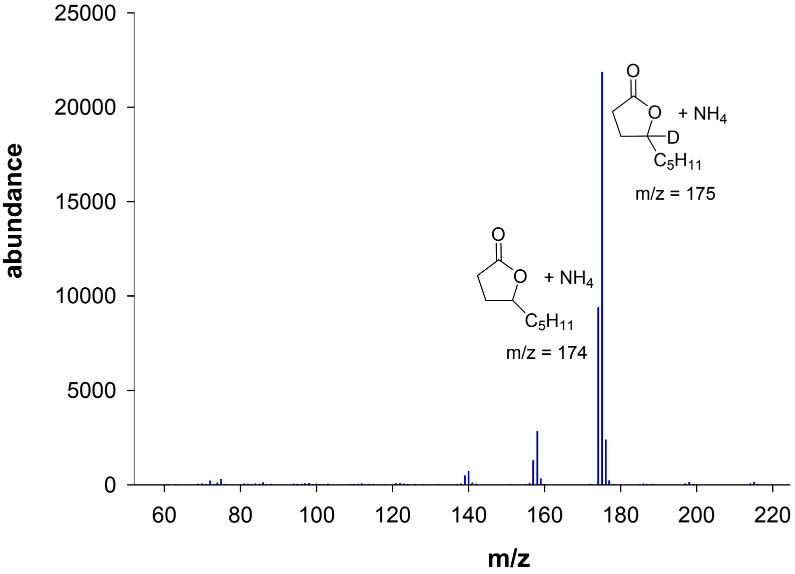

Figure S35. GC-MS spectrum of the peak at 8.50 min corresponding to the γ-nonalactone and the γ-nonalactone-4-d_1.

7.3. ^{18}O labelling experiments

To determine whether the reaction proceeds via a carboxylate rebound or a hydroxyl rebound, ^{18}O-labelling experiments were carried out. To do so, the iron(V)-oxo-carboxylato species was generated using pernonanoic-^{18}O acid (23 % ^{18}O-labelled in the oxygen atom of the carbonyl group). The self-decay of the resulting iron(V)-oxo-carboxylato species (**^{18}O-2d**) afforded both the ^{18}O-labelled and unlabelled γ–nonalactone. GC-MS analysis indicated that 23 % of the γ–nonalactone contained ^{18}O, indicating that the reaction proceeds through a carboxylate rebound after the hydrogen atom transfer step.

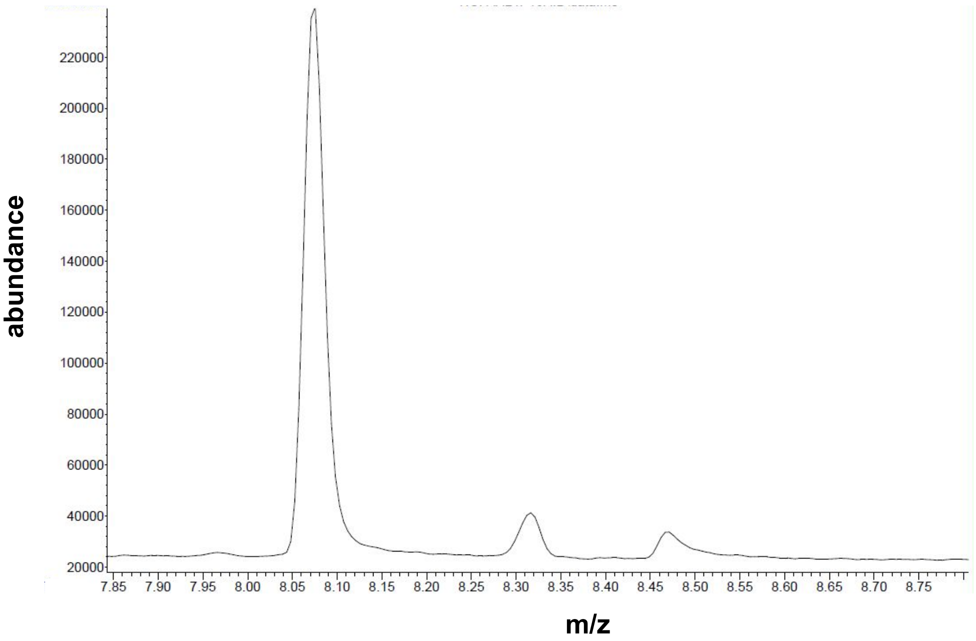

Figure S36. GC-MS (CI mode) chromatogram corresponding to the γ-nonalactone (retention time = 8.08 min) resulting from the decomposition of **^{18}O-2d**.

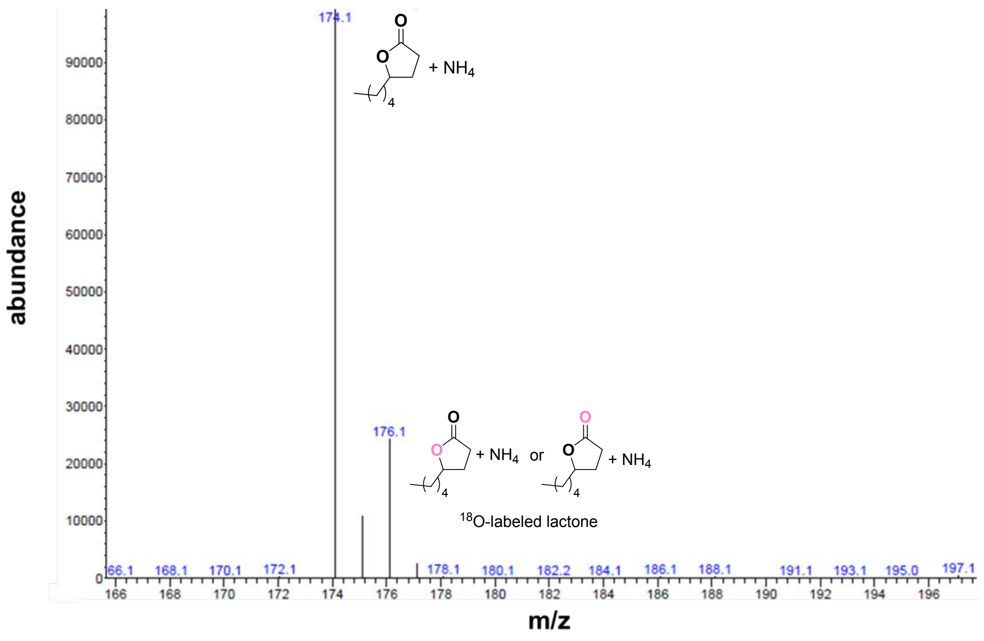

Figure S37. GC-MS spectrum of the peak at 8.08 min corresponding to the γ-nonalactone obtained from the decomposition of **^{18}O-2d** generated using pernonanoic-^{18}O acid (23 % ^{18}O-labelled in the oxygen atom of the carbonyl group). The calculated ^{18}O-content of the γ-nonalactone is 23 % according to the isotopic pattern.

8. Synthesis of other organic products

8.1. Synthesis of nonanoic-4-d_1 acid

Nonanoic-4-d_1 was prepared following a previously reported procedure (Scheme 1).[4]

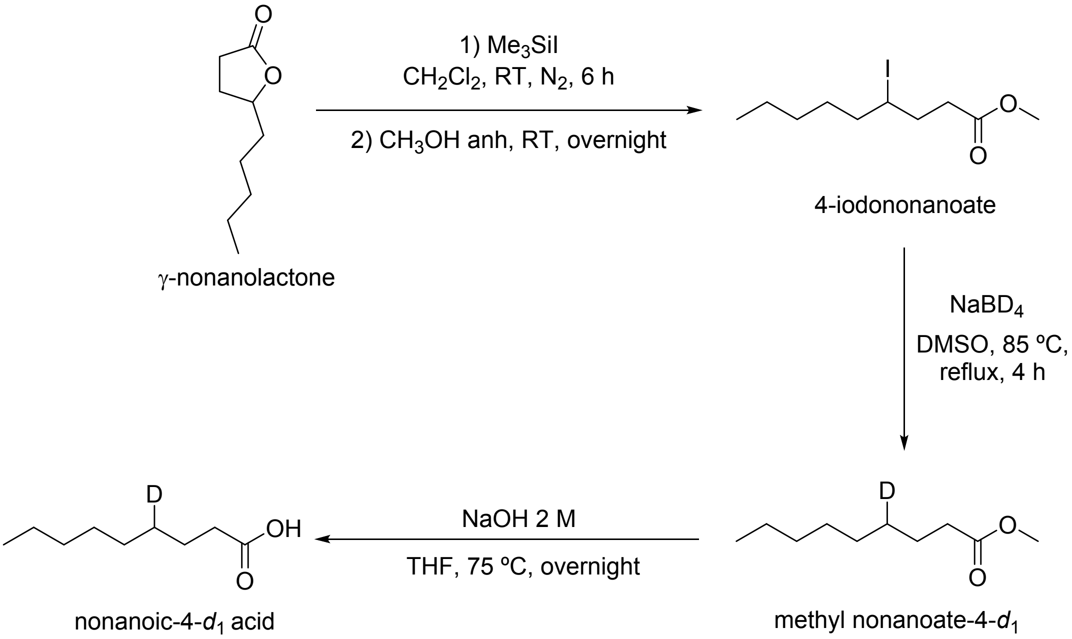

Scheme 1. Synthetic route for the synthesis of nonanoic-4-d_1 acid.

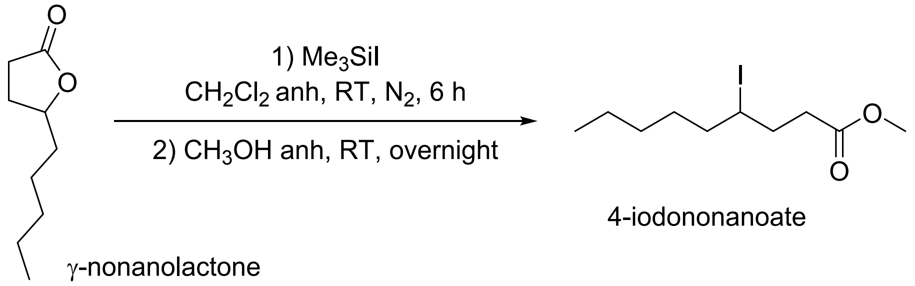

Synthesis of methyl 4-iodononanoate. Under a N_2 atmosphere, iodotrimethylsilane (2 mL, 15.2 mmol) was added dropwise to a solution of the γ-nonanolactone (0.59 g, 3.8 mmol) protected from light in anhydrous dichloromethane. The solution was stirred for 6 h at room temperature. Once the reaction was completed, which could be judged by ^{1}H-NMR, anhydrous methanol (0.77 mL, 19 mmol) was added to the mixture. The reaction was stirred at room temperature overnight. The reaction crude was diluted with dichloromethane (10 mL) and $Na_2S_2O_3$ 10% (25 mL) was also added. The aqueous phase was extracted with diethyl ether (3 x 100 mL). The organic phases were combined

and dried over $MgSO_4$ and the solvent was evaporated under reduced pressure. Afterwards, the product was purified by column chromatography on silica using 1 % diethyl ether/ hexane as the eluent to afford a yellowish oil (0.91 g, 81 % yield). ^{1}H-NMR ($CDCl_3$, 400 MHz, 298 K) δ, ppm: 4.12 (m, 1H), 3.69 (s, 3H), 2.66-2.44 (m, 2H), 2.07 (m, 2H), 1.89 (m, 1H), 1.71 (m, 1H), 1.54-1.25 (m, 6H), 0.90 (t, J = 6.9 Hz, 3H). ^{13}C-NMR ($CDCl_3$, 100 MHz) δ, ppm: 173.16, 51.73, 40.72, 38.35, 35.46, 34.19, 30.97, 29.13, 22.49, 14.13.

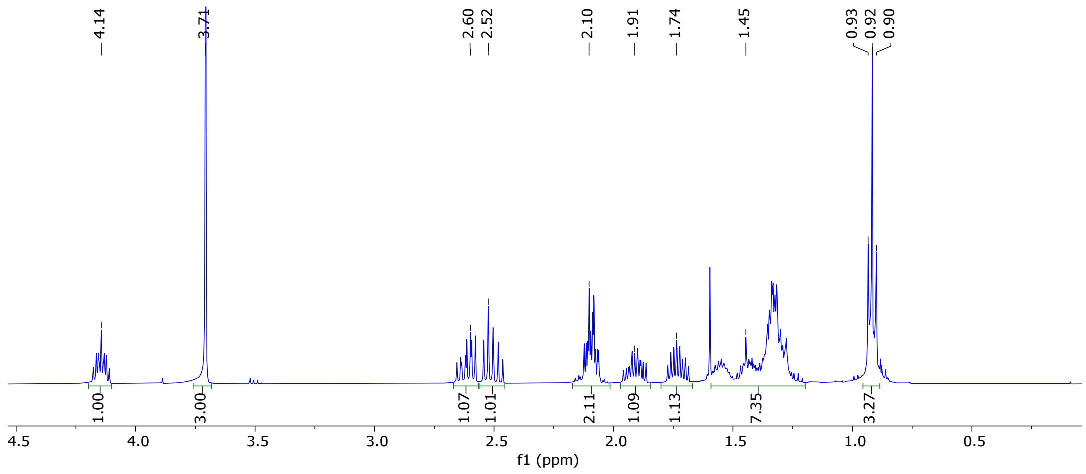

Figure S38. ^{1}H-NMR spectrum of the methyl 4-iodononanoate in $CDCl_3$ at 298 K.

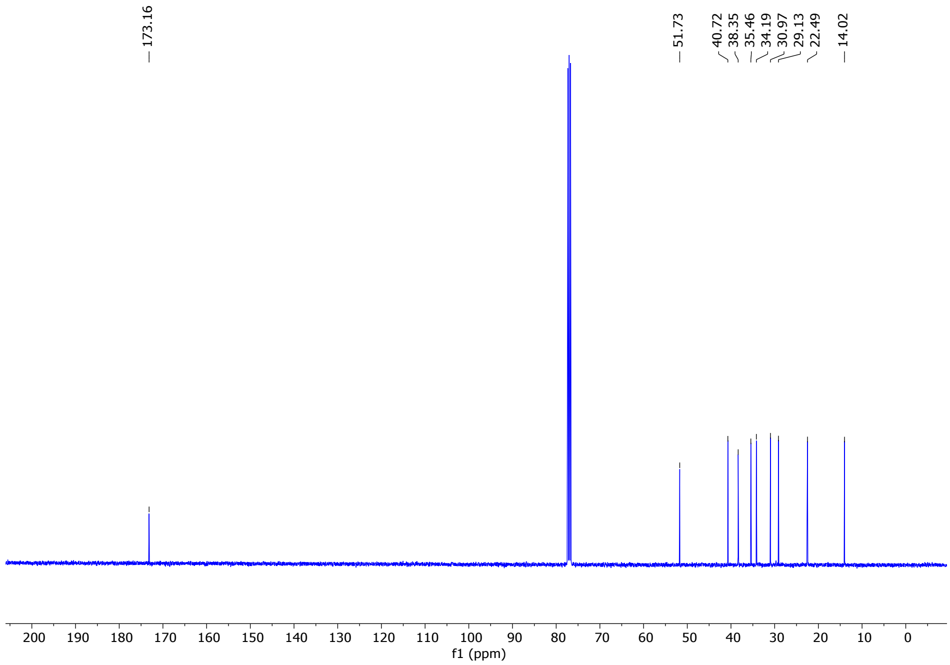

Figure S39. ^{13}C-NMR spectrum of the methyl 4-iodononanoate in $CDCl_3$ at 298 K.

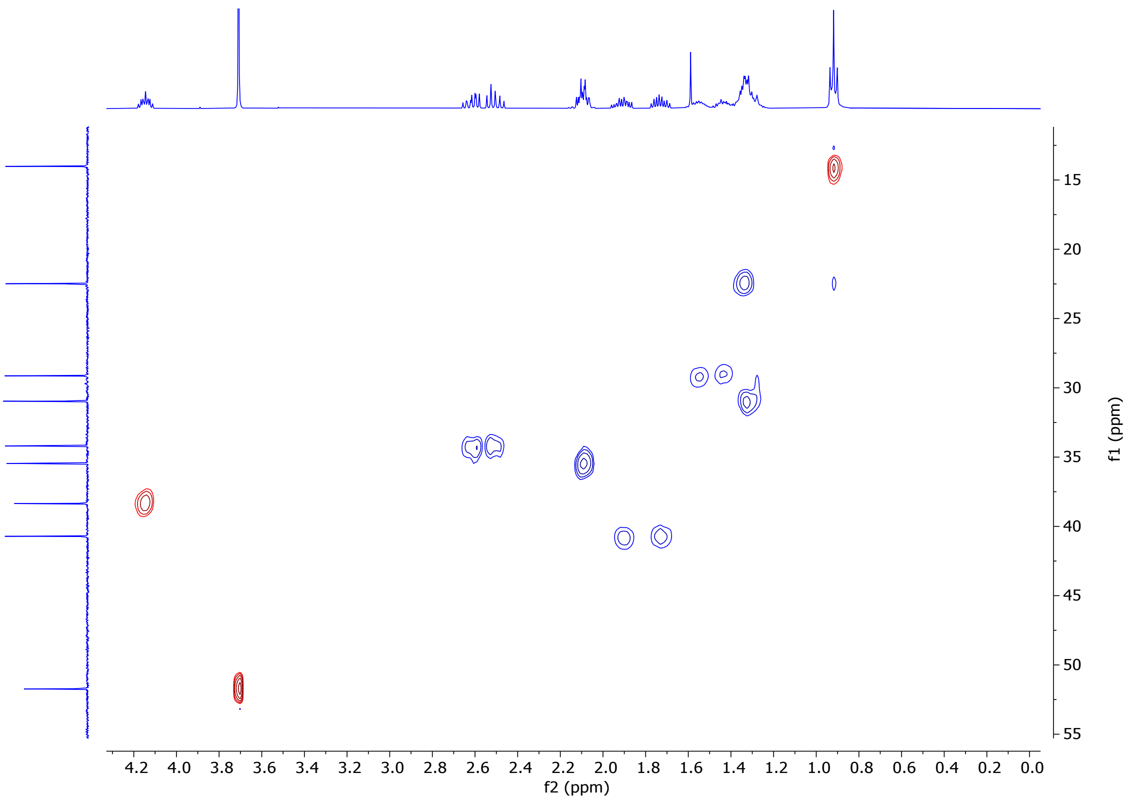

Figure S40. HSQCED spectrum of the methyl 4-iodononanoate in $CDCl_3$ at 298 K.

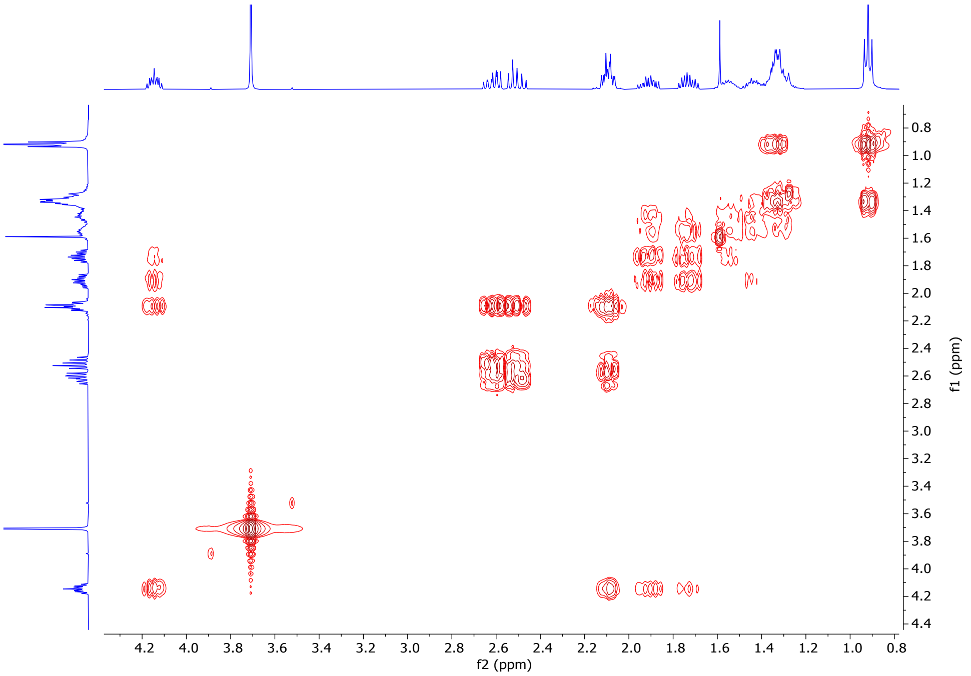

Figure S41. COSY spectrum of the methyl 4-iodononanoate in $CDCl_3$ at 298 K.

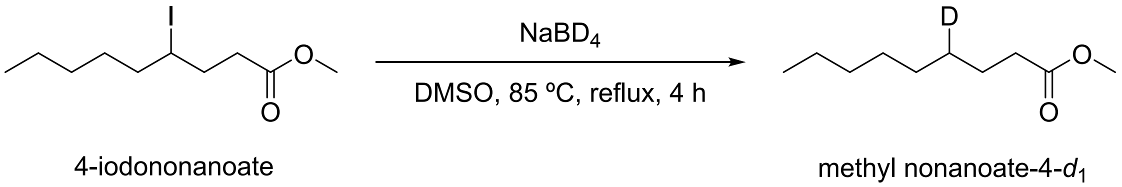

Synthesis of methyl nonanoate-4-d_1. In a 10 mL round-bottomed flask the iodo ester (0.41 g, 1.37 mmol) was dissolved in dimethylsulfoxide (6 mL) and sodium borodeuteride was added (0.12 g, 2.85 mmol). The mixture was stirred at 85 ºC for 4 h. Afterwards, the reaction crude was quenched with HCl 2 M (6 mL) and diluted with water (8 mL). The resulting mixture was extracted with hexane (3 x 20 mL). The combined organic phases were dried over $MgSO_4$, and the solvent was evaporated under reduced pressure. The product was further purified by column chromatography on silica using 2 % diethyl ether/hexane as the eluent to afford a colourless oil (0.10 g, 0.58 mmol, 43 % yield). ^{1}H-NMR ($CDCl_3$, 400 MHz, 298 K) δ, ppm: 3.66 (s, 3H), 2.32 (t, *J*= 7.6 Hz, 2H), 1.66-1.58 (m, 2H), 1.32-1.26 (m, 9H), 0.88 (t, *J*= 7.6 Hz, 3H). ^{13}C-NMR ($CDCl_3$, 100 MHz, 298 K) δ, ppm: 174.38, 51.45, 34.11, 31.82, 29.12, 29.10, 28.75, 24.87, 22.65, 14.10.

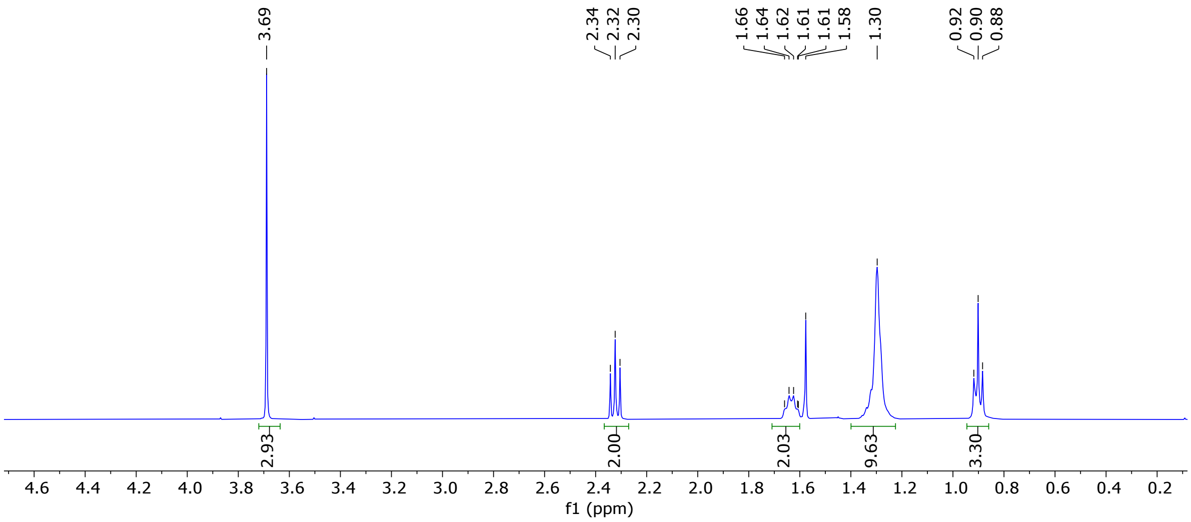

Figure S42. ^{1}H-NMR spectrum of methyl nonanoate-4-d_1 in $CDCl_3$ at 298 K.

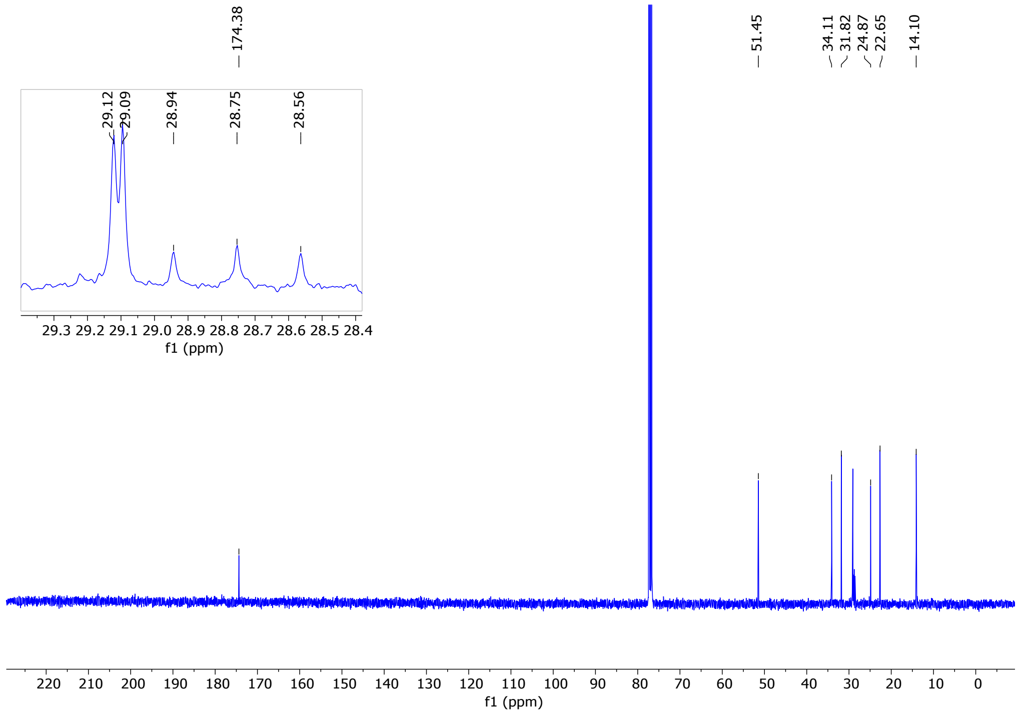

Figure S43. ^{13}C-NMR spectrum of methyl nonanoate-4-d_1 in $CDCl_3$ at 298 K.

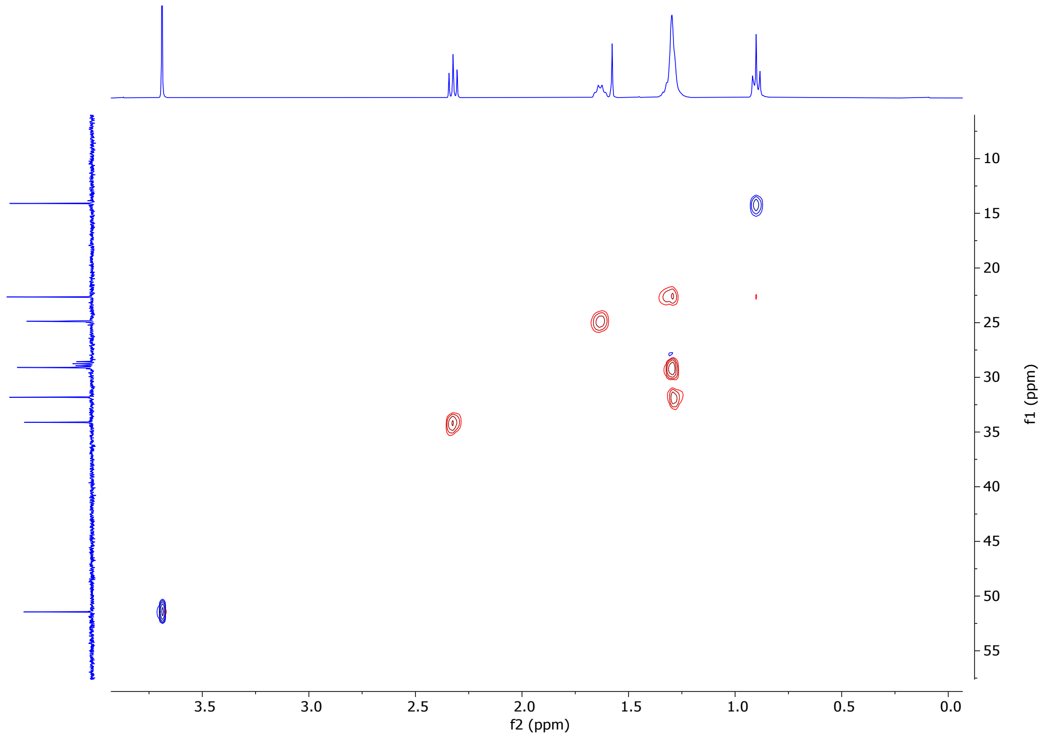

Figure S44. HSQCED spectrum of methyl nonanoate-4-d_1 in $CDCl_3$ at 298 K.

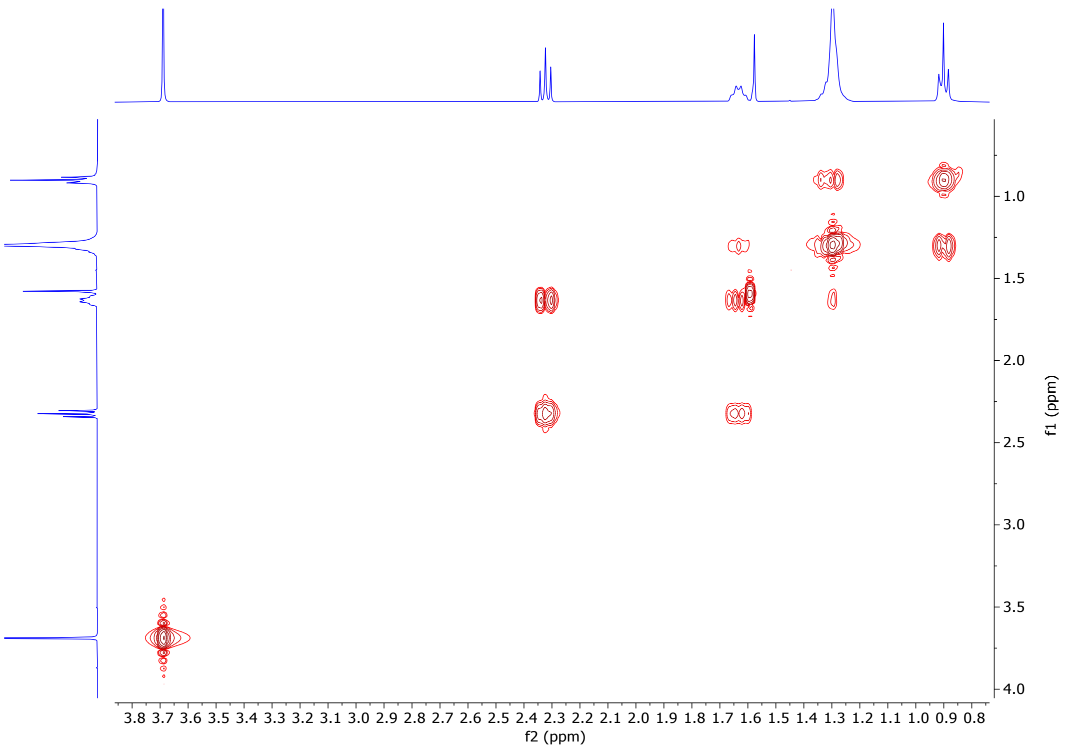

Figure S45. COSY spectrum of methyl nonanoate-4-d_1 in $CDCl_3$ at 298 K.

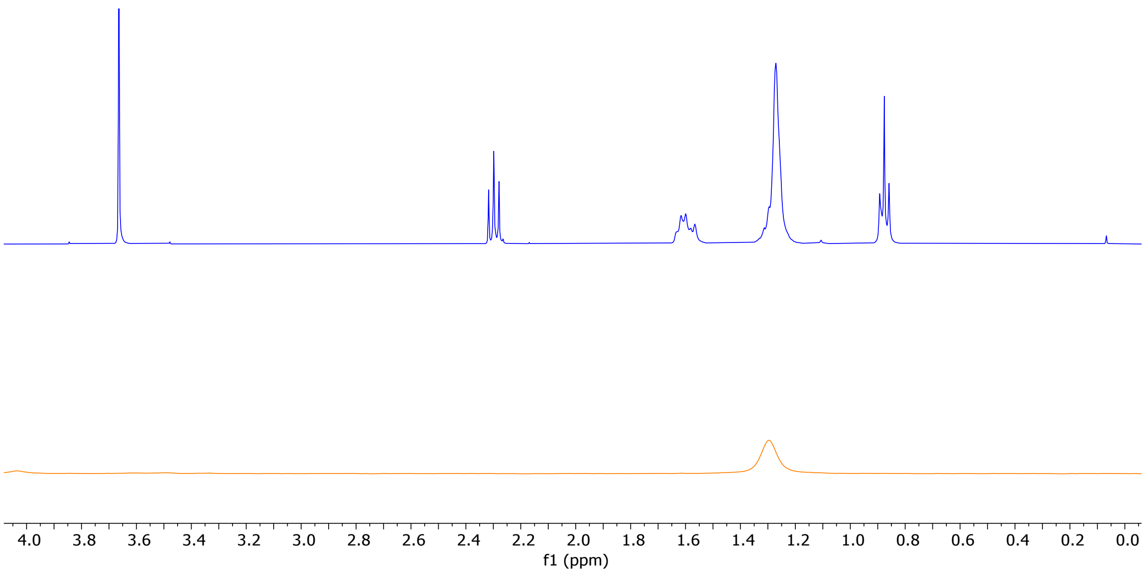

Figure S46. ^{1}H-NMR spectrum of methyl nonanoate-4-d_1 (top) and ^{2}H-NMR (bottom) in $CDCl_3$ at 298 K.

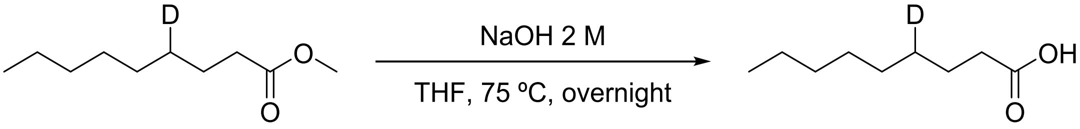

methyl nonanoate-4-d_1 nonanoic-4-d_1 acid

Synthesis of nonanoic-4-*d*$_1$ acid. In a 25 mL round-bottomed flask, methyl nonanoate-4-d_1 (0.060 g, 0.35 mmol) was dissolved in THF (5 mL) and NaOH 2 M (5 mL) was added to this solution. The resulting immiscible mixture was stirred at 75 ºC overnight. Afterwards, THF was removed under reduced pressure and the aqueous layer was washed with diethyl ether (2 x 10 mL). The aqueous layer was then acidified with HCl 2 M and extracted with diethyl ether (3 x 25 mL). The combined organic layers were dried over $MgSO_4$, and the solvent was removed under reduced pressure. The desired product was obtained as a colourless oil and no further purification was needed (0.043 g, 0.27 mmol, 77 % yield).^{1}H-NMR ($CDCl_3$, 400 MHz, 298 K) δ, ppm: 2.35 (t, J = 7.5 Hz, 2H), 1.63 (m, 2H), 1.32-1.23 (m, 9H), 0.88 (t, J= 7.5 Hz, 3H). ^{13}C-NMR ($CDCl_3$, 100 MHz, 298 K) δ, ppm: 33.52, 31.81, 29.10, 28.66, 24.61, 22.65, 14.1

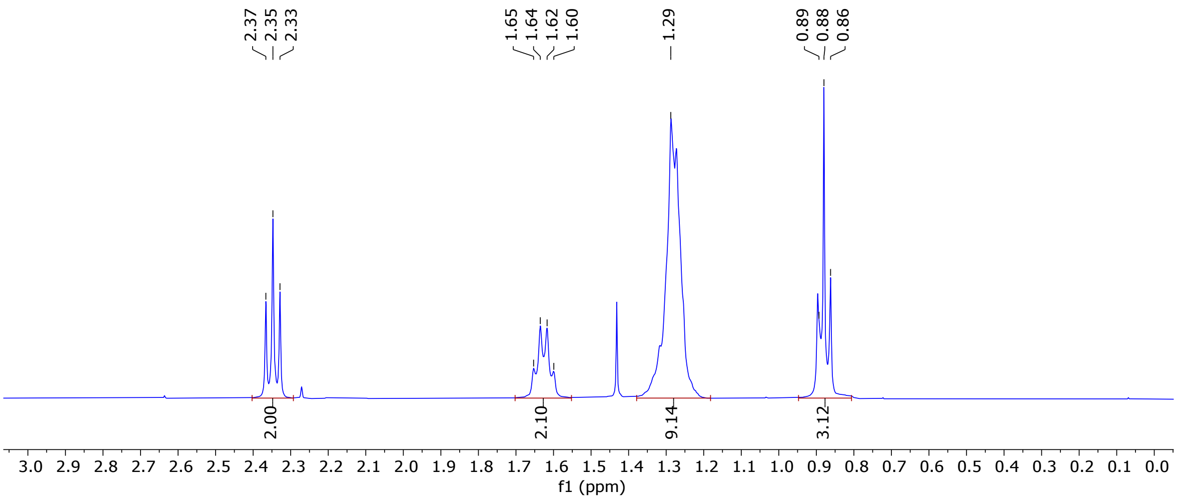

Figure S47. ^{1}H-NMR spectrum of nonanoic-4-d_1 acid in $CDCl_3$ at 298 K.

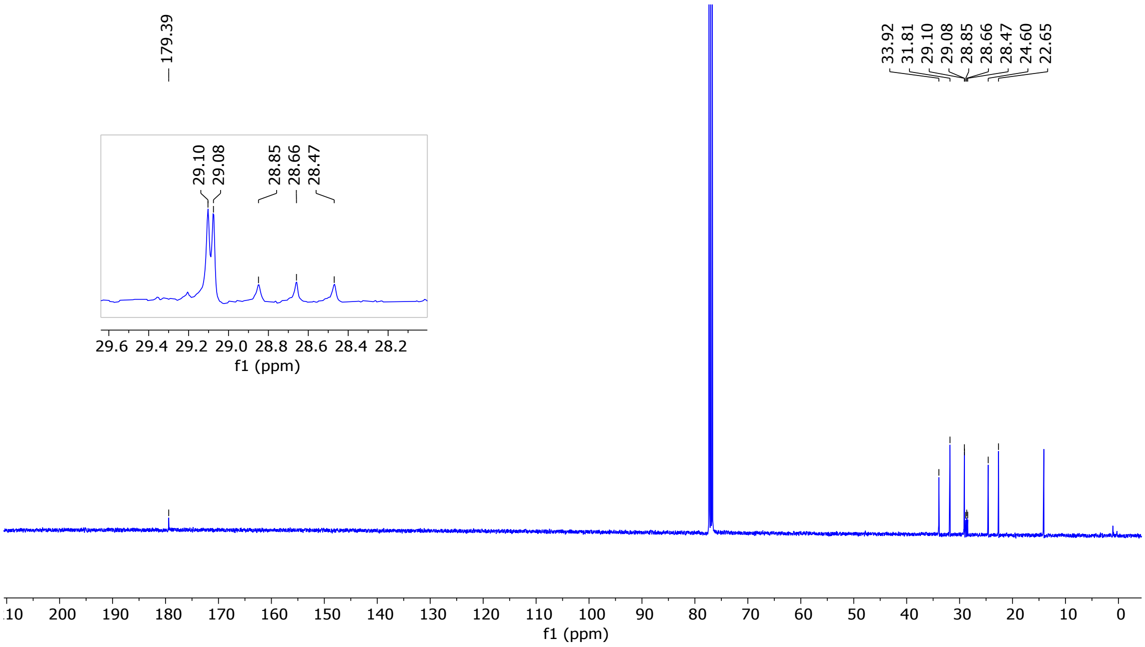

Figure S48. ^{13}C-NMR spectrum of nonanoic-4-d_1 acid in $CDCl_3$ at 298 K.

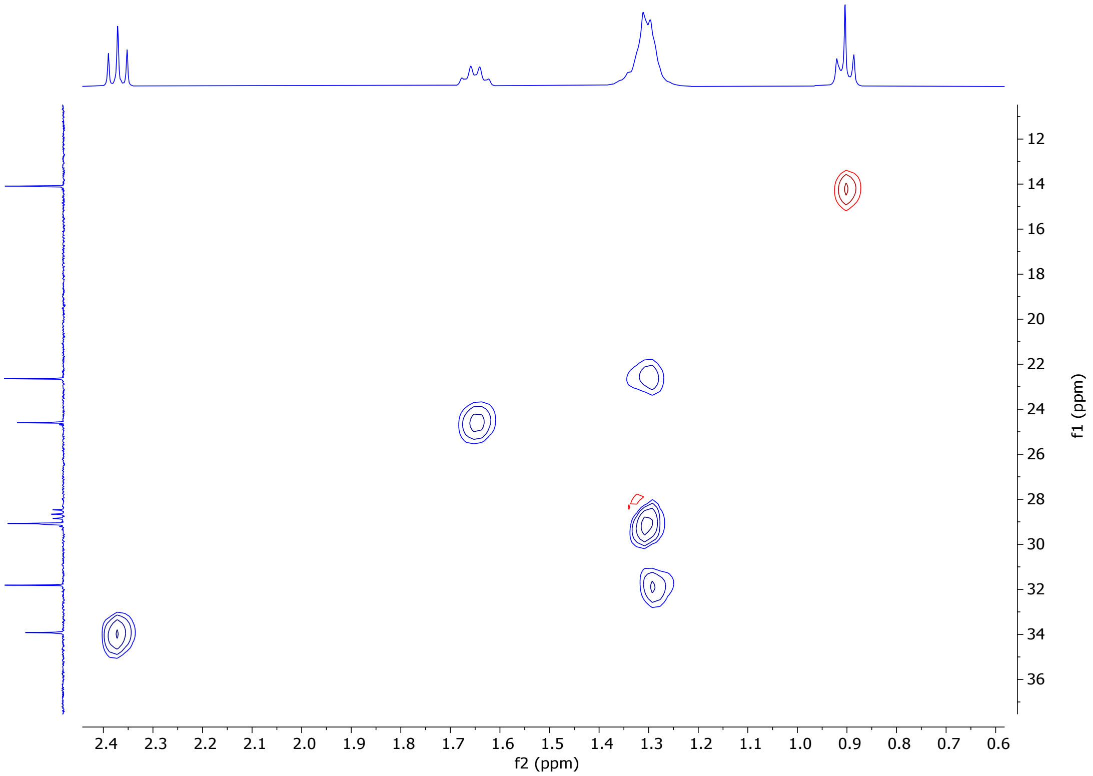

Figure S49. HSQCED spectrum of nonanoic-4-d_1 acid in $CDCl_3$ at 298 K.

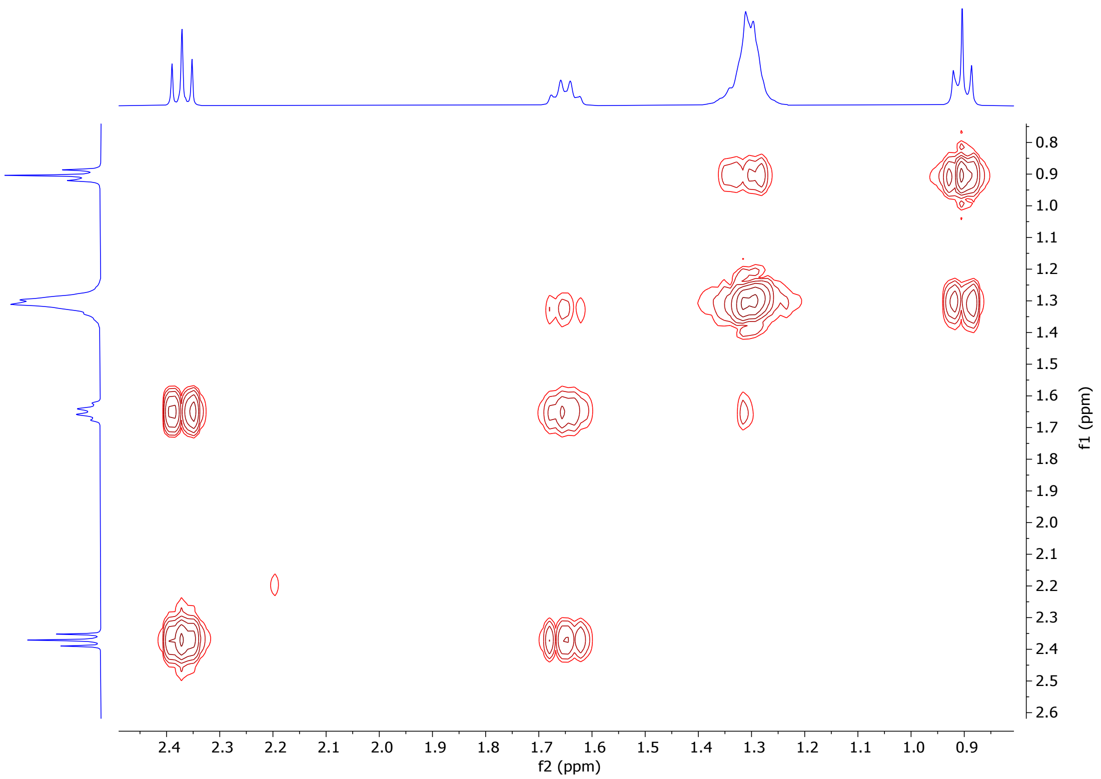

Figure S50. COSY spectrum of nonanoic-4-d_1 acid in $CDCl_3$ at 298 K.

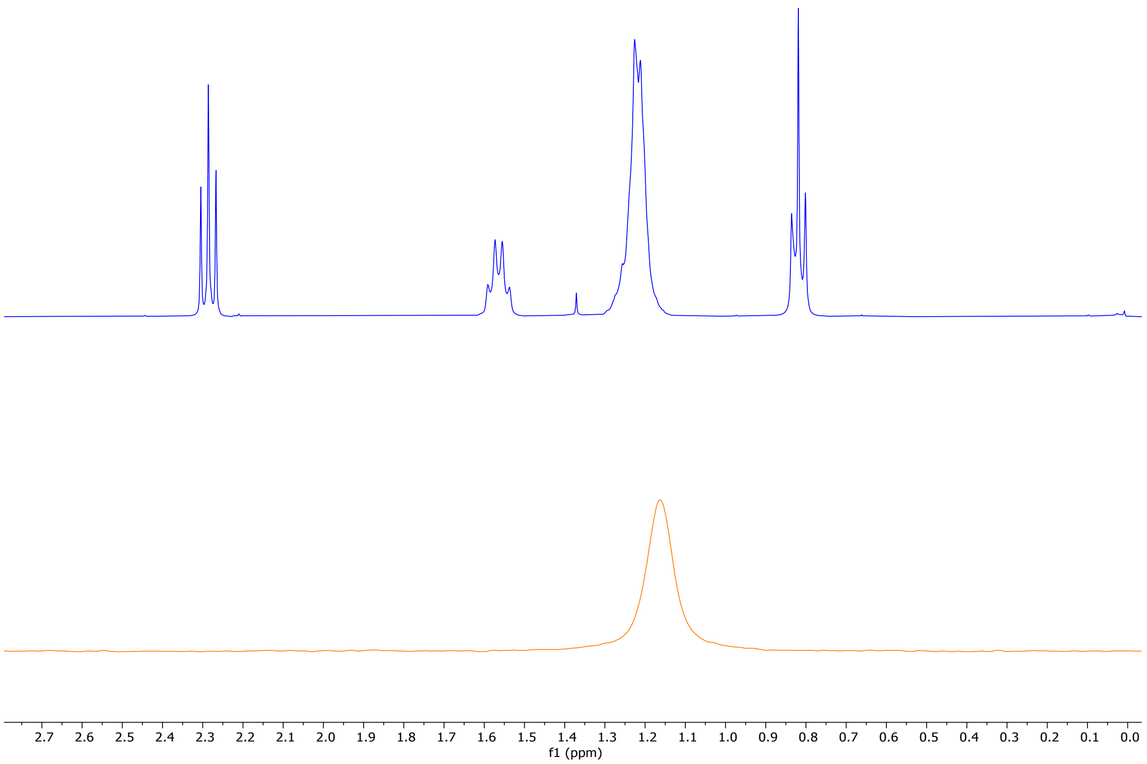

Figure S51. ^{1}H-NMR spectrum of nonanoic-4-d_1 acid (top) and ^{2}H-NMR (bottom) in $CDCl_3$ at 298 K.

8.2. Synthesis of 5-ethyl-5-methyldihydrofuran-2-one

5-ethyl-5-methyldihydrofuran-2-one was prepared following a previously reported procedure.[5]

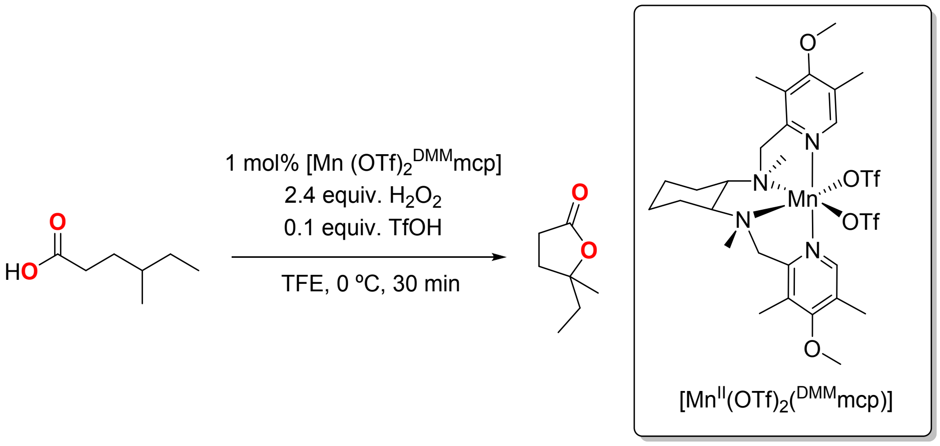

In a 100 mL round-bottomed flask 4-methylhexanoic acid (0.1g, 0.77 mmol) was added along with 1 mol% of Mn^{DMM}(mcp)[6] catalyst (6.1 mg, 0.0077 mmol) and trifluoroethanol (TFE, 50 mL). While stirring the mixture, 3.1 mL of a 0.6 M solution of H_2O_2 50% in H_2O (1.84 mmol, 2.4 equiv.) in TFE, and 3.1 mL of a 0.025 M solution of triflic acid (0.077 mmol, 0.1 equiv.) in TFE were added dropwise over a period of 30 min at 0 ºC by syringe pump. The resulting solution was stirred for 5 min and then quenched with isopropanol (500 μL). Saturated aqueous Na_2CO_3 solution was added (50 mL) and then the mixture was extracted with pentane (3 x 100 mL). The organic fractions were dried over $MgSO_4$ and then concentrated in vacuo (low pressure). The product was further purified by column chromatography on silica using pentane: diethyl ether 70:30 mixture as eluent, the product was afforded as a colourless oil (20 mg, 20 %). ^{1}H-NMR ($CDCl_3$, 400 MHz, 298 K) δ, ppm: 2.59 (m, 2H), 2.09 (m, 1H), 1.98 (m, 1H), 1.71(m, 2H), 1.38 (s, 3H), 0.97 (t, 3H).

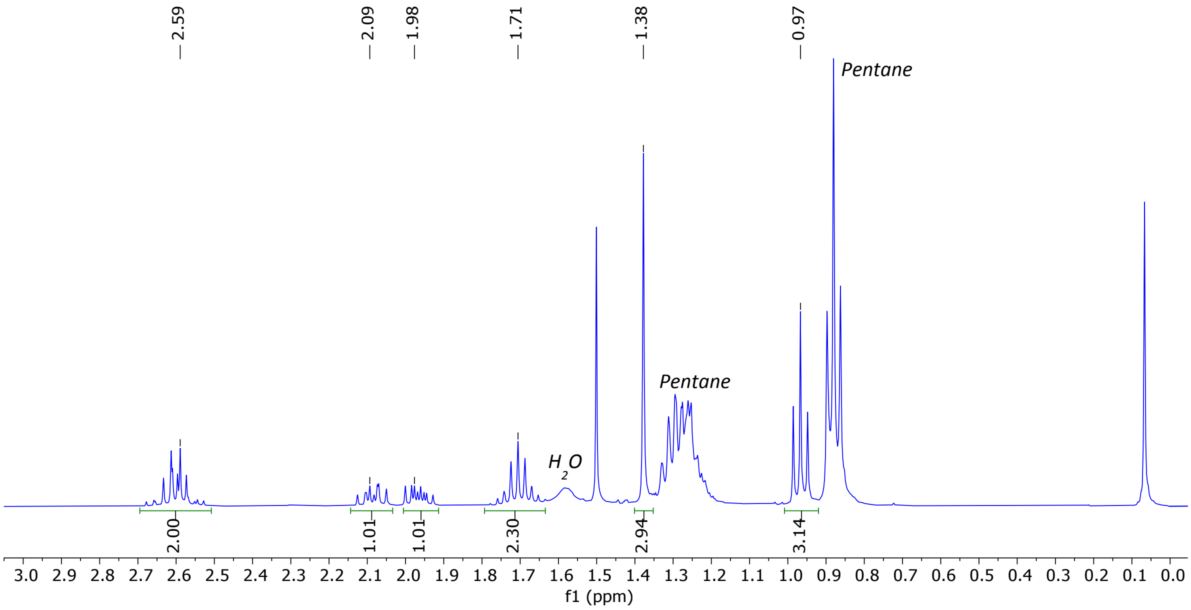

Figure S52. ^{1}H-NMR spectrum of 5-ethyl-5-methyldihydrofuran-2-one in $CDCl_3$ at 298 K.

8.3. Synthesis of (*S*)-4-methylhexanoic acid

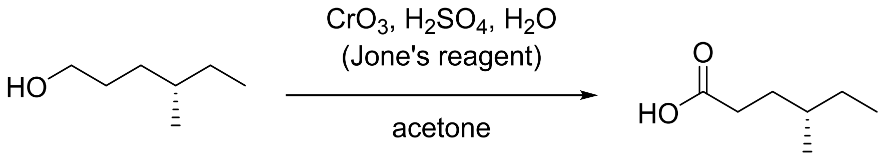

In a vial, (*S*)-4-methyl-1-hexanol (0.25 g, 1.72 mmol) were dissolved in acetone (3 mL). While stirring in an ice bath, Jones reagent was added dropwise until the mixture becomes orange. The reaction was then stirred for 20 min. Afterwards isopropanol was added dropwise until the mixture became blue. Water was added, and the mixture was washed with diethyl ether (3 x 20 mL). The organic fractions were extracted with a mixture of water: NaOH 2 M 50:50 (3 x 50 mL). The aqueous layers were combined and acidified with H_2SO_4 2 M, and then extracted with diethyl ether (3 x 10 mL). The organic layers were combined and dried over $MgSO_4$, and the solvent was removed in vacuo. The product was further purified by column chromatography on silica using ethyl acetate: hexane 10:90 mixture as eluent and was obtained as a yellowish oil (0.20 g, 71 % yield). ^{1}H-NMR ($CDCl_3$, 400 MHz, 298 K) δ, ppm: 2.37 (m, 2H), 1.70 (m, 1H), 1.38 (m, 3H), 1.19 (m, 1H) 0.9 (m, 6H)

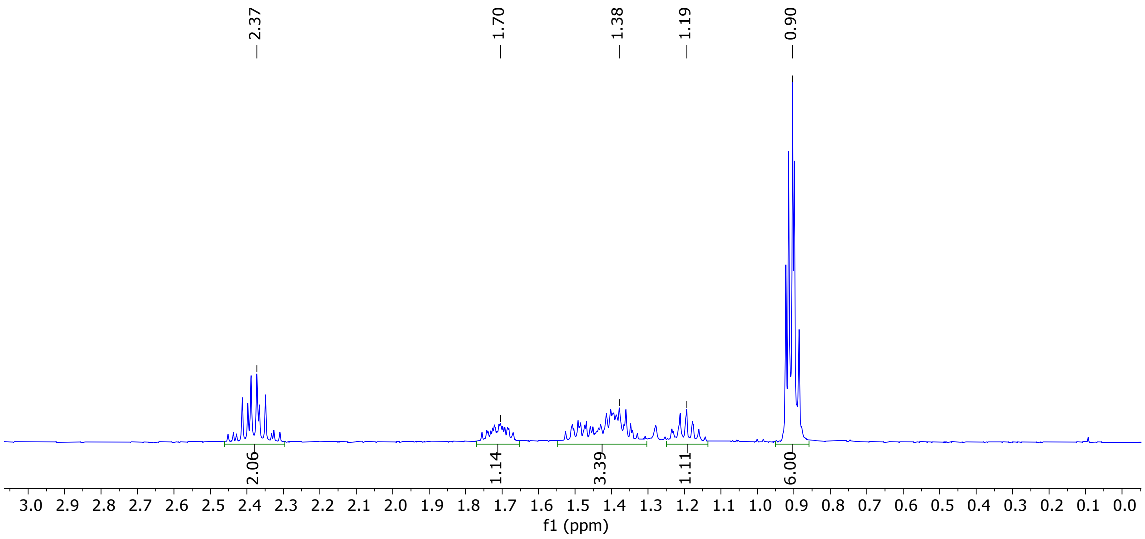

Figure S53. ^{1}H-NMR spectrum of (*S*)-4-methylhexanoic acid in $CDCl_3$ at 298 K.

8.4. Synthesis of nonanoic-^{18}O acid

In a vial, nonanoic acid (64.6 mg, 0.41 mmol) was mixed with methanesulfonic acid (646 μL, 9.9 mmol) at room temperature. Then $H_2{}^{18}O$ (408 μL, 50 equiv) was added dropwise to the mixture, which was subsequently stirred at 40 ºC overnight. Afterwards, ice was added along with a saturated aqueous solution of $(NH_4)_2SO_4$ (1 mL) and the mixture was extracted with dichloromethane (2 x 1 mL). The combined organic layers were washed with an aqueous solution of $(NH_4)_2SO_4$ (2 x 2 mL). Afterwards, the organic fraction was dried with $MgSO_4$ and the solution was filtered through Celite©. Finally, the solution was dried under vacuum affording a transparent oil (56.1 mg, 87 % yield). ^{1}H-NMR ($CDCl_3$, 400 MHz, 298 K) δ, ppm: 2.35 (t, *J* = 8 Hz, 2H), 1.67-1.60 (q, *J* = 8 Hz, 2H), 1.38-1.21 (m, 10H), 0.90-0.86 (t, 3H). According to ESI-MS analysis, the ^{18}O-content of the prepared nonanoic-^{18}O acid was 10 % singly ^{18}O-labelled and 86 % doubly ^{18}O-labelled (Figure S55).

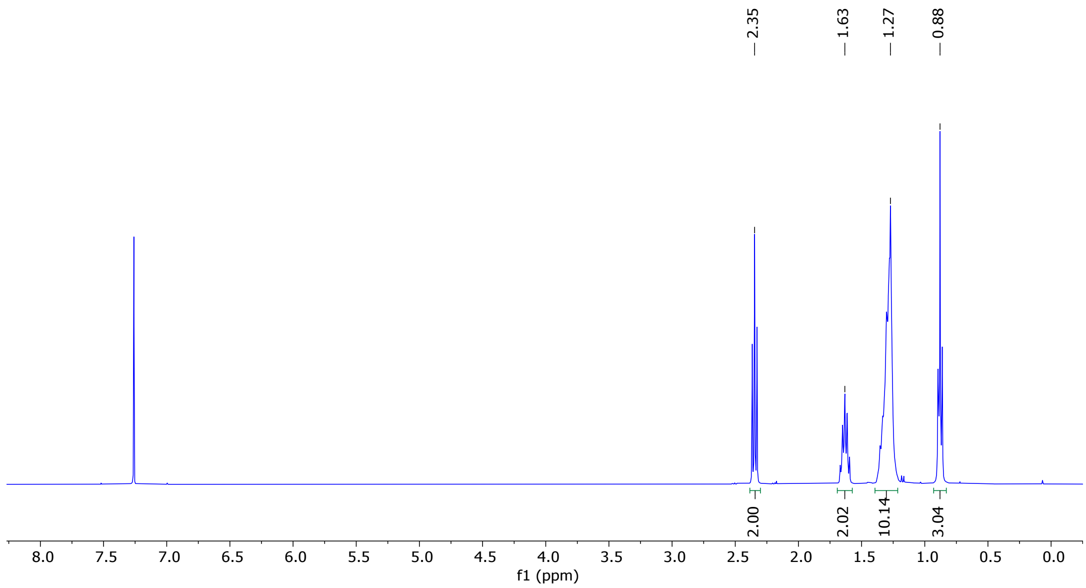

Figure S54. ^{1}H-NMR spectrum of nonanoic-^{18}O acid in $CDCl_3$ at 298 K.

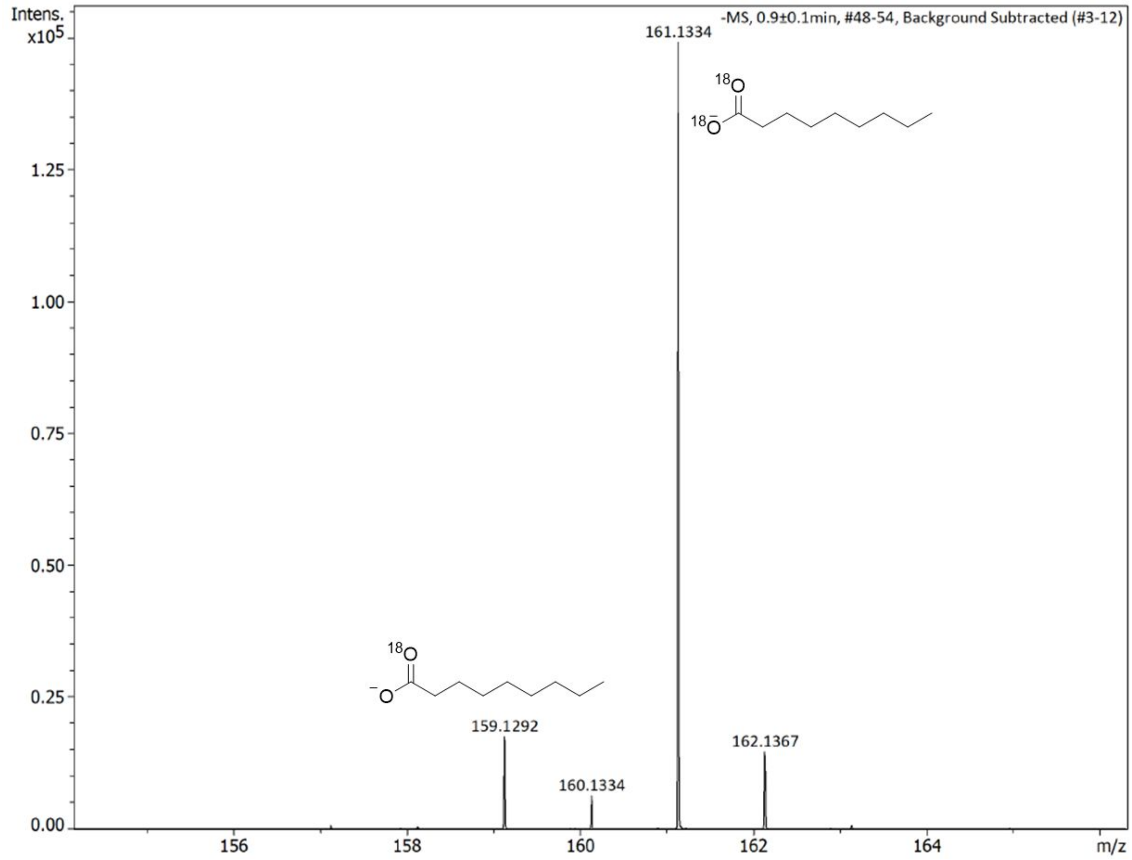

Figure S55. ESI-MS spectrum of nonanoic-^{18}O acid. The calculated ^{18}O-content according to the isotopic pattern is 10 % singly ^{18}O-labelled and 86 % doubly ^{18}O-labelled.

9. DFT calculations

9.1. Computational details for the electronic structure calculations

All DFT calculations were performed by using the Gaussian16 program.[7] The optimization of the geometry of all studied minimum and transition state structures were calculated employing density functional theory (DFT) using UB3LYP functional[8-9] together with dispersion corrections developed by Grimme and coworkers with the Becke-Jonhson damping[10-12] and a double-ζ basis-set of Ahlrichs and co-workers, Def2SVP,[13] and SMD solvation model for acetonitrile. All stationary points were confirmed by performing analytical Hessian calculations employing the same level of theory, basis set, and solvent (i.e. all the minimum present only real frequencies, while the transition state present only one imaginary frequency corresponding to the reaction coordinate). We checked with IRC calculations that the Transition States are connected to the respective reactants and products. Thermal quasiharmonic corrections of the enthalpic and entropic corrections to the Gibbs enthalpy of stationary point geometries were computed using Grimme's algorithm using the GoodVibes code.[14-15,18] Single-point electronic energy corrections were performed with UB3LYP functional[8-9] together with dispersion corrections developed by Grimme and coworkers with the Becke-Jonhson damping,[10-12] def2TZVP[16] basis set, and SMD solvation model for acetonitrile. Therefore, the whole methodology of the study can be denoted as UB3LYP-D3BJ/Def2TZVP/SMD//UB3LYP-D3BJ/Def2SVP/SMD.

9.2. Spin density

Table S4. Spin density computed at UB3LYP-D3BJ/Def2SVP/SMD(acetonitrile) level of theory.

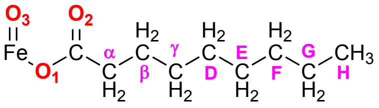

compound **I**

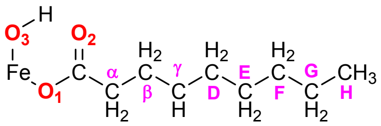

compound **II**

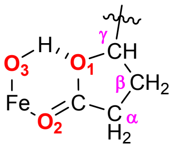

compound ^{trans}**III**

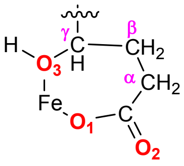

compound ^{cis}**III**

	Fe	O_1	O_2	O_3	Cγ
$^{cis}\mathbf{I}_{d}^{c}$	1.511 (1.52)[a]	-0.035	-0.419 (-0.38)[a]	0.091 (0.00)[a]	0.000
$^{trans}\mathbf{I}_{d}^{c,d}$	1.522 (1.50)[a]	-0.038	-0.408 (-0.37)[a]	0.065 (0.00)[a]	-0.001
$^{cis}\mathbf{I}_{q}^{c}$	1.900	0.071	0.132	1.060	0.001
$^{cis}\mathbf{I}_{q,IRC}^{d}$	1.926	0.090	0.103	1.046	0.000
$^{trans}\mathbf{I}_{q}^{c,d}$	2.010 (1.99)[a]	0.071	0.082 (0.08)[a]	1.030 (0.95)[a]	0.000
$^{cis}\mathbf{I}_{s}^{c}$	3.249	0.050	0.587	0.816	0.001
$^{trans}\mathbf{I}_{s}^{c}$	3.158	0.046	0.723	0.727	0.000
$^{cis}\mathbf{TS(I\text{-}III)}_{q}$	1.777	0.123	0.019	0.830	0.344
$^{trans}\mathbf{TS(I\text{-}III)}_{q}$	1.819	0.081	0.012	0.834	0.356
$^{cis}\mathbf{TS(I\text{-}III)}_{d}$ [b]	1.625	0.112	0.001	-0.509	-0.056
$^{trans}\mathbf{TS(I\text{-}III)}_{d}$	1.899	-0.002	0.011	-0.635	-0.028
$^{cis}\mathbf{II}_{d}$	1.016	-0.005	-0.001	0.099	0.000
$^{trans}\mathbf{II}_{d}$	0.972	-0.011	0.002	0.110	0.000
$^{cis}\mathbf{IRC1}_{q}$	1.968	0.051	0.011	0.252	0.902
$^{cis}\mathbf{IRC2}_{q}$	2.793	0.031	0.008	0.075	-0.061
$^{cis}\mathbf{II}_{q}$	2.793	0.043	0.000	0.032	0.000
$^{trans}\mathbf{IRC1}_{q}$	2.002	-0.008	0.011	0.259	0.936
$^{trans}\mathbf{IRC2}_{q}$	2.787	0.014	0.005	0.068	-0.020
$^{trans}\mathbf{II}_{q}$	2.807	0.008	0.009	0.065	0.000
$^{cis}\mathbf{II}_{s}$	4.165	0.078	0.015	0.367	0.006
$^{trans}\mathbf{II}_{s}$	4.169	0.114	0.018	0.299	0.001
$^{cis}\mathbf{TS(II\text{-}III)}_{s}$	4.165	0.094	0.020	0.359	0.001
$^{cis}\mathbf{III}_{d}^{c,d}$	1.027	0.081	0.004	-0.010	0.001
$^{trans}\mathbf{III}_{d}^{c}$	0.922	0.000	-0.006	0.145	0.000
$^{cis}\mathbf{III}_{q}^{c,d}$	2.874	0.052	0.001	0.036	0.003
$^{trans}\mathbf{III}_{q}^{c}$	2.825	0.003	0.002	0.057	0.000
$^{trans}\mathbf{III}_{q,IRC}^{d}$	2.816	0.007	0.001	0.180	0.000
$^{cis}\mathbf{III}_{s}^{c,d}$	4.164	0.216	0.048	0.061	0.002
$^{trans}\mathbf{III}_{s}^{c}$	4.165	0.003	0.046	0.337	0.000

[a] B. Mondal, F. Neese, E. Bill, and S. Ye, *J. Am. Chem. Soc.* **2018**, *140*, 9531−9544.[17] [b] After several attempts we could not find the optimized geometry of this TS due to its very low imaginary frequency. The data presented correspond to geometry of the maximum of a relaxed scan connecting $^{cis}\mathbf{I}_{q}$ and $^{cis}\mathbf{II}_{q}$. [c] Minimum Gibbs energy conformer. [d] IRC conformer.

9.3. Key geometrical and IR parameters

Table S5. Fe-O and O-O bond distances and Fe-O stretching frequency computed at UB3LYP-D3BJ/Def2SVP/SMD(acetonitrile) level of theory.

	$Fe\text{-}O_3$ (Å)	$\nu_{Fe\text{-}O3}$ (cm^{-1})	$Fe\text{-}O_1$ (Å)	$O_3\text{-}O_2$ (Å)
$^{cis}\mathbf{I}_d{}^{c}$	1.636	825	1.913	2.009
$^{trans}\mathbf{I}_d{}^{c,d}$	1.632 (1.631)[a]	838 (804)[a]	1.927	2.029 (2.07)[a]
$^{cis}\mathbf{I}_q{}^{c}$	1.661	758	1.819	2.470
$^{cis}\mathbf{I}_{q,IRC}{}^{d}$	1.654	771	1.810	2.547
$^{trans}\mathbf{I}_q{}^{c,d}$	1.647	807	1.835	2.610 (2.583)[a]
$^{cis}\mathbf{I}_s{}^{c}$	1.688	703	1.973	2.225
$^{trans}\mathbf{I}_s{}^{c}$	1.644	803	2.074	2.466
$^{cis}\mathbf{TS(I\text{-}III)}_q$	1.734	-	1.828	4.169
$^{trans}\mathbf{TS(I\text{-}III)}_q$	1.726	-	1.850	4.054
$^{cis}\mathbf{TS(I\text{-}III)}_d{}^{b}$	1.645	-	1.824	4.128
$^{trans}\mathbf{TS(I\text{-}III)}_d$	1.681	-	1.875	4.296
$^{cis}\mathbf{II}_d$	1.810	604	1.972	4.075
$^{trans}\mathbf{II}_d$	1.815	628	1.954	4.376
$^{cis}\mathbf{II}_q$	1.824	613	1.893	3.709
$^{trans}\mathbf{II}_q$	1.798	640	1.947	4.240
$^{cis}\mathbf{II}_s$	1.840	628	2.034	2.671
$^{trans}\mathbf{II}_s$	1.835	657	1.972	4.528
$^{cis}\mathbf{TS(II\text{-}III)}_s$	1.843	624	1.986	2.722
$^{cis}\mathbf{III}_d{}^{c,d}$	2.023	-	1.844	4.152
$^{trans}\mathbf{III}_d{}^{c}$	1.796	653	2.024	2.825
$^{cis}\mathbf{III}_q{}^{c,d}$	2.143	-	1.837	4.193
$^{trans}\mathbf{III}_q{}^{c}$	1.787	676	2.081	2.851
$^{trans}\mathbf{III}_{q,IRC}{}^{d}$	1.777	688	2.832	4.523
$^{cis}\mathbf{III}_s{}^{c,d}$	2.097	-	1.847	4.388
$^{trans}\mathbf{III}_s{}^{c}$	1.819	681	2.180	2.864

[a] B. Mondal, F. Neese, E. Bill, and S. Ye, *J. Am. Chem. Soc.* **2018**, *140*, 9531−9544.[17] [b] After several attempts we could not find the optimized geometry of this TS due to its very low imaginary frequency. The data presented correspond to geometry of the maximum of a relaxed scan connecting $^{cis}\mathbf{I}_q$ and $^{cis}\mathbf{II}_q$. [c] Minimum Gibbs energy conformer. [d] IRC conformer.

9.4. Electronic energies, thermal and entropic corrections, and Gibbs energies

Table S6. Absolute and relative electronic energies of the optimized structures computed at UB3LYP-D3BJ/Def2SVP/SMD(acetonitrile) level and single point energies computed at UB3LYP-D3BJ/Def2TZVP/SMD(acetonitrile) level of theory.

	**Electronic energy Def2SVP (a.u.)**	**Rel. Elec. energy Def2SVP (kcal·mol^{-1})**	**Electronic energy Def2TZVP (a.u.)**	**Rel. Elec. energy Def2TZVP (kcal·mol^{-1})**
$^{cis}\mathbf{I_d}^{b}$	-2608.3535594	0.00	-2609.9992383	0.00
$^{trans}\mathbf{I_d}^{b,c}$	-2608.3533019	0.16	-2609.9998484	-0.38
$^{cis}\mathbf{I_q}^{b}$	-2608.3468341	4.22	-2609.9948303	2.77
$^{cis}\mathbf{I_{q,IRC}}^{c}$	-2608.3460726	4.70	-2609.9942179	3.15
$^{trans}\mathbf{I_q}^{b,c}$	-2608.3473343	3.91	-2609.9955435	2.32
$^{cis}\mathbf{I_s}^{b}$	-2608.3250388	17.90	-2609.9718619	17.18
$^{trans}\mathbf{I_s}^{b}$	-2608.3341404	12.19	-2609.9794904	12.39
$^{cis}\mathbf{TS(I\text{-}III)_q}$	-2608.3263933	17.05	-2609.9714311	17.45
$^{trans}\mathbf{TS(I\text{-}III)_q}$	-2608.3268758	16.74	-2609.9717623	17.24
$^{cis}\mathbf{TS(I\text{-}III)_d}^{a}$	-2608.3228924	19.24	-2609.9620448	23.34
$^{trans}\mathbf{TS(I\text{-}III)_d}$	-2608.3305384	14.45	-2609.9642546	21.95
$^{cis}\mathbf{II_d}$	-2608.3824126	-18.11	-2610.0263167	-16.99
$^{trans}\mathbf{II_d}$	-2608.3799801	-16.58	-2610.0263630	-17.02
$^{cis}\mathbf{II_q}$	-2608.3924098	-24.38	-2610.0355149	-22.76
$^{trans}\mathbf{II_q}$	-2608.3861341	-20.44	-2610.0319371	-20.52
$^{cis}\mathbf{II_s}$	-2608.4032289	-31.17	-2610.0496030	-31.60
$^{trans}\mathbf{II_s}$	-2608.3945441	-25.72	-2610.0413027	-26.40
$^{cis}\mathbf{TS(II\text{-}III)_s}$	-2608.3980640	-27.93	-2610.0452552	-28.88
$^{cis}\mathbf{III_d}^{b,c}$	-2608.4515689	-61.50	-2610.0906898	-57.39
$^{trans}\mathbf{III_d}^{b}$	-2608.4518821	-61.70	-2610.0938960	-59.40
$^{cis}\mathbf{III_q}^{b,c}$	-2608.4527559	-62.25	-2610.0909999	-57.58
$^{trans}\mathbf{III_q}^{b}$	-2608.4526164	-62.16	-2610.0942252	-59.61
$^{trans}\mathbf{III_{q,IRC}}^{c}$	-2608.4430746	-56.17	-2610.0884598	-55.99
$^{cis}\mathbf{III_s}^{b,c}$	-2608.4647766	-69.79	-2610.1044353	-66.01
$^{trans}\mathbf{III_s}^{b}$	-2608.4597321	-66.62	-2610.1024277	-64.75

[a] After several attempts we could not find the optimized geometry of this TS due to its very low imaginary frequency. The data presented correspond to geometry of the maximum of a relaxed scan connecting $^{cis}\mathbf{I_q}$ and $^{cis}\mathbf{II_q}$. [b] Minimum Gibbs energy conformer. [c] IRC conformer.

Table S7. Absolute and relative Gibbs energies computed at UB3LYP-D3BJ/Def2TZVP /SMD(acetonitrile)//UB3LYP-D3BJ/Def2SVP/SMD(acetonitrile) level including thermal quasiharmonic corrections of the enthalpic and entropic corrections calculated at 233.15 K using Grimme's algorithm of Goodvibes code.[18]

	**Gibbs energy (a.u)**	**Relative Gibbs energy (kcal·mol^{-1})**
$^{cis}\mathbf{I}_d$[b]	-2609.4044539	0.00
$^{trans}\mathbf{I}_d$[b,c]	-2609.4218315	0.36
$^{cis}\mathbf{I}_q$[b]	-2609.4014751	1.87
$^{cis}\mathbf{I}_{q,IRC}$[c]	-2609.4192113	2.28
$^{trans}\mathbf{I}_q$[b,c]	-2609.4210962	1.15
$^{cis}\mathbf{I}_s$[b]	-2609.3828821	13.54
$^{trans}\mathbf{I}_s$[b]	-2609.3885820	9.96
$^{cis}\mathbf{TS(I\text{-}III)}_q$	-2609.3818208	14.20
$^{trans}\mathbf{TS(I\text{-}III)}_q$	-2609.3830645	13.42
$^{cis}\mathbf{TS(I\text{-}III)}_d$[a]	-2609.3672784	23.33
$^{trans}\mathbf{TS(I\text{-}III)}_d$	-2609.3698802	21.70
$^{cis}\mathbf{II}_d$	-2609.4346432	-18.94
$^{trans}\mathbf{II}_d$	-2609.4367089	-20.24
$^{cis}\mathbf{II}_q$	-2609.4478251	-27.22
$^{trans}\mathbf{II}_q$	-2609.4449560	-25.42
$^{cis}\mathbf{II}_s$	-2609.4607791	-35.34
$^{trans}\mathbf{II}_s$	-2609.4573737	-33.21
$^{cis}\mathbf{TS(II\text{-}III)}_s$	-2609.4590815	-34.28
$^{cis}\mathbf{III}_d$[b,c]	-2609.5072569	-53.56
$^{trans}\mathbf{III}_d$[b]	-2609.4969408	-58.04
$^{cis}\mathbf{III}_q$[b,c]	-2609.5121420	-56.20
$^{trans}\mathbf{III}_q$[b]	-2609.5008588	-60.50
$^{trans}\mathbf{III}_{q,IRC}$[c]	-2609.5146982	-57.50
$^{cis}\mathbf{III}_s$[b,c]	-2609.5292788	-66.71
$^{trans}\mathbf{III}_s$[b]	-2609.5123905	-67.73

[a] After several attempts we could not find the optimized geometry of this TS due to its very low imaginary frequency. The data presented correspond to geometry of the maximum of a relaxed scan connecting $^{cis}\mathbf{I}_q$ and $^{cis}\mathbf{II}_q$. [b] Minimum Gibbs energy conformer. [c] IRC conformer.

Table S8. Mulliken charges of the carbons (including the charge of their bonded hydrogens) in the carbon chain computed at UB3LYP-D3BJ/Def2SVP/SMD(acetonitrile) level of theory.

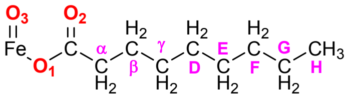

compound **I**

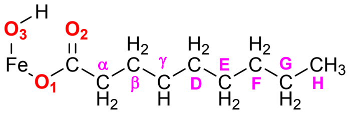

compound **II**

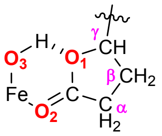

compound ^{trans}**III**

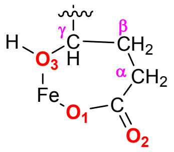

compound ^{cis}**III**

	α	β	γ	D	E	F	G	H	sum
$^{cis}\mathbf{I}_{d}^{a}$	0.191	0.050	0.008	0.010	0.018	-0.013	-0.023	0.029	0.270
$^{trans}\mathbf{I}_{d}^{a,b}$	0.203	0.033	0.003	0.019	0.018	-0.016	-0.022	0.028	0.265
$^{cis}\mathbf{I}_{q}^{a}$	0.155	0.030	0.006	0.008	0.017	-0.013	-0.023	0.029	0.209
$^{cis}\mathbf{I}_{q,IRC}^{b}$	0.143	0.033	-0.009	0.017	0.016	-0.016	-0.023	0.028	0.189
$^{trans}\mathbf{I}_{q}^{a,b}$	0.141	0.028	-0.005	0.019	0.016	-0.016	-0.023	0.028	0.187
$^{cis}\mathbf{I}_{s}^{a}$	0.212	0.045	0.019	0.011	0.022	-0.013	-0.022	0.029	0.302
$^{trans}\mathbf{I}_{s}^{a}$	0.226	0.058	0.025	0.015	0.033	-0.024	-0.023	0.030	0.341
$^{cis}\mathbf{II}_{d}$	0.174	0.244	0.145	0.220	0.108	0.017	0.008	0.066	0.983
$^{trans}\mathbf{II}_{d}$	0.153	0.273	0.142	0.262	0.109	0.027	0.019	0.030	1.016
$^{cis}\mathbf{IRC1}_{q}$	0.149	0.118	-0.172	0.094	0.049	-0.011	-0.022	0.032	0.237
$^{cis}\mathbf{IRC2}_{q}$	0.181	0.259	0.106	0.194	0.128	0.012	-0.008	0.043	0.915
$^{cis}\mathbf{II}_{q}$	0.136	0.277	0.137	0.206	0.134	0.009	-0.002	0.033	0.930
$^{trans}\mathbf{IRC1}_{q}$	0.140	0.117	-0.182	0.082	0.052	-0.014	-0.022	0.032	0.205
$^{trans}\mathbf{IRC2}_{q}$	0.181	0.265	0.122	0.188	0.133	0.017	-0.008	0.044	0.943
$^{trans}\mathbf{II}_{q}$	0.152	0.282	0.142	0.264	0.113	0.028	0.017	0.038	1.035
$^{cis}\mathbf{II}_{s}$	0.194	0.265	0.178	0.239	0.105	0.016	-0.025	0.045	1.016
$^{trans}\mathbf{II}_{s}$	0.151	0.279	0.144	0.265	0.112	0.029	0.017	0.039	1.038
$^{cis}\mathbf{III}_{d}^{a,b}$	0.141	0.077	0.153	0.036	0.067	-0.011	-0.015	0.036	0.486
$^{trans}\mathbf{III}_{d}^{a}$	0.210	0.133	0.135	0.103	0.043	-0.008	-0.017	0.033	0.632
$^{cis}\mathbf{III}_{q}^{a,b}$	0.139	0.088	0.156	0.036	0.060	-0.011	-0.018	0.036	0.486
$^{trans}\mathbf{III}_{q}^{a}$	0.198	0.137	0.124	0.101	0.044	-0.009	-0.017	0.033	0.612
$^{trans}\mathbf{III}_{q,IRC}^{b}$	0.167	0.121	0.110	0.072	0.046	-0.007	-0.025	0.033	0.517
$^{cis}\mathbf{III}_{s}^{a,b}$	0.131	0.092	0.154	0.037	0.066	-0.009	-0.018	0.037	0.488
$^{trans}\mathbf{III}_{s}^{a}$	0.198	0.137	0.124	0.101	0.044	-0.009	-0.017	0.033	0.612

[a] Minimum Gibbs energy conformer. [b] IRC conformer.

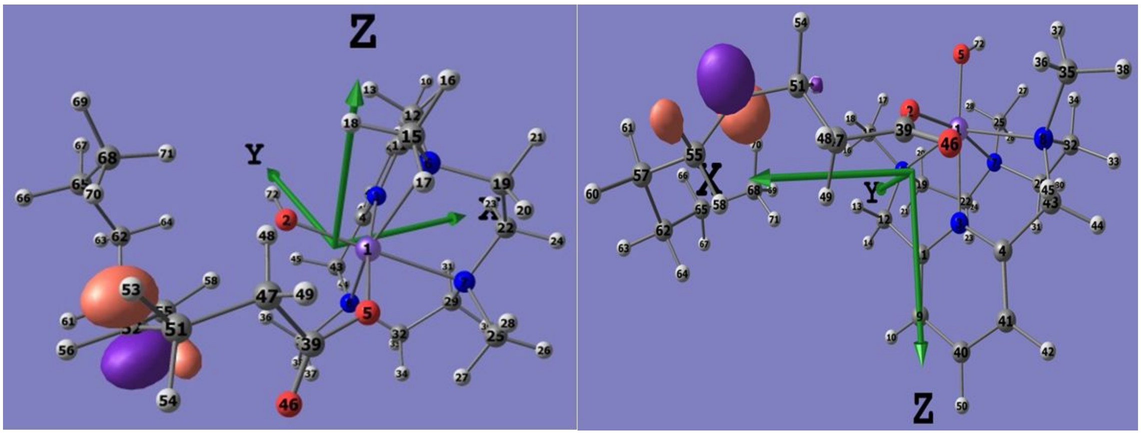

Figure S56. LUMO of $^{cis}\mathbf{II}_s$ (left panel) and $^{trans}\mathbf{II}_s$ (right panel) which corresponds to the empty p orbital perpendicular to the sp^2 plane of the Cγ. The isosurfaces of the molecular orbitals are calculated using a contour value of 0.12.

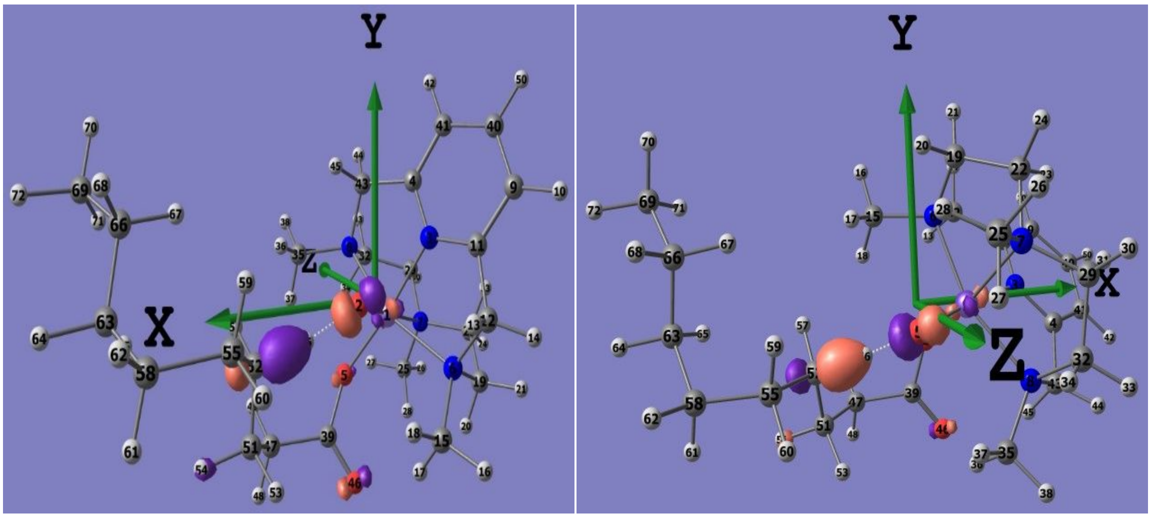

Figure S57. The HOMO of $^{cis}\mathbf{TS(I\text{-}III)}_q$ and $^{trans}\mathbf{TS(I\text{-}III)}_q$ are shown in the left and right panels, respectively. The isosurfaces of the molecular orbitals are calculated using contour value of 0.12.

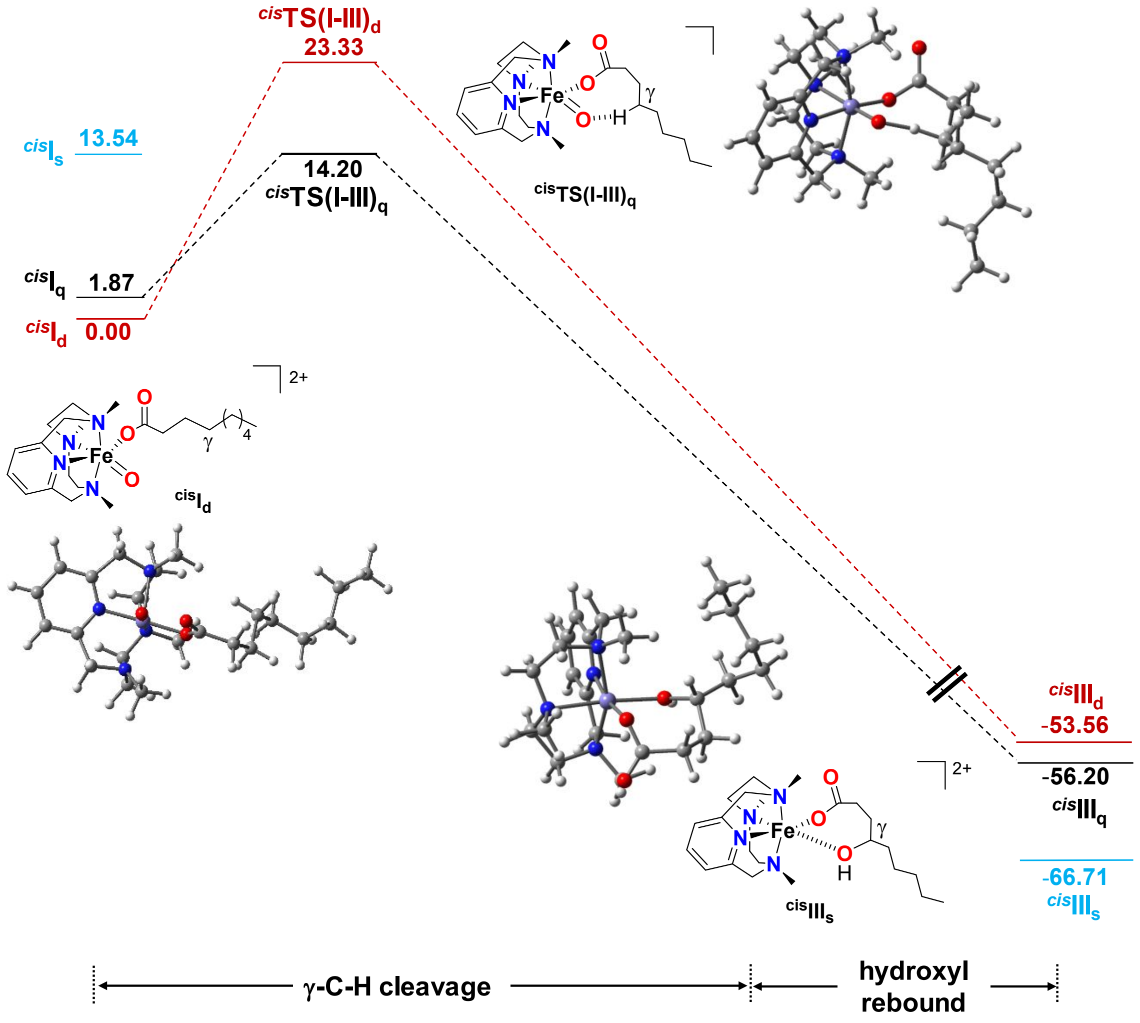

Figure S58. Reaction profile for the γ-C−H lactonization of pernonanoic acid computed at the B3LYP-D3BJ/Def2TZVP/SMD(acetonitrile)//B3LYP-D3BJ/Def2SVP/SMD(acetonitrile) level of theory for compound **^{cis}2d**. Gibbs energies are given in kcal·mol^{-1}. Subscripts d, q, and s represent spin states S = 1/2, S = 3/2 and S = 5/2, respectively.

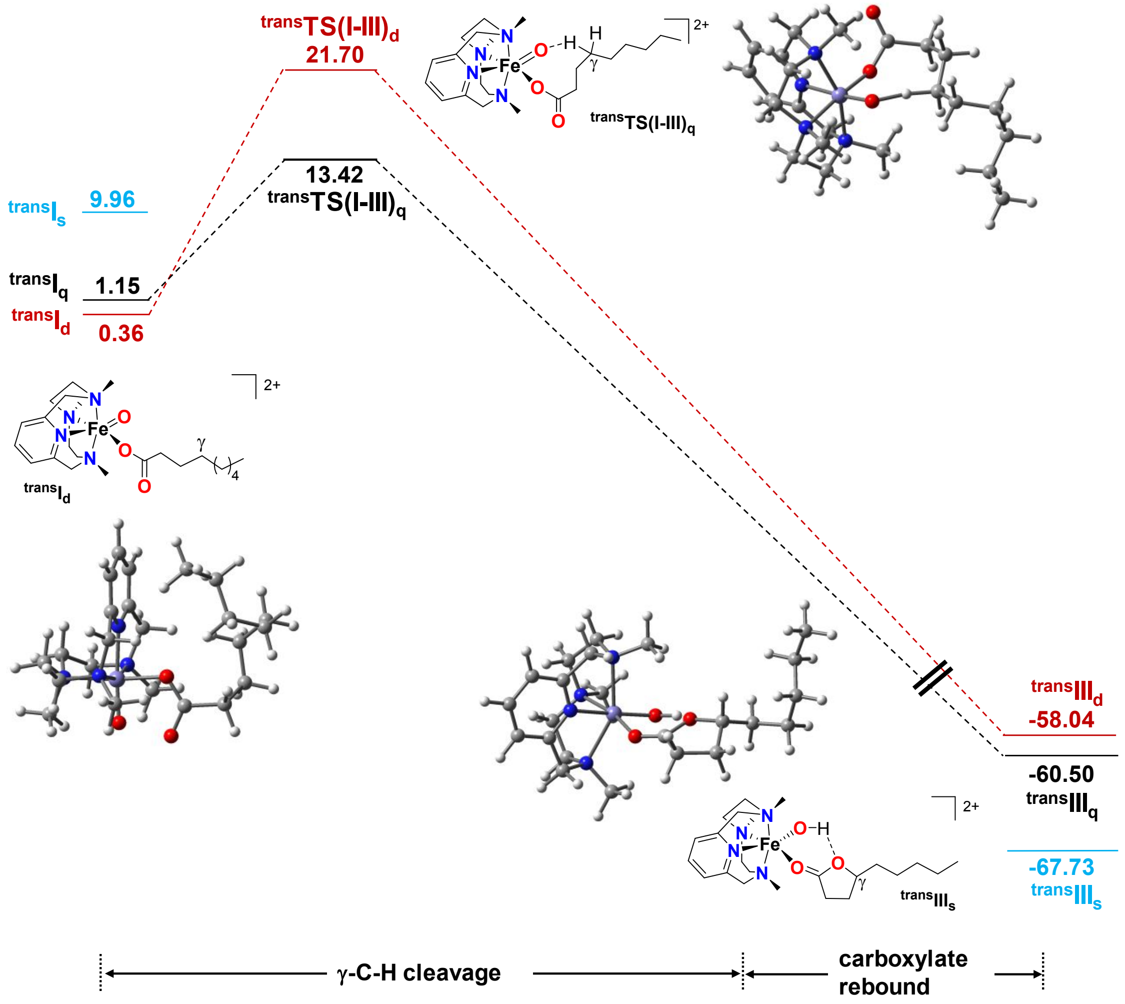

Figure S59. Reaction profile for the γ-C−H lactonization of pernonanoic acid computed at the B3LYP-D3BJ/Def2TZVP/SMD(acetonitrile)//B3LYP-D3BJ/Def2SVP/SMD (acetonitrile) level of theory for compound **^{trans}2d**. Gibbs energies are given in kcal·mol^{-1}. Subscripts d, q and s represent spin states S = 1/2, S = 3/2 and S = 5/2, respectively.

9.5. Cartesian coordinates of the optimized geometries of ^{cis}I$_d$, ^{trans}I$_d$, ^{cis}I$_q$, ^{cis}I$_{q,IRC}$, ^{trans}I$_q$, ^{cis}I$_s$, ^{trans}I$_s$, ^{cis}TS(I-III)$_q$, ^{trans}TS(I-III)$_q$, ^{cis}TS(I-III)$_d$, ^{trans}TS(I-III)$_d$, ^{cis}II$_d$, ^{trans}II$_d$, ^{cis}IRC1$_q$, ^{cis}IRC2$_q$, ^{cis}II$_q$, ^{trans}IRC1$_q$, ^{trans}IRC2$_q$, ^{trans}II$_q$, ^{cis}II$_s$, ^{trans}II$_s$, ^{cis}TS(II-III)$_s$,^{cis}III$_d$, ^{trans}III$_d$, ^{cis}III$_q$, ^{trans}III$_q$, ^{trans}III$_{q,IRC}$, ^{cis}III$_s$, and ^{trans}III$_s$

Optimized geometry of ^{cis}I$_d$ [UB3LYP/def2SVP/GD3BJ/SMD (acetonitrile)]
Electronic energy: -2608.35355940 a.u.
Lowest frequency: 14.63 cm^{-1}

Atomic number	x	y	z
26	1.292544000	-0.167389000	-0.361603000
8	1.448686000	-0.371846000	-1.977438000
7	3.106883000	0.432982000	-0.226715000
6	4.066085000	-0.485212000	-0.031253000
8	-0.511173000	-0.800597000	-0.431706000
7	0.894374000	1.870428000	-0.443090000
7	1.016766000	0.100735000	1.736770000
7	2.117558000	-1.984155000	0.203146000
6	4.661704000	2.210455000	-0.266528000
1	4.874076000	3.272394000	-0.394201000
6	3.360625000	1.738383000	-0.405526000
6	2.172144000	2.512480000	-0.882283000
1	2.197231000	2.495547000	-1.982953000
1	2.198093000	3.564280000	-0.564413000
6	-0.182037000	2.221039000	-1.396891000
1	-0.292991000	3.316109000	-1.440478000
1	-1.126330000	1.782108000	-1.057196000
1	0.072411000	1.835966000	-2.390581000
6	0.522851000	2.333073000	0.934515000
1	-0.559238000	2.196462000	1.041452000
1	0.731068000	3.409141000	1.024200000
6	1.269385000	1.541866000	1.985995000
1	2.349284000	1.729672000	1.950051000
1	0.922583000	1.825367000	2.992232000
6	-0.349613000	-0.242776000	2.213168000
1	-0.396343000	-0.061073000	3.298100000
1	-0.581087000	-1.289875000	2.007095000
1	-1.100018000	0.368228000	1.707429000
6	2.004663000	-0.800709000	2.380748000
1	1.779403000	-0.916520000	3.452825000
1	3.002552000	-0.351856000	2.303998000
6	1.934553000	-2.142154000	1.684292000
1	2.698096000	-2.831462000	2.073561000
1	0.957795000	-2.612321000	1.844705000
6	1.512609000	-3.136117000	-0.503405000
1	1.611777000	-2.990646000	-1.585215000
1	0.453584000	-3.210983000	-0.232594000
1	2.028133000	-4.063116000	-0.206252000
6	-0.993694000	-1.081649000	-1.573090000
6	5.674613000	1.289291000	0.019749000
6	5.387165000	-0.075997000	0.118237000
1	6.170809000	-0.813694000	0.293191000
6	3.571518000	-1.893847000	-0.137839000
1	4.152558000	-2.593120000	0.479815000
1	3.692126000	-2.200947000	-1.188336000
8	-0.354560000	-0.954345000	-2.645629000
6	-2.401435000	-1.640594000	-1.649078000

1	-2.267515000	-2.712382000	-1.877090000
1	-2.891115000	-1.194928000	-2.528835000
1	6.702645000	1.636063000	0.142962000
6	-3.224044000	-1.450741000	-0.376613000
6	-3.592844000	0.004746000	-0.085387000
1	-2.672249000	-1.874479000	0.478116000
1	-4.137407000	-2.055655000	-0.490036000
6	-4.293970000	0.210526000	1.261344000
1	-2.677386000	0.617676000	-0.094606000
1	-4.222849000	0.388595000	-0.906358000
6	-5.610397000	-0.558566000	1.443077000
1	-4.474937000	1.289758000	1.401308000
1	-3.601260000	-0.085026000	2.069740000
1	-5.399612000	-1.641184000	1.481830000
1	-6.030236000	-0.299989000	2.431745000
6	-6.674103000	-0.303213000	0.368891000
1	-7.558061000	-0.926528000	0.594535000
1	-6.307018000	-0.652801000	-0.612933000
6	-7.119441000	1.155547000	0.243924000
1	-6.256668000	1.786448000	-0.031152000
1	-7.456104000	1.516975000	1.233082000
6	-8.233768000	1.354683000	-0.781359000
1	-8.535408000	2.412469000	-0.854996000
1	-7.913509000	1.029187000	-1.786024000
1	-9.131120000	0.769548000	-0.516780000

Optimized geometry of $^{trans}\mathbf{I_d}$ [UB3LYP/def2SVP/GD3BJ/SMD (acetonitrile)]
Electronic energy: -2608.35330194 a.u.
Lowest frequency: 15.64 cm^{-1}

Atomic number	x	y	z
26	1.444768000	-0.501724000	0.224238000
8	-0.115344000	-0.249744000	-0.877935000
7	2.012273000	1.246597000	-0.529545000
6	2.698766000	1.257826000	-1.681721000
8	0.879734000	-1.929294000	0.776644000
7	0.718676000	0.744038000	1.717431000
7	3.145445000	-0.637605000	1.353693000
7	2.607330000	-1.189033000	-1.353674000
6	2.053566000	3.609776000	-0.380171000
1	1.756150000	4.524072000	0.134540000
6	1.628217000	2.371347000	0.091046000
6	0.626161000	2.135658000	1.177033000
1	-0.369108000	2.253527000	0.722219000
1	0.704116000	2.865309000	1.995513000
6	-0.609841000	0.326115000	2.222031000
1	-0.911486000	0.987200000	3.049911000
1	-0.552894000	-0.708519000	2.576752000
1	-1.348252000	0.405034000	1.417211000
6	1.723527000	0.671892000	2.827224000
1	1.463298000	-0.194013000	3.446350000
1	1.638047000	1.568010000	3.459087000
6	3.123285000	0.533901000	2.271967000
1	3.433448000	1.426450000	1.716102000
1	3.847621000	0.384030000	3.086995000
6	3.198727000	-1.891664000	2.155703000
1	4.146848000	-1.906151000	2.713953000
1	3.143522000	-2.767451000	1.503612000
1	2.361831000	-1.930964000	2.858463000
6	4.277746000	-0.618676000	0.387595000
1	5.200438000	-0.950298000	0.888010000
1	4.438051000	0.411663000	0.049990000
6	3.936743000	-1.545674000	-0.757380000
1	4.710031000	-1.507646000	-1.538283000
1	3.870810000	-2.583027000	-0.411006000
6	2.033539000	-2.377710000	-2.025945000
1	1.076048000	-2.114288000	-2.489820000
1	1.885176000	-3.173542000	-1.288318000
1	2.724817000	-2.723319000	-2.810851000
6	-0.993419000	-1.175025000	-0.859181000
6	2.848719000	3.642090000	-1.530071000
6	3.158640000	2.459847000	-2.210160000
1	3.732004000	2.467842000	-3.137658000
6	2.757972000	-0.081393000	-2.346535000
1	3.674598000	-0.223802000	-2.936439000
1	1.904646000	-0.130417000	-3.040176000
8	-0.889504000	-2.217687000	-0.173734000
6	-2.214219000	-1.002107000	-1.740921000
1	-1.971999000	-1.535157000	-2.676983000
1	-2.283129000	0.068563000	-1.982621000
1	3.207191000	4.599026000	-1.914882000
6	-3.514277000	-1.533471000	-1.136932000
6	-3.914705000	-0.822873000	0.157414000
1	-3.419898000	-2.616878000	-0.954122000
1	-4.300522000	-1.411600000	-1.898171000
6	-5.225313000	-1.325480000	0.772329000

1	-3.111629000	-0.953515000	0.901970000
1	-3.977122000	0.262980000	-0.032428000
6	-6.474336000	-1.158594000	-0.105482000
1	-5.380448000	-0.806349000	1.733297000
1	-5.113814000	-2.395212000	1.024348000
1	-6.401565000	-1.827469000	-0.980312000
1	-7.350152000	-1.511576000	0.467997000
6	-6.744954000	0.267527000	-0.599027000
1	-7.652436000	0.255057000	-1.229187000
1	-5.926162000	0.595375000	-1.264772000
6	-6.930392000	1.304826000	0.510807000
1	-6.012705000	1.366227000	1.120900000
1	-7.727490000	0.962751000	1.196292000
6	-7.272675000	2.694949000	-0.021130000
1	-7.398083000	3.424337000	0.795951000
1	-6.477852000	3.073923000	-0.686401000
1	-8.209941000	2.681951000	-0.603326000

Optimized geometry of $^{cis}I_q$ [UB3LYP/def2SVP/GD3BJ/SMD (acetonitrile)]
Electronic energy: -2608.34683412 a.u.
Lowest frequency: 16.09 cm^{-1}

Atomic number	x	y	z
26	1.284822000	-0.165694000	-0.414337000
8	1.624715000	-0.333262000	-2.031052000
7	3.153173000	0.380904000	-0.168443000
6	4.074549000	-0.567971000	0.057003000
8	-0.445608000	-0.712384000	-0.535274000
7	1.019384000	1.888149000	-0.484684000
7	0.939707000	0.083066000	1.686216000
7	2.071098000	-2.000890000	0.141481000
6	4.773783000	2.102799000	-0.123008000
1	5.025759000	3.157934000	-0.233683000
6	3.465909000	1.676496000	-0.327609000
6	2.335187000	2.493045000	-0.861412000
1	2.409754000	2.479871000	-1.959609000
1	2.370049000	3.541378000	-0.533872000
6	-0.004685000	2.274412000	-1.485029000
1	-0.058788000	3.373622000	-1.529817000
1	-0.977461000	1.877157000	-1.178871000
1	0.271446000	1.871207000	-2.464969000
6	0.596496000	2.340728000	0.885043000
1	-0.493466000	2.244232000	0.933483000
1	0.842976000	3.406176000	0.995140000
6	1.255316000	1.506968000	1.959679000
1	2.342868000	1.642378000	1.977334000
1	0.874914000	1.802854000	2.949918000
6	-0.452063000	-0.200936000	2.119074000
1	-0.524680000	-0.016602000	3.202201000
1	-0.716363000	-1.238765000	1.906952000
1	-1.162159000	0.438253000	1.590300000
6	1.863173000	-0.874842000	2.344025000
1	1.583236000	-1.018111000	3.399522000
1	2.881089000	-0.467041000	2.331869000
6	1.772856000	-2.188632000	1.602388000
1	2.474010000	-2.930122000	2.011520000
1	0.764088000	-2.609562000	1.677066000
6	1.471035000	-3.104865000	-0.644963000
1	1.633725000	-2.922638000	-1.713606000
1	0.398463000	-3.158766000	-0.431893000
1	1.950208000	-4.052621000	-0.352856000
6	-1.106456000	-0.979837000	-1.666253000
6	5.740510000	1.147142000	0.204302000
6	5.400703000	-0.206815000	0.273286000
1	6.147346000	-0.975403000	0.474955000
6	3.547606000	-1.956420000	-0.101073000
1	4.053941000	-2.683250000	0.549120000
1	3.728326000	-2.258390000	-1.144130000
8	-0.707877000	-0.670762000	-2.770116000
6	-2.402611000	-1.735354000	-1.441646000
1	-2.116430000	-2.802870000	-1.432087000
1	-3.018011000	-1.580051000	-2.340414000
1	6.772553000	1.457236000	0.379781000
6	-3.170842000	-1.388786000	-0.166848000
6	-3.607232000	0.075411000	-0.095269000
1	-2.554852000	-1.635678000	0.711587000
1	-4.052572000	-2.047454000	-0.120993000
6	-4.275534000	0.466769000	1.226768000

1	-2.725049000	0.722177000	-0.239854000
1	-4.280975000	0.294269000	-0.941919000
6	-5.543509000	-0.322510000	1.583346000
1	-4.508907000	1.544727000	1.196799000
1	-3.543486000	0.341069000	2.044519000
1	-5.274282000	-1.371543000	1.795406000
1	-5.948499000	0.079434000	2.529468000
6	-6.649262000	-0.304882000	0.521501000
1	-7.492152000	-0.921993000	0.881504000
1	-6.291602000	-0.799168000	-0.399704000
6	-7.175134000	1.087976000	0.167627000
1	-6.355317000	1.703080000	-0.241781000
1	-7.503264000	1.596827000	1.092716000
6	-8.326293000	1.054912000	-0.835519000
1	-8.685492000	2.068784000	-1.077135000
1	-8.016961000	0.577361000	-1.781081000
1	-9.183749000	0.481867000	-0.443062000

Optimized geometry of $^{cis}\mathbf{I}_{q,IRC}$ [UB3LYP/def2SVP/GD3BJ/SMD (acetonitrile)]
Electronic energy: -2608.34607264 a.u.
Lowest frequency: 8.99 cm^{-1}

Atomic Number	x	y	z
26	-1.306047000	-0.233735000	-0.082989000
8	-0.881598000	-0.357780000	-1.676417000
7	-2.948411000	0.669526000	-0.664889000
6	-2.927350000	2.001692000	-0.820780000
8	0.182554000	-1.020701000	0.581011000
7	-2.580494000	-1.866247000	-0.076826000
7	-1.921363000	-0.022880000	1.964351000
7	-0.665498000	1.727813000	0.121312000
6	-5.208389000	0.526334000	-1.346417000
1	-6.082077000	-0.083079000	-1.579506000
6	-4.015996000	-0.083781000	-0.972775000
6	-3.713080000	-1.545762000	-1.000051000
1	-3.394788000	-1.797249000	-2.023411000
1	-4.583242000	-2.170162000	-0.754300000
6	-1.893411000	-3.095270000	-0.542178000
1	-2.623745000	-3.919525000	-0.570352000
1	-1.089091000	-3.346023000	0.156872000
1	-1.480287000	-2.926144000	-1.542091000
6	-3.075825000	-2.056563000	1.330550000
1	-2.348451000	-2.696344000	1.841982000
1	-4.030832000	-2.599845000	1.297171000
6	-3.223353000	-0.730461000	2.041013000
1	-4.003714000	-0.107612000	1.588205000
1	-3.510059000	-0.898556000	3.091024000
6	-0.985729000	-0.604370000	2.960837000
1	-1.418158000	-0.464751000	3.963868000
1	-0.013290000	-0.109137000	2.911052000
1	-0.826212000	-1.668884000	2.773768000
6	-2.013368000	1.441945000	2.184835000
1	-2.060640000	1.668094000	3.261639000
1	-2.936520000	1.823420000	1.732989000
6	-0.783758000	2.077374000	1.576681000
1	-0.804103000	3.172072000	1.676279000
1	0.124676000	1.714584000	2.070364000
6	0.738256000	1.886747000	-0.329784000
1	0.823704000	1.562605000	-1.373269000
1	1.395313000	1.285702000	0.306652000
1	1.021291000	2.947844000	-0.246325000
6	1.193791000	-1.552157000	-0.120756000
6	-5.243553000	1.921840000	-1.427862000
6	-4.089550000	2.672934000	-1.188050000
1	-4.078979000	3.757772000	-1.297667000
6	-1.559666000	2.591511000	-0.712193000
1	-1.565518000	3.616333000	-0.315623000
1	-1.139182000	2.629415000	-1.728628000
8	1.100003000	-1.907310000	-1.274799000
6	2.445299000	-1.705974000	0.719767000
1	2.307387000	-2.643973000	1.287326000
1	2.468366000	-0.897075000	1.466033000
1	-6.170767000	2.426189000	-1.707235000
6	3.728259000	-1.750141000	-0.106628000
6	4.047008000	-0.416638000	-0.786845000
1	3.640067000	-2.543132000	-0.867854000

1	4.554162000	-2.042704000	0.560663000
6	5.282117000	-0.450950000	-1.693267000
1	3.178830000	-0.105786000	-1.392164000
1	4.168398000	0.360769000	-0.011915000
6	6.598806000	-0.828364000	-0.999056000
1	5.390977000	0.535207000	-2.175976000
1	5.101462000	-1.167906000	-2.514144000
1	6.553245000	-1.881962000	-0.673822000
1	7.411473000	-0.781086000	-1.746049000
6	6.979947000	0.041034000	0.204996000
1	7.926307000	-0.340683000	0.628730000
1	6.227557000	-0.076503000	1.005798000
6	7.146373000	1.529844000	-0.107950000
1	6.193445000	1.940905000	-0.483572000
1	7.872865000	1.646884000	-0.933061000
6	7.604344000	2.347116000	1.098231000
1	7.714591000	3.415235000	0.848589000
1	6.880903000	2.272337000	1.928277000
1	8.577497000	1.991086000	1.477471000

Optimized geometry of ${}^{trans}\mathbf{I_q}$
[UB3LYP/def2SVP/GD3BJ/SMD (acetonitrile)]
Electronic energy: -2608.34733427 a.u.
Lowest frequency: 8.92 cm^{-1}

Atomic Number	x	y	z
26	1.400852000	-0.435322000	0.366475000
8	0.039463000	-0.471020000	-0.862887000
7	2.082931000	1.011577000	-0.801028000
6	2.856774000	0.675586000	-1.841063000
8	0.807544000	-1.624833000	1.339237000
7	0.600623000	1.193178000	1.391558000
7	3.038052000	-0.181380000	1.645925000
7	2.733767000	-1.555863000	-0.775425000
6	2.150564000	3.306639000	-1.364084000
1	1.829760000	4.331695000	-1.175565000
6	1.668313000	2.264148000	-0.577425000
6	0.576375000	2.355396000	0.442567000
1	-0.378106000	2.321525000	-0.103646000
1	0.600682000	3.296148000	1.009823000
6	-0.771895000	0.917910000	1.878332000
1	-1.143878000	1.802888000	2.418679000
1	-0.750784000	0.055716000	2.553881000
1	-1.430267000	0.710550000	1.028285000
6	1.509841000	1.466474000	2.554372000
1	1.202354000	0.807436000	3.373954000
1	1.363679000	2.503516000	2.888881000
6	2.944445000	1.210546000	2.165038000
1	3.291385000	1.903656000	1.390094000
1	3.608983000	1.325311000	3.034724000
6	3.010253000	-1.140092000	2.782928000
1	3.904228000	-0.966633000	3.401115000
1	3.010189000	-2.170997000	2.419611000
1	2.112782000	-0.986462000	3.388338000
6	4.249499000	-0.413379000	0.813973000
1	5.128305000	-0.534425000	1.465673000
1	4.422524000	0.467091000	0.184628000
6	4.020438000	-1.660288000	-0.003129000
1	4.846586000	-1.832403000	-0.707233000
1	3.943436000	-2.542953000	0.640935000
6	2.221513000	-2.915498000	-1.071536000
1	1.284771000	-2.845488000	-1.632805000
1	2.049839000	-3.447971000	-0.129817000
1	2.973188000	-3.452557000	-1.671833000
6	-0.978244000	-1.339284000	-0.914281000
6	3.032161000	2.995164000	-2.403980000
6	3.375475000	1.665017000	-2.670253000
1	4.019051000	1.398127000	-3.509087000
6	2.939447000	-0.803832000	-2.055886000
1	3.883363000	-1.118369000	-2.522637000
1	2.119826000	-1.078765000	-2.736194000
8	-0.966535000	-2.433687000	-0.395896000
6	-2.130275000	-0.812672000	-1.751462000
1	-1.904592000	-1.112044000	-2.790698000
1	-2.111885000	0.287269000	-1.733799000
1	3.434405000	3.794209000	-3.030166000
6	-3.485327000	-1.363944000	-1.311157000
6	-3.911445000	-0.867710000	0.072469000
1	-3.442057000	-2.465776000	-1.312632000
1	-4.233405000	-1.074593000	-2.065964000

6	-5.225896000	-1.469650000	0.580537000
1	-3.115387000	-1.103354000	0.799140000
1	-3.982988000	0.234088000	0.051759000
6	-6.456172000	-1.201566000	-0.298589000
1	-5.413795000	-1.092701000	1.600345000
1	-5.100569000	-2.562442000	0.683063000
1	-6.342976000	-1.732294000	-1.259549000
1	-7.337723000	-1.653640000	0.190466000
6	-6.750440000	0.275629000	-0.584896000
1	-7.637662000	0.336523000	-1.240629000
1	-5.921068000	0.717798000	-1.166046000
6	-6.998645000	1.130804000	0.659652000
1	-6.100667000	1.124728000	1.301378000
1	-7.803696000	0.669373000	1.260691000
6	-7.369090000	2.575152000	0.329144000
1	-7.540894000	3.170468000	1.240970000
1	-6.567998000	3.070668000	-0.245873000
1	-8.288498000	2.623917000	-0.279074000

Optimized geometry of $^{cis}I_s$ [UB3LYP/def2SVP/GD3BJ/SMD (acetonitrile)]
Electronic energy: -2608.32503883 a.u.
Lowest frequency: 9.45 cm^{-1}

Atomic Number	x	y	z
26	-1.248560000	0.192247000	-0.320726000
8	-1.240678000	0.315315000	-2.004028000
7	-3.171361000	-0.476674000	-0.431124000
6	-4.169373000	0.414418000	-0.344659000
8	0.562098000	0.972960000	-0.253268000
7	-0.919670000	-1.984883000	-0.254423000
7	-1.263420000	0.000601000	1.786762000
7	-2.365812000	2.055574000	0.104867000
6	-4.677076000	-2.299783000	-0.501934000
1	-4.847632000	-3.374715000	-0.573994000
6	-3.379793000	-1.796409000	-0.540227000
6	-2.147845000	-2.622592000	-0.786974000
1	-2.036201000	-2.716950000	-1.878329000
1	-2.267025000	-3.638563000	-0.380637000
6	0.253031000	-2.355821000	-1.073219000
1	0.413879000	-3.446190000	-1.039693000
1	1.145567000	-1.855493000	-0.678337000
1	0.089550000	-2.041437000	-2.111038000
6	-0.688485000	-2.314662000	1.180357000
1	0.378224000	-2.160301000	1.384848000
1	-0.907609000	-3.378019000	1.364717000
6	-1.533674000	-1.438755000	2.079305000
1	-2.603866000	-1.628672000	1.932228000
1	-1.308080000	-1.646723000	3.136799000
6	0.026926000	0.404138000	2.406559000
1	-0.051177000	0.259620000	3.495023000
1	0.245889000	1.453218000	2.190595000
1	0.847556000	-0.204122000	2.015062000
6	-2.363265000	0.878639000	2.285991000
1	-2.257399000	1.015043000	3.373687000
1	-3.317602000	0.367801000	2.112039000
6	-2.318118000	2.219595000	1.584562000
1	-3.153400000	2.853156000	1.922526000
1	-1.387943000	2.748205000	1.825489000
6	-1.732811000	3.205845000	-0.572119000
1	-1.704557000	3.021133000	-1.653108000
1	-0.710099000	3.330873000	-0.196283000
1	-2.302441000	4.128954000	-0.372827000
6	1.213772000	1.232505000	-1.303469000
6	-5.737167000	-1.397935000	-0.371501000
6	-5.489643000	-0.024049000	-0.300606000
1	-6.301709000	0.699029000	-0.213865000
6	-3.746200000	1.855746000	-0.393540000
1	-4.455335000	2.494651000	0.154575000
1	-3.775230000	2.164052000	-1.450087000
8	0.824584000	0.996269000	-2.473142000
6	2.570030000	1.913313000	-1.177146000
1	2.321071000	2.984171000	-1.068627000
1	3.115345000	1.796903000	-2.124205000
1	-6.763675000	-1.768055000	-0.330256000
6	3.374665000	1.405960000	0.018566000
6	3.795577000	-0.058328000	-0.104909000
1	2.786051000	1.554157000	0.938164000
1	4.263021000	2.052605000	0.099915000

6	4.509600000	-0.609360000	1.133527000
1	2.901619000	-0.677683000	-0.292183000
1	4.433553000	-0.175653000	-0.997578000
6	5.797607000	0.125940000	1.531062000
1	4.731246000	-1.676300000	0.962534000
1	3.811194000	-0.580781000	1.989035000
1	5.546071000	1.142988000	1.878175000
1	6.235209000	-0.388905000	2.405078000
6	6.860670000	0.231317000	0.431401000
1	7.722333000	0.796963000	0.829311000
1	6.472153000	0.834661000	-0.408822000
6	7.359719000	-1.111046000	-0.107936000
1	6.519307000	-1.668833000	-0.555879000
1	7.718643000	-1.729128000	0.735558000
6	8.471615000	-0.962121000	-1.144321000
1	8.812565000	-1.941047000	-1.519504000
1	8.130327000	-0.373339000	-2.013047000
1	9.348392000	-0.443709000	-0.719888000

Optimized geometry of ^{trans}**I$_S$** [UB3LYP/def2SVP/GD3BJ/SMD (acetonitrile)]
Electronic energy: -2608.33414040 a.u.
Lowest frequency: 12.57 cm^{-1}

Atomic Number	x	y	z
26	1.470275000	-0.562484000	-0.116714000
8	-0.320244000	-1.239536000	-0.914382000
7	0.434490000	1.131646000	0.178094000
6	0.268378000	1.974535000	-0.860913000
8	2.192835000	-2.021435000	-0.343039000
7	1.142849000	-0.673127000	2.060898000
7	3.344042000	0.435681000	0.470579000
7	1.870340000	0.490064000	-2.035186000
6	-0.743256000	2.588224000	1.637929000
1	-1.139575000	2.798099000	2.632019000
6	-0.067306000	1.394285000	1.398771000
6	0.066620000	0.293302000	2.408739000
1	-0.889409000	-0.250490000	2.422052000
1	0.221432000	0.697566000	3.420685000
6	0.771893000	-2.038436000	2.490209000
1	0.653567000	-2.072961000	3.585990000
1	1.556579000	-2.740246000	2.184695000
1	-0.177912000	-2.326551000	2.022764000
6	2.452674000	-0.283822000	2.653958000
1	3.087975000	-1.178041000	2.656465000
1	2.305743000	0.021492000	3.701909000
6	3.123350000	0.838364000	1.879803000
1	2.513040000	1.750046000	1.893602000
1	4.086857000	1.083718000	2.355383000
6	4.510707000	-0.466235000	0.358384000
1	5.422905000	0.084422000	0.640491000
1	4.610824000	-0.835954000	-0.667224000
1	4.387687000	-1.329096000	1.020610000
6	3.472904000	1.586304000	-0.454208000
1	4.492406000	2.001607000	-0.394518000
1	2.782272000	2.374373000	-0.130898000
6	3.194821000	1.153868000	-1.885101000
1	3.252571000	2.027185000	-2.553814000
1	3.956084000	0.437409000	-2.215912000
6	1.888081000	-0.447669000	-3.176000000
1	0.923295000	-0.965621000	-3.243026000
1	2.685059000	-1.185597000	-3.025901000
1	2.068908000	0.097958000	-4.117411000
6	-0.574010000	-2.453778000	-1.037811000
6	-0.898915000	3.490706000	0.584462000
6	-0.395938000	3.183238000	-0.682999000
1	-0.517641000	3.866121000	-1.524380000
6	0.761994000	1.466977000	-2.184708000
1	1.059428000	2.296400000	-2.844636000
1	-0.081608000	0.949669000	-2.667046000
8	0.208602000	-3.413945000	-0.795464000
6	-1.966449000	-2.892752000	-1.504742000
1	-1.935216000	-3.918908000	-1.893839000
1	-2.163874000	-2.205436000	-2.345489000
1	-1.418361000	4.436500000	0.749786000
6	-3.022010000	-2.752715000	-0.395827000
6	-2.931402000	-1.478949000	0.447626000
1	-2.981275000	-3.637055000	0.260318000
1	-3.991763000	-2.801831000	-0.920832000

6	-4.203933000	-1.150954000	1.232859000
1	-2.093896000	-1.587961000	1.154254000
1	-2.665759000	-0.626020000	-0.194161000
6	-5.379008000	-0.659108000	0.374156000
1	-3.955865000	-0.378999000	1.980357000
1	-4.522889000	-2.037016000	1.809882000
1	-5.768561000	-1.492212000	-0.236601000
1	-6.204340000	-0.370848000	1.049199000
6	-5.055863000	0.517735000	-0.556455000
1	-5.979610000	0.809065000	-1.087507000
1	-4.354492000	0.188588000	-1.344219000
6	-4.474304000	1.751878000	0.138110000
1	-3.528719000	1.487802000	0.641796000
1	-5.163612000	2.077491000	0.938676000
6	-4.216612000	2.910193000	-0.822970000
1	-3.807169000	3.789516000	-0.300382000
1	-3.493619000	2.623875000	-1.605737000
1	-5.144720000	3.224854000	-1.330099000

Optimized geometry of ^{cis}**TS(I-II)$_q$** [UB3LYP/def2SVP/GD3BJ/SMD (acetonitrile)]
Electronic energy: -2608.32639330 a.u.
Lowest frequency: -744.03 cm^{-1}

Atomic Number	x	y	z
26	-0.841244000	-0.242501000	0.099940000
8	0.457527000	0.284443000	-0.921438000
7	-1.759355000	1.357113000	-0.509591000
6	-1.682530000	2.474424000	0.230852000
8	0.111691000	-1.622443000	0.828032000
7	-1.859472000	-1.081084000	-1.519881000
7	-2.553097000	-0.859592000	1.190586000
7	-0.474109000	0.969906000	1.762801000
6	-3.135284000	2.413873000	-2.121605000
1	-3.678655000	2.366752000	-3.065697000
6	-2.410800000	1.311568000	-1.683796000
6	-2.165631000	0.052467000	-2.444529000
1	-1.281948000	0.218157000	-3.078294000
1	-3.004852000	-0.217314000	-3.100379000
6	-1.055537000	-2.086697000	-2.255034000
1	-1.619452000	-2.408992000	-3.145184000
1	-0.866667000	-2.945056000	-1.604263000
1	-0.107063000	-1.633212000	-2.565844000
6	-3.123137000	-1.712993000	-1.008878000
1	-2.884228000	-2.749094000	-0.747301000
1	-3.866419000	-1.737308000	-1.819208000
6	-3.650125000	-0.966064000	0.195236000
1	-3.995303000	0.041385000	-0.064323000
1	-4.507304000	-1.504643000	0.628935000
6	-2.367606000	-2.170636000	1.867045000
1	-3.312501000	-2.441351000	2.363476000
1	-1.568794000	-2.106825000	2.610063000
1	-2.087667000	-2.943378000	1.146553000
6	-2.787976000	0.196488000	2.207693000
1	-3.476443000	-0.169742000	2.985657000
1	-3.269452000	1.057764000	1.728779000
6	-1.457384000	0.563154000	2.820078000
1	-1.564258000	1.384435000	3.543880000
1	-1.025917000	-0.291084000	3.353531000
6	0.901768000	0.829153000	2.292579000
1	1.620426000	1.146598000	1.530457000
1	1.079477000	-0.215807000	2.563677000
1	1.008669000	1.469342000	3.182765000
6	0.725391000	-2.757926000	0.532735000
6	-3.135289000	3.566898000	-1.330300000
6	-2.387978000	3.611405000	-0.150813000
1	-2.336711000	4.512592000	0.460937000
6	-0.693468000	2.387508000	1.343982000
1	-0.975532000	3.001543000	2.210785000
1	0.260802000	2.776315000	0.957521000
8	0.123031000	-3.736312000	0.127621000
6	2.220825000	-2.751182000	0.760125000
1	2.538282000	-3.795964000	0.880847000
1	2.452393000	-2.200888000	1.684958000
1	-3.704847000	4.443200000	-1.646352000
6	2.970051000	-2.119127000	-0.425577000
6	2.854736000	-0.616132000	-0.535569000
1	2.658674000	-2.600123000	-1.368617000
1	4.042970000	-2.358241000	-0.304072000
6	3.547681000	0.041062000	-1.711361000

1	1.690684000	-0.311355000	-0.672578000
1	3.082071000	-0.120797000	0.421280000
6	5.087627000	-0.054390000	-1.662743000
1	3.250587000	1.101573000	-1.749209000
1	3.194309000	-0.418919000	-2.649779000
1	5.388555000	-1.104184000	-1.817689000
1	5.484050000	0.510614000	-2.524561000
6	5.734087000	0.464599000	-0.374371000
1	6.829881000	0.363648000	-0.470419000
1	5.449338000	-0.182555000	0.474742000
6	5.399096000	1.916953000	-0.028231000
1	4.309947000	2.022608000	0.117117000
1	5.654871000	2.563122000	-0.887892000
6	6.121215000	2.413731000	1.222328000
1	5.862009000	3.460000000	1.453209000
1	5.857681000	1.803181000	2.103012000
1	7.216547000	2.360302000	1.100846000

Optimized geometry of ^{trans}**TS(I-II)$_q$** [UB3LYP/def2SVP/GD3BJ/SMD (acetonitrile)]
Electronic energy: -2608.32687576 a.u.
Lowest frequency: -1019.10 cm^{-1}

Atomic Number	x	y	z
26	0.699703000	0.038643000	0.299941000
8	0.062075000	-0.897521000	-1.162830000
7	2.392219000	0.187403000	-0.692727000
6	3.351441000	-0.722439000	-0.469200000
8	-0.736582000	-0.210978000	1.224889000
7	0.289928000	1.812194000	-0.722642000
7	1.403541000	1.316406000	1.785804000
7	1.792313000	-1.428746000	1.311745000
6	3.691159000	1.300240000	-2.326179000
1	3.787740000	2.087478000	-3.074665000
6	2.498703000	1.142545000	-1.627188000
6	1.210294000	1.852087000	-1.902585000
1	0.718182000	1.311699000	-2.724529000
1	1.357479000	2.892563000	-2.225342000
6	-1.112012000	1.882015000	-1.194418000
1	-1.258500000	2.822264000	-1.749382000
1	-1.784312000	1.860647000	-0.329968000
1	-1.316111000	1.029830000	-1.849617000
6	0.556384000	2.952461000	0.211875000
1	-0.360376000	3.125005000	0.787094000
1	0.761863000	3.861097000	-0.372138000
6	1.707989000	2.612695000	1.121261000
1	2.649121000	2.515737000	0.568337000
1	1.849286000	3.394883000	1.882315000
6	0.418556000	1.535332000	2.879262000
1	0.868230000	2.225160000	3.609183000
1	0.174099000	0.590535000	3.370634000
1	-0.505301000	1.965805000	2.485060000
6	2.626648000	0.669978000	2.335616000
1	2.908057000	1.156863000	3.282145000
1	3.453094000	0.819108000	1.631981000
6	2.327023000	-0.788803000	2.560782000
1	3.225746000	-1.334331000	2.882266000
1	1.566981000	-0.915366000	3.339651000
6	0.988953000	-2.612410000	1.706921000
1	0.624802000	-3.128343000	0.814834000
1	0.149512000	-2.283310000	2.328110000
1	1.632025000	-3.296611000	2.283490000
6	-0.273402000	-2.140631000	-1.461445000
6	4.740407000	0.415962000	-2.053422000
6	4.570135000	-0.624194000	-1.134957000
1	5.358386000	-1.353216000	-0.944706000
6	2.905556000	-1.852383000	0.404236000
1	3.724993000	-2.287781000	0.993061000
1	2.495413000	-2.624640000	-0.264614000
8	0.499436000	-3.078737000	-1.361020000
6	-1.702450000	-2.300385000	-1.933934000
1	-1.767727000	-3.243174000	-2.494017000
1	-1.962987000	-1.468020000	-2.605615000
1	5.690739000	0.524135000	-2.580198000
6	-2.679971000	-2.334156000	-0.745468000
6	-2.860360000	-1.022060000	-0.017023000
1	-2.389463000	-3.129563000	-0.037807000
1	-3.670060000	-2.630426000	-1.140372000
6	-3.845311000	-0.999532000	1.132981000

1	-1.804041000	-0.702528000	0.497519000
1	-3.006034000	-0.184234000	-0.714587000
6	-5.321658000	-1.092769000	0.691668000
1	-3.699174000	-0.068521000	1.704528000
1	-3.625322000	-1.830523000	1.824182000
1	-5.513344000	-2.099481000	0.283402000
1	-5.946280000	-1.002048000	1.597604000
6	-5.762717000	-0.045630000	-0.336627000
1	-6.839009000	-0.189378000	-0.539003000
1	-5.252458000	-0.228156000	-1.299509000
6	-5.526776000	1.406849000	0.083619000
1	-4.447650000	1.577258000	0.241923000
1	-6.009079000	1.584890000	1.062201000
6	-6.046374000	2.415504000	-0.938408000
1	-5.861616000	3.453271000	-0.615781000
1	-5.555421000	2.278703000	-1.917225000
1	-7.132518000	2.303429000	-1.096690000

Geometry of **TS(I-II)$_d$** [UB3LYP/def2SVP/GD3BJ/SMD (acetonitrile)]. After several attempts, we could not find the optimized geometry of this TS due to its very low imaginary frequency. The data presented correspond to geometry of the maximum of a relaxed scan connecting ^{cis}**I$_q$** and ^{cis}**II$_q$**.
Electronic energy: -2608.3228924 a.u.
Lowest frequency: -52.07 cm^{-1}

Atomic Number	x	y	z
26	-0.740436000	-0.241294000	0.017487000
8	0.302471000	0.223212000	-1.166508000
7	-1.907220000	1.203960000	-0.571083000
6	-1.828767000	2.390016000	0.053490000
8	0.328054000	-1.505055000	0.784331000
7	-1.931777000	-1.312969000	-1.345802000
7	-2.259516000	-0.848540000	1.413927000
7	-0.249766000	1.148717000	1.478031000
6	-3.596800000	1.987409000	-2.033537000
1	-4.262130000	1.806408000	-2.878160000
6	-2.712939000	0.995894000	-1.625944000
6	-2.455072000	-0.302188000	-2.311521000
1	-1.677268000	-0.125280000	-3.070256000
1	-3.342045000	-0.695060000	-2.827833000
6	-1.182162000	-2.344078000	-2.103337000
1	-1.857413000	-2.800647000	-2.844835000
1	-0.815468000	-3.106851000	-1.411103000
1	-0.339011000	-1.872350000	-2.622379000
6	-3.061284000	-1.949891000	-0.589315000
1	-2.718053000	-2.936864000	-0.261868000
1	-3.908372000	-2.104786000	-1.272824000
6	-3.471153000	-1.107346000	0.599037000
1	-3.917481000	-0.152693000	0.299393000
1	-4.225747000	-1.644799000	1.194660000
6	-1.922783000	-2.080667000	2.177999000
1	-2.778286000	-2.330925000	2.824108000
1	-1.036839000	-1.920000000	2.797057000
1	-1.717851000	-2.913409000	1.500798000
6	-2.399022000	0.285516000	2.360535000
1	-2.930292000	-0.036585000	3.269820000
1	-3.003221000	1.074410000	1.896542000
6	-1.010906000	0.766264000	2.712449000
1	-1.045293000	1.631785000	3.390158000
1	-0.437783000	-0.023515000	3.210588000
6	1.200923000	1.185222000	1.782557000
1	1.756931000	1.466561000	0.881769000
1	1.523834000	0.200474000	2.132067000
1	1.378823000	1.934429000	2.569849000
6	0.976663000	-2.653625000	0.608204000
6	-3.597197000	3.203675000	-1.343811000
6	-2.692707000	3.421357000	-0.302095000
1	-2.638741000	4.377164000	0.219904000
6	-0.675917000	2.496819000	0.992007000
1	-0.880282000	3.157931000	1.845587000
1	0.161087000	2.933487000	0.425850000
8	0.421514000	-3.662741000	0.222403000
6	2.444712000	-2.589135000	0.969434000
1	2.725337000	-3.586663000	1.338831000
1	2.593643000	-1.858378000	1.778211000
1	-4.290813000	3.994504000	-1.636011000
6	3.308723000	-2.208591000	-0.244026000
6	2.993911000	-0.820569000	-0.793252000

1	3.189209000	-2.969022000	-1.034644000
1	4.362837000	-2.251990000	0.077147000
6	3.942282000	-0.311183000	-1.877979000
1	1.958324000	-0.806258000	-1.195605000
1	2.980431000	-0.106062000	0.041750000
6	5.358617000	0.021717000	-1.378330000
1	3.497768000	0.591226000	-2.329999000
1	4.010590000	-1.054525000	-2.691699000
1	5.896162000	-0.912444000	-1.140554000
1	5.917370000	0.489348000	-2.208456000
6	5.411647000	0.941076000	-0.151096000
1	6.469560000	1.140980000	0.095907000
1	4.999066000	0.412106000	0.726882000
6	4.678887000	2.275411000	-0.311156000
1	3.621623000	2.091497000	-0.568295000
1	5.107270000	2.826458000	-1.168197000
6	4.737881000	3.142108000	0.944785000
1	4.208902000	4.099055000	0.804204000
1	4.272132000	2.627785000	1.803124000
1	5.779242000	3.374351000	1.225935000

Optimized geometry of **TS(I-II)$_d$** [UB3LYP/def2SVP/GD3BJ/SMD (acetonitrile)]
Electronic energy: -2608.33053841 a.u.
Lowest frequency: -21.24 cm^{-1}

Atomic Number	x	y	z
26	-0.838133000	-0.438558000	0.256473000
8	0.305205000	0.584496000	-0.821113000
7	-2.369637000	0.480344000	-0.556697000
6	-2.988277000	1.462152000	0.116951000
8	0.475487000	-1.149435000	1.026926000
7	-1.095708000	-1.663290000	-1.405854000
7	-2.076431000	-1.801852000	1.209541000
7	-1.305917000	0.855457000	1.828781000
6	-3.806450000	0.727968000	-2.424980000
1	-4.088818000	0.439077000	-3.437718000
6	-2.710186000	0.135997000	-1.813714000
6	-1.743329000	-0.811106000	-2.447349000
1	-0.956284000	-0.213001000	-2.929688000
1	-2.208926000	-1.443885000	-3.215658000
6	0.198416000	-2.177353000	-1.910433000
1	0.019479000	-2.760182000	-2.827943000
1	0.655012000	-2.821406000	-1.151039000
1	0.858715000	-1.331446000	-2.129347000
6	-1.994182000	-2.803201000	-1.007700000
1	-1.349991000	-3.638736000	-0.715018000
1	-2.580364000	-3.128062000	-1.878891000
6	-2.906710000	-2.403226000	0.130518000
1	-3.665874000	-1.677551000	-0.178399000
1	-3.432746000	-3.285592000	0.526549000
6	-1.304234000	-2.875040000	1.903074000
1	-2.020551000	-3.600185000	2.317082000
1	-0.710897000	-2.449921000	2.717999000
1	-0.635572000	-3.384855000	1.203920000
6	-2.856359000	-1.022978000	2.208618000
1	-3.286702000	-1.706740000	2.955486000
1	-3.689331000	-0.521709000	1.700342000
6	-1.917193000	-0.039388000	2.863013000
1	-2.440259000	0.576157000	3.608749000
1	-1.100587000	-0.557760000	3.378719000
6	-0.115633000	1.525018000	2.407329000
1	0.335198000	2.190252000	1.665599000
1	0.607464000	0.767113000	2.727571000
1	-0.431828000	2.119836000	3.278878000
6	0.490792000	1.885975000	-0.933759000
6	-4.519442000	1.699222000	-1.712147000
6	-4.103046000	2.086460000	-0.437295000
1	-4.616331000	2.872977000	0.116877000
6	-2.305479000	1.879989000	1.375626000
1	-3.015847000	2.093792000	2.186833000
1	-1.760611000	2.802323000	1.138757000
8	-0.322661000	2.736903000	-0.620570000
6	1.877789000	2.205596000	-1.458270000
1	1.855318000	3.231670000	-1.852066000
1	2.127647000	1.514510000	-2.277846000
1	-5.393418000	2.171822000	-2.164865000
6	2.931187000	2.091714000	-0.339458000
6	3.151313000	0.681425000	0.209885000
1	2.657881000	2.773088000	0.485635000
1	3.879424000	2.475164000	-0.750628000
6	4.188900000	0.601579000	1.333248000

1	2.196998000	0.281467000	0.600133000
1	3.430319000	0.007672000	-0.618564000
6	5.609951000	1.045490000	0.953121000
1	4.216857000	-0.436080000	1.706674000
1	3.843564000	1.217499000	2.182902000
1	5.616843000	2.132824000	0.764869000
1	6.266603000	0.888220000	1.827548000
6	6.216594000	0.332364000	-0.260698000
1	7.226007000	0.743698000	-0.441461000
1	5.631574000	0.572398000	-1.166832000
6	6.321379000	-1.188206000	-0.123500000
1	5.314458000	-1.620718000	0.007614000
1	6.878129000	-1.430876000	0.800341000
6	6.999846000	-1.851017000	-1.320544000
1	7.060375000	-2.945166000	-1.200098000
1	6.446942000	-1.648939000	-2.254005000
1	8.027313000	-1.473176000	-1.458877000

Optimized geometry of $^{cis}II_d$ [UB3LYP/def2SVP/GD3BJ/SMD (acetonitrile)]
Electronic energy: -2608.38241255 a.u.
Lowest frequency: 24.98 cm^{-1}

Atomic Number	x	y	z
26	-0.626799000	0.080384000	0.109522000
8	0.503968000	-1.288302000	-0.242694000
7	-2.207251000	-1.043712000	-0.309468000
6	-2.725014000	-1.024155000	-1.549330000
8	0.921787000	1.061341000	0.836894000
7	-1.268240000	-0.489404000	2.180860000
7	-1.970327000	1.821755000	0.545219000
7	-0.985526000	0.698845000	-1.974249000
6	-3.986634000	-2.419445000	0.467426000
1	-4.459864000	-2.960866000	1.287466000
6	-2.797570000	-1.730429000	0.688605000
6	-2.045002000	-1.734998000	1.987875000
1	-1.330701000	-2.571975000	1.950712000
1	-2.721484000	-1.916103000	2.838269000
6	-0.119331000	-0.732777000	3.068888000
1	-0.461891000	-1.038418000	4.072810000
1	0.476425000	0.183469000	3.140868000
1	0.505072000	-1.525572000	2.638113000
6	-2.110089000	0.626201000	2.696632000
1	-1.436631000	1.337039000	3.190682000
1	-2.803982000	0.245825000	3.464632000
6	-2.891256000	1.332988000	1.599039000
1	-3.627937000	0.663028000	1.139100000
1	-3.452514000	2.175249000	2.039184000
6	-1.247296000	3.020623000	1.014009000
1	-1.970252000	3.827438000	1.224608000
1	-0.533515000	3.353564000	0.254739000
1	-0.683063000	2.793396000	1.923698000
6	-2.644152000	2.102484000	-0.745298000
1	-3.111094000	3.101855000	-0.726599000
1	-3.454234000	1.375203000	-0.886964000
6	-1.638543000	2.035325000	-1.882134000
1	-2.129095000	2.275447000	-2.840269000
1	-0.835902000	2.764633000	-1.719957000
6	0.250214000	0.784197000	-2.779658000
1	0.756850000	-0.188124000	-2.753027000
1	0.901466000	1.548957000	-2.344423000
1	0.006174000	1.044152000	-3.824118000
6	1.752691000	1.900071000	0.333530000
6	-4.551379000	-2.391415000	-0.810136000
6	-3.914831000	-1.690884000	-1.835507000
1	-4.327330000	-1.655746000	-2.844603000
6	-1.884265000	-0.322310000	-2.572612000
1	-2.504324000	0.122070000	-3.366744000
1	-1.252032000	-1.087865000	-3.048303000
8	1.568882000	2.624334000	-0.650063000
6	3.099259000	1.954079000	1.057856000
1	3.161898000	1.156788000	1.810197000
1	3.166003000	2.921077000	1.580477000
1	-5.489524000	-2.915303000	-1.005311000
6	4.275462000	1.859715000	0.092856000
6	4.442171000	0.653051000	-0.703574000
1	5.243483000	2.119391000	0.552216000
1	4.173748000	2.628354000	-0.716232000
6	3.415639000	-0.262156000	-1.113541000

1	5.423413000	0.534405000	-1.185554000
6	3.809640000	-1.645010000	-1.640772000
1	2.519667000	-0.292824000	-0.475648000
1	3.102814000	0.381086000	-1.990835000
1	3.065563000	-1.928587000	-2.399528000
1	4.785051000	-1.588772000	-2.152670000
6	3.825094000	-2.726186000	-0.553348000
1	3.862113000	-3.704512000	-1.061313000
1	2.859180000	-2.691133000	-0.019342000
6	4.977434000	-2.670431000	0.456708000
1	5.938603000	-2.765110000	-0.079350000
1	4.902417000	-3.561751000	1.103065000
6	5.017096000	-1.432142000	1.351345000
1	5.738457000	-1.555473000	2.175094000
1	5.362980000	-0.531422000	0.808400000
1	4.031961000	-1.206299000	1.789269000
1	0.105118000	-1.966306000	-0.813154000

Optimized geometry of ^{trans}**II$_d$** [UB3LYP/def2SVP/GD3BJ/SMD (acetonitrile)]
Electronic energy: -2608.37998006 a.u.
Lowest frequency: 11.08 cm^{-1}

Atomic Number	x	y	z
26	-1.297691000	0.332830000	-0.632945000
8	0.411754000	-0.610774000	-0.718844000
7	-1.581910000	-0.533392000	1.105299000
6	-2.410659000	-1.579974000	1.190356000
8	-0.872142000	1.007819000	-2.262667000
7	-0.320327000	1.736526000	0.589416000
7	-2.955897000	1.577262000	-0.405215000
7	-2.683435000	-1.105762000	-1.229699000
6	-1.128139000	-0.595327000	3.432393000
1	-0.595530000	-0.196009000	4.296468000
6	-0.915660000	-0.055986000	2.167863000
6	0.089011000	1.001473000	1.815657000
1	1.037772000	0.490696000	1.593654000
1	0.268273000	1.696592000	2.650051000
6	0.866812000	2.322636000	-0.060683000
1	1.353836000	3.048806000	0.611244000
1	0.554516000	2.817729000	-0.986548000
1	1.567688000	1.519016000	-0.306569000
6	-1.310523000	2.807611000	0.909998000
1	-1.197563000	3.594539000	0.154831000
1	-1.080824000	3.258258000	1.888029000
6	-2.728332000	2.269361000	0.889799000
1	-2.901903000	1.558387000	1.705236000
1	-3.447779000	3.094852000	1.016060000
6	-3.121678000	2.595180000	-1.470463000
1	-3.936182000	3.283013000	-1.191857000
1	-3.390282000	2.121537000	-2.421632000
1	-2.197725000	3.162160000	-1.615065000
6	-4.141805000	0.686038000	-0.391106000
1	-5.065458000	1.261300000	-0.564373000
1	-4.228187000	0.223933000	0.600803000
6	-3.950846000	-0.360420000	-1.466847000
1	-4.801533000	-1.059694000	-1.494502000
1	-3.882324000	0.108189000	-2.456618000
6	-2.273644000	-1.805514000	-2.466595000
1	-1.348943000	-2.359732000	-2.271704000
1	-2.096689000	-1.065967000	-3.255433000
1	-3.062197000	-2.507351000	-2.786525000
6	0.745628000	-1.829029000	-0.473682000
6	-2.035000000	-1.654160000	3.557670000
6	-2.677220000	-2.165436000	2.428515000
1	-3.363813000	-3.010382000	2.497308000
6	-2.878648000	-2.103616000	-0.130522000
1	-3.924649000	-2.442643000	-0.096957000
1	-2.234524000	-2.964633000	-0.347579000
8	-0.004729000	-2.775283000	-0.240463000
6	2.264559000	-2.035479000	-0.452694000
1	2.487123000	-3.104101000	-0.578783000
1	2.602116000	-1.745826000	0.556802000
1	-2.230588000	-2.089934000	4.539903000
6	2.966309000	-1.197850000	-1.504912000
6	4.377593000	-0.920530000	-1.409202000
1	2.544935000	-0.158784000	-1.504980000
1	2.735014000	-1.499631000	-2.544580000
6	5.268937000	-1.330819000	-0.368617000

1	4.823160000	-0.398477000	-2.267709000
6	6.607871000	-0.621479000	-0.159865000
1	4.759107000	-1.634388000	0.557876000
1	5.459047000	-2.321657000	-0.896937000
1	7.333835000	-1.386994000	0.151001000
1	6.964474000	-0.216247000	-1.121577000
6	6.580773000	0.476241000	0.913949000
1	7.625430000	0.655918000	1.217769000
1	6.068569000	0.085025000	1.811925000
6	5.967449000	1.826010000	0.523538000
1	6.522895000	2.240191000	-0.336873000
1	6.144825000	2.523041000	1.360601000
6	4.471799000	1.820974000	0.205824000
1	4.075916000	2.847981000	0.160813000
1	4.250991000	1.373160000	-0.775806000
1	3.893137000	1.272888000	0.967474000
1	-1.641616000	1.275676000	-2.783722000

Optimized geometry ^{cis}**IRC1$_q$** [UB3LYP/def2SVP/GD3BJ/SMD (acetonitrile)]
Electronic energy: -2608.34510061 a.u.

Atomic Number	x	y	z
26	-0.798987000	-0.212178000	0.101934000
8	0.515216000	0.376265000	-0.918737000
7	-1.749067000	1.354362000	-0.486127000
6	-1.648782000	2.476167000	0.245858000
8	0.288516000	-1.538688000	0.815259000
7	-1.807070000	-1.084137000	-1.514288000
7	-2.477837000	-0.857826000	1.193994000
7	-0.422962000	0.980390000	1.785721000
6	-3.084754000	2.422190000	-2.120635000
1	-3.615660000	2.382058000	-3.072106000
6	-2.385490000	1.309519000	-1.668101000
6	-2.135476000	0.050382000	-2.429508000
1	-1.259890000	0.229580000	-3.070972000
1	-2.978338000	-0.224083000	-3.079179000
6	-0.996478000	-2.073621000	-2.261840000
1	-1.554904000	-2.391331000	-3.157240000
1	-0.798059000	-2.936424000	-1.620176000
1	-0.052145000	-1.606436000	-2.565583000
6	-3.060070000	-1.729701000	-0.996694000
1	-2.809547000	-2.763011000	-0.734697000
1	-3.810243000	-1.763234000	-1.800343000
6	-3.585625000	-0.985123000	0.209410000
1	-3.949934000	0.016159000	-0.047264000
1	-4.425476000	-1.535017000	0.661436000
6	-2.248405000	-2.170484000	1.858174000
1	-3.181870000	-2.467651000	2.360339000
1	-1.445519000	-2.087601000	2.594836000
1	-1.953414000	-2.927585000	1.127716000
6	-2.727615000	0.186406000	2.223236000
1	-3.411681000	-0.203321000	2.993250000
1	-3.223144000	1.043270000	1.751343000
6	-1.402994000	0.566951000	2.841174000
1	-1.524096000	1.387670000	3.563549000
1	-0.964390000	-0.281393000	3.378075000
6	0.953622000	0.852040000	2.314258000
1	1.669877000	1.168012000	1.548385000
1	1.138504000	-0.190310000	2.588626000
1	1.059105000	1.498313000	3.200361000
6	0.783067000	-2.730855000	0.535861000
6	-3.070571000	3.579608000	-1.335521000
6	-2.330548000	3.621911000	-0.150572000
1	-2.264720000	4.529231000	0.450648000
6	-0.656917000	2.391883000	1.359351000
1	-0.941436000	3.009857000	2.222931000
1	0.292664000	2.786794000	0.967644000
8	0.116615000	-3.679976000	0.158107000
6	2.283393000	-2.828539000	0.749428000
1	2.530037000	-3.894605000	0.845622000
1	2.555563000	-2.316467000	1.685173000
1	-3.620460000	4.464187000	-1.663340000
6	3.077931000	-2.220154000	-0.422089000
6	3.099437000	-0.730734000	-0.484012000
1	2.723325000	-2.639675000	-1.381189000
1	4.126301000	-2.575762000	-0.328239000
6	3.673928000	-0.015361000	-1.663299000

1	1.354483000	-0.113178000	-0.701440000
1	3.092646000	-0.199553000	0.474803000
6	5.223840000	-0.021321000	-1.683823000
1	3.323543000	1.029671000	-1.667599000
1	3.313461000	-0.480399000	-2.597115000
1	5.572090000	-1.050974000	-1.871515000
1	5.554432000	0.583408000	-2.546520000
6	5.879930000	0.503345000	-0.403303000
1	6.976569000	0.460299000	-0.528314000
1	5.647072000	-0.175884000	0.436711000
6	5.480868000	1.928735000	-0.015644000
1	4.390097000	1.978395000	0.146489000
1	5.693492000	2.607810000	-0.861606000
6	6.195608000	2.427115000	1.238409000
1	5.895250000	3.455914000	1.496309000
1	5.967808000	1.785838000	2.107094000
1	7.290773000	2.422771000	1.104380000

Optimized geometry ^{cis}**IRC2$_q$** [UB3LYP/def2SVP/GD3BJ/SMD (acetonitrile)]
Electronic energy: -2608.34764170 a.u.

Atomic Number	x	y	z
26	-0.866470000	-0.223512000	0.095489000
8	0.450062000	0.366979000	-0.935423000
7	-1.825590000	1.345267000	-0.503882000
6	-1.727269000	2.463944000	0.221926000
8	0.232344000	-1.546683000	0.814080000
7	-1.881081000	-1.109502000	-1.523657000
7	-2.543897000	-0.867616000	1.192739000
7	-0.493626000	0.989620000	1.779715000
6	-3.164233000	2.392927000	-2.140991000
1	-3.696496000	2.346523000	-3.091865000
6	-2.459459000	1.286485000	-1.680585000
6	-2.207474000	0.018793000	-2.436731000
1	-1.332251000	0.199301000	-3.078986000
1	-3.051982000	-0.250303000	-3.089585000
6	-1.062956000	-2.096674000	-2.258598000
1	-1.610615000	-2.431064000	-3.156564000
1	-0.857382000	-2.954963000	-1.611927000
1	-0.117675000	-1.628868000	-2.562641000
6	-3.123340000	-1.751738000	-0.996791000
1	-2.871952000	-2.783540000	-0.725214000
1	-3.884149000	-1.796907000	-1.792272000
6	-3.650272000	-1.003473000	0.210964000
1	-4.016942000	-0.004338000	-0.053060000
1	-4.491488000	-1.553571000	0.663274000
6	-2.309462000	-2.174097000	1.862528000
1	-3.238974000	-2.479143000	2.370197000
1	-1.504702000	-2.085905000	2.597399000
1	-2.009282000	-2.933885000	1.136207000
6	-2.795385000	0.180172000	2.214904000
1	-3.477853000	-0.203628000	2.990843000
1	-3.292217000	1.034396000	1.738178000
6	-1.469014000	0.573509000	2.829638000
1	-1.601512000	1.392223000	3.555138000
1	-1.030183000	-0.273584000	3.370562000
6	0.882161000	0.863426000	2.300731000
1	1.598376000	1.175741000	1.531431000
1	1.073876000	-0.177452000	2.578924000
1	1.001072000	1.512833000	3.184816000
6	0.722534000	-2.726360000	0.543933000
6	-3.153518000	3.554569000	-1.362170000
6	-2.413061000	3.606517000	-0.177998000
1	-2.351427000	4.518041000	0.417931000
6	-0.732980000	2.388905000	1.340970000
1	-1.022732000	3.020971000	2.194703000
1	0.213975000	2.786521000	0.944384000
8	0.065368000	-3.686221000	0.168559000
6	2.229381000	-2.821211000	0.757189000
1	2.488198000	-3.883199000	0.854298000
1	2.496705000	-2.303138000	1.690132000
1	-3.706986000	4.435380000	-1.694520000
6	3.007978000	-2.209294000	-0.416885000
6	3.053524000	-0.733431000	-0.491672000
1	2.686566000	-2.637716000	-1.383051000
1	4.080714000	-2.519428000	-0.326076000
6	3.594524000	-0.015478000	-1.669837000
1	1.258363000	-0.129364000	-0.719364000

1	2.991626000	-0.182830000	0.459209000
6	5.160839000	-0.014024000	-1.686893000
1	3.254326000	1.030642000	-1.665434000
1	3.254104000	-0.497182000	-2.598374000
1	5.500388000	-1.044051000	-1.872442000
1	5.460479000	0.592193000	-2.556856000
6	5.808842000	0.522186000	-0.411817000
1	6.904078000	0.483216000	-0.543571000
1	5.583065000	-0.155142000	0.431587000
6	5.402005000	1.947099000	-0.031611000
1	4.310184000	1.989934000	0.131107000
1	5.610601000	2.622061000	-0.881191000
6	6.114587000	2.455088000	1.219783000
1	5.807800000	3.483100000	1.471743000
1	5.890604000	1.816930000	2.091526000
1	7.209390000	2.456461000	1.084650000

Optimized geometry of ^{cis}**II**$_q$ [UB3LYP/def2SVP/GD3BJ/SMD (acetonitrile)]
Electronic energy: -2608.39240984 a.u.
Lowest frequency: 23.50 cm^{-1}

Atomic Number	x	y	z
26	0.667338000	-0.440213000	0.036599000
8	-0.855706000	0.519350000	0.328019000
7	1.747178000	1.276365000	0.067478000
6	1.992338000	1.915357000	-1.088277000
8	-0.319550000	-2.053297000	-0.046238000
7	1.466186000	-0.446989000	2.177453000
7	2.478408000	-1.549325000	-0.306230000
7	0.885140000	-0.012574000	-2.190793000
6	3.120679000	2.800407000	1.268439000
1	3.549061000	3.122660000	2.218436000
6	2.270577000	1.697263000	1.231637000
6	1.821280000	0.954236000	2.461195000
1	0.918052000	1.463095000	2.833197000
1	2.583913000	1.025082000	3.254561000
6	0.455073000	-0.933174000	3.127778000
1	0.846063000	-0.935557000	4.161040000
1	0.161628000	-1.953837000	2.853321000
1	-0.429482000	-0.285733000	3.079738000
6	2.646851000	-1.340030000	2.152371000
1	2.289337000	-2.364699000	2.315448000
1	3.333997000	-1.097985000	2.981316000
6	3.389055000	-1.253877000	0.831440000
1	3.816466000	-0.254581000	0.684539000
1	4.230760000	-1.966145000	0.830958000
6	2.242768000	-3.007981000	-0.391064000
1	3.205500000	-3.523648000	-0.541047000
1	1.568420000	-3.234174000	-1.221489000
1	1.769455000	-3.369125000	0.526683000
6	3.050801000	-1.059662000	-1.589431000
1	3.823899000	-1.760907000	-1.944556000
1	3.551049000	-0.101303000	-1.403646000
6	1.969363000	-0.911147000	-2.643912000
1	2.415363000	-0.539934000	-3.582871000
1	1.517888000	-1.887742000	-2.860875000
6	-0.370094000	-0.292240000	-2.906631000
1	-1.162593000	0.354863000	-2.509828000

1	-0.662455000	-1.336105000	-2.740279000
1	-0.259244000	-0.104773000	-3.989805000
6	-1.605811000	-2.232266000	-0.168956000
6	3.409843000	3.464315000	0.074804000
6	2.837444000	3.023313000	-1.119297000
1	3.040234000	3.523745000	-2.067226000
6	1.243368000	1.412251000	-2.293340000
1	1.809965000	1.621828000	-3.215903000
1	0.309011000	1.992832000	-2.351404000
8	-2.135008000	-2.602677000	-1.211579000
6	-2.430598000	-1.999963000	1.089464000
1	-2.086086000	-1.068253000	1.556355000
1	-2.199320000	-2.814583000	1.794225000
1	4.082812000	4.324453000	0.075282000
6	-3.926995000	-1.958850000	0.839663000
6	-4.468903000	-0.897993000	0.002633000
1	-4.508190000	-1.888956000	1.783360000
1	-4.324625000	-2.901391000	0.406707000
6	-3.733414000	0.024913000	-0.788289000
1	-5.563824000	-0.887647000	-0.099779000
6	-4.405870000	1.265641000	-1.366580000
1	-2.684622000	0.171848000	-0.469806000
1	-3.630352000	-0.794666000	-1.582960000
1	-4.084475000	1.368446000	-2.414025000
1	-5.500270000	1.130017000	-1.378662000
6	-4.029580000	2.555568000	-0.624015000
1	-4.387394000	3.400431000	-1.235946000
1	-2.927937000	2.642109000	-0.601094000
6	-4.579200000	2.727636000	0.797633000
1	-5.682444000	2.675706000	0.770167000
1	-4.334621000	3.753740000	1.122155000
6	-4.047393000	1.749323000	1.849064000
1	-4.285570000	2.100101000	2.866144000
1	-4.495304000	0.746556000	1.759954000
1	-2.954210000	1.629310000	1.776354000
1	-0.676417000	1.472866000	0.334745000

Optimized geometry ^{trans}**IRC1$_q$** [UB3LYP/def2SVP/GD3BJ/SMD (acetonitrile)]
Electronic energy: -2608.34894312 a.u.

Atomic Number	x	y	z
26	-0.705768000	-0.101037000	0.345817000
8	0.183128000	0.892203000	-0.990543000
7	-2.336306000	-0.070490000	-0.736301000
6	-3.297927000	0.823867000	-0.448785000
8	0.752116000	-0.051317000	1.309701000
7	-0.310107000	-1.781169000	-0.826722000
7	-1.537753000	-1.450533000	1.684837000
7	-1.781245000	1.348037000	1.415438000
6	-3.626491000	-1.045272000	-2.466420000
1	-3.720308000	-1.774244000	-3.271948000
6	-2.441958000	-0.953701000	-1.746342000
6	-1.164823000	-1.669239000	-2.046366000
1	-0.618659000	-1.059695000	-2.781944000
1	-1.328949000	-2.663474000	-2.485318000
6	1.111686000	-1.880912000	-1.227993000
1	1.249019000	-2.781753000	-1.847031000
1	1.735867000	-1.955217000	-0.330872000
1	1.385312000	-0.990978000	-1.802069000
6	-0.687057000	-2.976675000	-0.007154000
1	0.190531000	-3.252970000	0.587920000
1	-0.914158000	-3.819900000	-0.675781000
6	-1.863647000	-2.663002000	0.885941000
1	-2.775280000	-2.471057000	0.309350000
1	-2.070623000	-3.507838000	1.560053000
6	-0.603386000	-1.817493000	2.784365000
1	-1.117178000	-2.540994000	3.434816000
1	-0.323662000	-0.933716000	3.361915000
1	0.308564000	-2.263245000	2.381073000
6	-2.738507000	-0.782641000	2.253154000
1	-3.076151000	-1.321963000	3.151264000
1	-3.552926000	-0.827799000	1.519854000
6	-2.360081000	0.636256000	2.602618000
1	-3.232078000	1.198485000	2.966544000
1	-1.604140000	0.652431000	3.395833000
6	-0.940514000	2.470023000	1.897967000
1	-0.540537000	3.026913000	1.046245000
1	-0.123724000	2.067466000	2.506650000
1	-1.560642000	3.144094000	2.510627000
6	0.263003000	2.164224000	-1.315114000
6	-4.673504000	-0.177522000	-2.136350000
6	-4.507417000	0.784070000	-1.136135000
1	-5.295454000	1.497073000	-0.892301000
6	-2.874069000	1.863790000	0.534060000
1	-3.707987000	2.239791000	1.142882000
1	-2.452462000	2.688921000	-0.058001000
8	-0.650832000	2.969569000	-1.233774000
6	1.655429000	2.542416000	-1.794492000
1	1.595473000	3.546429000	-2.235951000
1	1.971434000	1.832956000	-2.575710000
1	-5.619546000	-0.239248000	-2.678090000
6	2.682079000	2.530294000	-0.645311000
6	3.044057000	1.185225000	-0.111155000
1	2.338898000	3.185965000	0.176510000
1	3.604468000	3.019073000	-1.026455000

6	3.911612000	1.026346000	1.094985000
1	1.462448000	0.418999000	0.804964000
1	2.998864000	0.343008000	-0.810042000
6	5.429102000	1.033129000	0.777430000
1	3.671880000	0.073715000	1.598839000
1	3.701506000	1.831280000	1.819660000
1	5.717674000	2.044160000	0.443237000
1	5.972359000	0.848041000	1.720983000
6	5.870914000	0.013893000	-0.277897000
1	6.965945000	0.091188000	-0.400807000
1	5.439458000	0.285918000	-1.258118000
6	5.508991000	-1.439475000	0.036918000
1	4.412556000	-1.540766000	0.115947000
1	5.911325000	-1.707681000	1.031015000
6	6.026516000	-2.422104000	-1.011494000
1	5.750438000	-3.460960000	-0.766951000
1	5.612998000	-2.193677000	-2.008804000
1	7.125959000	-2.379307000	-1.093626000

Optimized geometry ^{trans}**IRC2$_q$** [UB3LYP/def2SVP/GD3BJ/SMD (acetonitrile)]
Electronic energy: -2608.36556681 a.u.

Atomic Number	x	y	z
26	0.693522000	0.146196000	0.367708000
8	-0.270612000	-0.922595000	-0.880310000
7	2.306585000	0.009974000	-0.742927000
6	3.258397000	-0.869310000	-0.407116000
8	-0.767898000	0.188422000	1.366168000
7	0.322803000	1.789474000	-0.916566000
7	1.578088000	1.568822000	1.626281000
7	1.768052000	-1.280661000	1.519259000
6	3.555552000	0.810644000	-2.586110000
1	3.636920000	1.466885000	-3.453546000
6	2.394750000	0.803093000	-1.820864000
6	1.125025000	1.540103000	-2.136968000
1	0.535355000	0.888410000	-2.800081000
1	1.319351000	2.478540000	-2.679643000
6	-1.106875000	1.899556000	-1.256685000
1	-1.271946000	2.756742000	-1.931764000
1	-1.690101000	2.043430000	-0.339365000
1	-1.433372000	0.980497000	-1.754339000
6	0.779479000	2.999345000	-0.185595000
1	-0.069714000	3.369925000	0.400654000
1	1.053643000	3.792445000	-0.899787000
6	1.949761000	2.689945000	0.733188000
1	2.843421000	2.408075000	0.164129000
1	2.206709000	3.582783000	1.326768000
6	0.656149000	2.046008000	2.679937000
1	1.170842000	2.810810000	3.284674000
1	0.345998000	1.217918000	3.322226000
1	-0.247554000	2.473473000	2.238334000
6	2.738540000	0.886663000	2.243738000
1	3.095479000	1.454112000	3.118819000
1	3.564281000	0.853182000	1.521084000
6	2.310132000	-0.506191000	2.667217000
1	3.157298000	-1.050753000	3.114328000
1	1.524113000	-0.443912000	3.429855000
6	0.898520000	-2.364226000	2.015751000
1	0.507999000	-2.945163000	1.173530000
1	0.066085000	-1.929446000	2.581124000
1	1.474126000	-3.039317000	2.672328000
6	-0.229162000	-2.168673000	-1.229260000
6	4.597765000	-0.046323000	-2.216068000
6	4.448836000	-0.911839000	-1.130159000
1	5.234150000	-1.614884000	-0.849565000
6	2.864431000	-1.825114000	0.679078000
1	3.725013000	-2.119453000	1.299487000
1	2.480106000	-2.716088000	0.164298000
8	0.755553000	-2.894080000	-1.204303000
6	-1.607350000	-2.663336000	-1.663239000
1	-1.520289000	-3.697601000	-2.018692000
1	-1.956051000	-2.032424000	-2.495793000
1	5.525661000	-0.052271000	-2.791882000
6	-2.607171000	-2.588176000	-0.506092000
6	-3.072602000	-1.267101000	-0.081474000
1	-2.287172000	-3.178492000	0.373069000
1	-3.565421000	-3.093700000	-0.798405000
6	-3.901057000	-1.040275000	1.110325000

1	-1.420904000	-0.354604000	0.899720000
1	-2.956084000	-0.434142000	-0.787134000
6	-5.440723000	-1.041675000	0.757817000
1	-3.678015000	-0.049225000	1.538642000
1	-3.728194000	-1.823975000	1.860146000
1	-5.707659000	-2.064031000	0.451892000
1	-5.949805000	-0.832344000	1.711774000
6	-5.859017000	-0.041008000	-0.314264000
1	-6.952970000	-0.122959000	-0.437375000
1	-5.422757000	-0.334120000	-1.286050000
6	-5.495600000	1.415389000	-0.017498000
1	-4.398636000	1.514511000	0.063471000
1	-5.902700000	1.695976000	0.970611000
6	-6.005401000	2.383548000	-1.082570000
1	-5.725092000	3.423540000	-0.849775000
1	-5.588521000	2.139365000	-2.074443000
1	-7.104492000	2.343642000	-1.167031000

Optimized geometry of $^{trans}\mathbf{II}_q$ [UB3LYP/def2SVP/GD3BJ/SMD (acetonitrile)]
Electronic energy: -2608.38613412 a.u.
Lowest frequency: 24.08 cm^{-1}

Atomic Number	x	y	z
26	-1.413956000	0.074184000	-0.807574000
8	0.282799000	-0.838286000	-1.089696000
7	-1.205536000	-0.253741000	1.162932000
6	-1.943502000	-1.199705000	1.765018000
8	-1.571875000	0.210726000	-2.593793000
7	-0.229132000	1.907972000	-0.182196000
7	-3.068870000	1.444955000	-0.377304000
7	-3.024683000	-1.467192000	-0.452463000
6	-0.215470000	0.376044000	3.232674000
1	0.477575000	1.016165000	3.780215000
6	-0.345065000	0.517589000	1.854239000
6	0.482671000	1.465742000	1.030623000
1	1.382245000	0.919691000	0.709028000
1	0.818590000	2.321881000	1.638605000
6	0.717160000	2.297356000	-1.237521000
1	1.310494000	3.179496000	-0.938584000
1	0.159407000	2.527798000	-2.153650000
1	1.392907000	1.459591000	-1.441952000
6	-1.198151000	2.993507000	0.086562000
1	-1.336056000	3.552372000	-0.847745000
1	-0.794794000	3.700993000	0.830574000
6	-2.534023000	2.455093000	0.568647000
1	-2.438505000	1.987982000	1.555107000
1	-3.250233000	3.287250000	0.673257000
6	-3.592703000	2.120275000	-1.582007000
1	-4.368482000	2.850079000	-1.296829000
1	-4.047327000	1.391410000	-2.264113000
1	-2.789559000	2.648992000	-2.108590000
6	-4.138709000	0.628187000	0.248445000
1	-5.094500000	1.177036000	0.224755000
1	-3.885167000	0.473136000	1.304965000
6	-4.288058000	-0.700921000	-0.468586000
1	-5.100045000	-1.284584000	-0.001476000
1	-4.567778000	-0.539638000	-1.518210000
6	-3.007970000	-2.479779000	-1.521129000
1	-2.037820000	-2.991845000	-1.507534000
1	-3.135184000	-1.978073000	-2.488445000
1	-3.816546000	-3.219574000	-1.381290000
6	0.702426000	-1.933006000	-0.538143000
6	-0.991871000	-0.586009000	3.883945000
6	-1.864091000	-1.384978000	3.146141000
1	-2.479313000	-2.148353000	3.624594000
6	-2.764252000	-2.080143000	0.866528000
1	-3.702446000	-2.378896000	1.362512000
1	-2.160568000	-2.982652000	0.703176000
8	-0.000624000	-2.837310000	-0.099932000
6	2.224113000	-2.017830000	-0.420891000
1	2.518767000	-3.072040000	-0.326694000
1	2.474975000	-1.522439000	0.533085000
1	-0.916811000	-0.710131000	4.966397000
6	2.951926000	-1.333778000	-1.561680000
6	4.356524000	-1.019576000	-1.431456000
1	2.494564000	-0.343437000	-1.792117000
1	2.819473000	-1.837185000	-2.541873000
6	5.218931000	-1.364978000	-0.346642000

1	4.825275000	-0.544390000	-2.304597000
6	6.524461000	-0.601497000	-0.115230000
1	4.691105000	-1.662708000	0.570661000
1	5.461631000	-2.358604000	-0.853053000
1	7.264137000	-1.325647000	0.256227000
1	6.904841000	-0.219585000	-1.077234000
6	6.405298000	0.533680000	0.912002000
1	7.429014000	0.785761000	1.235111000
1	5.891691000	0.146669000	1.810916000
6	5.723690000	1.828641000	0.454425000
1	6.289645000	2.257443000	-0.391644000
1	5.815959000	2.556533000	1.278635000
6	4.247162000	1.715175000	0.069542000
1	3.775981000	2.709488000	0.018469000
1	4.105641000	1.270905000	-0.928918000
1	3.678268000	1.114800000	0.797774000
1	-2.306023000	0.775994000	-2.875031000

Optimized geometry of ${}^{cis}\mathbf{II}_{\mathbf{S}}$ [UB3LYP/def2SVP/GD3BJ/SMD (acetonitrile)]
Electronic energy: -2608.40322889 a.u.
Lowest frequency: 29.49 cm^{-1}

Atomic Number	x	y	z
26	0.911311000	0.722852000	-0.264243000
8	1.706121000	2.035250000	-1.279769000
7	2.655673000	-0.472303000	-0.452505000
6	2.716248000	-1.389847000	-1.422356000
8	-0.177752000	3.779007000	-0.540288000
7	2.036171000	1.169652000	1.635554000
7	0.139334000	-0.968155000	1.124029000
7	0.331569000	-0.802703000	-1.791118000
6	4.787171000	-1.089889000	0.379220000
1	5.592327000	-0.945832000	1.101114000
6	3.650514000	-0.284032000	0.422019000
6	3.462943000	0.887627000	1.349323000
1	3.881571000	1.768498000	0.838573000
1	4.028782000	0.747747000	2.284488000
6	1.858947000	2.594776000	1.974763000
1	2.454933000	2.865619000	2.863677000
1	0.799608000	2.788939000	2.187233000
1	2.166663000	3.210982000	1.121809000
6	1.507124000	0.306029000	2.720877000
1	0.622447000	0.805505000	3.136103000
1	2.247715000	0.222432000	3.533776000
6	1.126938000	-1.074946000	2.217478000
1	2.007734000	-1.613364000	1.847141000
1	0.718469000	-1.668085000	3.054580000
6	-1.195231000	-0.668724000	1.673403000
1	-1.476905000	-1.426898000	2.423776000
1	-1.938919000	-0.677700000	0.873094000
1	-1.207483000	0.323894000	2.136539000
6	0.091000000	-2.182231000	0.281605000
1	-0.544309000	-2.956627000	0.745600000
1	1.101356000	-2.603866000	0.208627000
6	-0.458007000	-1.848105000	-1.094414000
1	-0.513494000	-2.758673000	-1.713600000
1	-1.478340000	-1.462514000	-0.993437000
6	-0.490710000	-0.126677000	-2.814008000
1	0.086683000	0.695864000	-3.254942000
1	-1.386329000	0.288235000	-2.335104000
1	-0.796679000	-0.830971000	-3.606771000
6	-1.048282000	2.963215000	-0.236908000
6	4.861043000	-2.079137000	-0.606449000
6	3.821753000	-2.233551000	-1.527950000
1	3.864433000	-2.990461000	-2.312601000
6	1.567156000	-1.354721000	-2.395097000
1	1.380128000	-2.350083000	-2.828825000
1	1.864855000	-0.690416000	-3.221324000
8	-0.874040000	1.683174000	-0.093223000
6	-2.472908000	3.441283000	0.020971000
1	-2.566088000	4.484347000	-0.303229000
1	-2.649319000	3.397971000	1.108561000
1	5.735192000	-2.732087000	-0.657372000
6	-3.468704000	2.559092000	-0.707862000
6	-3.657656000	1.224680000	-0.215813000
1	-3.404834000	2.600367000	-1.806630000
1	-4.519626000	2.903738000	-0.475123000

6	-4.171280000	0.132369000	-1.011370000
1	1.175203000	2.858288000	-1.159765000
1	-3.506720000	1.057795000	0.859074000
6	-5.272298000	-0.723946000	-0.339078000
1	-3.253948000	-0.488748000	-1.154914000
1	-4.451578000	0.478751000	-2.016745000
1	-6.074549000	-0.041994000	-0.016689000
1	-5.699219000	-1.353346000	-1.135916000
6	-4.829115000	-1.607906000	0.827936000
1	-5.737101000	-1.964965000	1.343737000
1	-4.279026000	-1.010150000	1.576465000
6	-3.987928000	-2.817383000	0.416209000
1	-3.076372000	-2.479579000	-0.102590000
1	-4.554167000	-3.409163000	-0.324773000
6	-3.597329000	-3.707861000	1.592791000
1	-2.986623000	-4.564654000	1.264816000
1	-3.013232000	-3.149191000	2.342093000
1	-4.489507000	-4.109343000	2.102553000

Optimized geometry of ^{trans}**II$_S$** [UB3LYP/def2SVP/GD3BJ/SMD (acetonitrile)]
Electronic energy: -2608.39454406 a.u.
Lowest frequency: 22.25 cm^{-1}

Atomic Number	x	y	z
26	-1.407218000	0.038037000	-0.917673000
8	0.274253000	-0.974615000	-1.108016000
7	-1.169353000	-0.231526000	1.195013000
6	-1.877456000	-1.168582000	1.831775000
8	-1.716310000	0.251320000	-2.714268000
7	-0.280153000	1.880545000	-0.268504000
7	-3.156544000	1.442159000	-0.320791000
7	-2.981423000	-1.486589000	-0.373479000
6	-0.172069000	0.500671000	3.221955000
1	0.510544000	1.174209000	3.742096000
6	-0.321519000	0.581818000	1.840128000
6	0.470871000	1.498984000	0.947704000
1	1.367509000	0.948166000	0.625865000
1	0.812701000	2.391051000	1.496862000
6	0.649027000	2.277243000	-1.341893000
1	1.221945000	3.176807000	-1.057802000
1	0.076654000	2.482733000	-2.254803000
1	1.347027000	1.455487000	-1.539059000
6	-1.254144000	2.971270000	-0.002604000
1	-1.440198000	3.481247000	-0.956207000
1	-0.810402000	3.713699000	0.681300000
6	-2.566488000	2.461394000	0.569897000
1	-2.420637000	2.017245000	1.561441000
1	-3.257841000	3.312955000	0.698034000
6	-3.756298000	2.083072000	-1.505209000
1	-4.532049000	2.805357000	-1.196016000
1	-4.225821000	1.330390000	-2.150522000
1	-2.993073000	2.610630000	-2.088737000
6	-4.153076000	0.586595000	0.356632000
1	-5.140820000	1.078917000	0.375485000
1	-3.847484000	0.449168000	1.401729000
6	-4.269234000	-0.750818000	-0.353176000
1	-5.045559000	-1.367158000	0.131419000
1	-4.578604000	-0.600347000	-1.395868000
6	-2.995546000	-2.524371000	-1.423026000
1	-2.022538000	-3.031365000	-1.433042000
1	-3.164659000	-2.045682000	-2.395530000
1	-3.792922000	-3.264645000	-1.234497000
6	0.753685000	-2.028848000	-0.521897000
6	-0.922600000	-0.456372000	3.913897000
6	-1.783401000	-1.306896000	3.218491000
1	-2.373247000	-2.065725000	3.734958000
6	-2.676328000	-2.085727000	0.947171000
1	-3.598245000	-2.419133000	1.450004000
1	-2.039415000	-2.963748000	0.773486000
8	0.088918000	-2.959605000	-0.081274000
6	2.272373000	-2.023924000	-0.365539000
1	2.620623000	-3.054032000	-0.209493000
1	2.476627000	-1.466009000	0.565032000
1	-0.834930000	-0.537168000	4.999684000
6	2.987318000	-1.361279000	-1.527615000
6	4.383351000	-1.006287000	-1.401672000
1	2.496605000	-0.403875000	-1.813277000
1	2.890543000	-1.918656000	-2.483773000

6	5.256716000	-1.319188000	-0.315547000
1	4.839008000	-0.534395000	-2.283509000
6	6.546789000	-0.524986000	-0.102659000
1	4.738770000	-1.610813000	0.609241000
1	5.518509000	-2.315681000	-0.805564000
1	7.302257000	-1.225976000	0.281242000
1	6.916804000	-0.154391000	-1.073041000
6	6.405310000	0.627777000	0.901291000
1	7.424199000	0.914218000	1.210770000
1	5.908525000	0.246717000	1.812101000
6	5.684848000	1.892907000	0.420583000
1	6.230967000	2.317374000	-0.440549000
1	5.763503000	2.642182000	1.226821000
6	4.208844000	1.728476000	0.053029000
1	3.708078000	2.707067000	-0.017222000
1	4.070652000	1.257569000	-0.934001000
1	3.664798000	1.128428000	0.800038000
1	-2.489177000	0.661710000	-3.123110000

Optimized geometry of ^{cis}**TS(II-III)$_s$** [UB3LYP/def2SVP/GD3BJ/SMD (acetonitrile)]
Electronic energy: -2608.39806373 a.u.
Lowest frequency: -18.05 cm^{-1}

Atomic Number	x	y	z
26	-1.007589000	-0.523686000	-0.347826000
8	-0.939561000	-1.057328000	-2.110459000
7	-2.775641000	0.608970000	-0.644194000
6	-2.660540000	1.888327000	-1.018783000
8	1.452034000	-2.297131000	-1.719341000
7	-2.604136000	-1.846262000	0.490373000
7	-1.076402000	0.253644000	1.856616000
7	-0.230648000	1.561742000	-0.613564000
6	-5.136106000	0.776925000	-0.502160000
1	-6.100145000	0.310380000	-0.294065000
6	-3.959369000	0.035340000	-0.402638000
6	-3.897806000	-1.440988000	-0.108370000
1	-4.004039000	-1.966653000	-1.070086000
1	-4.741350000	-1.754031000	0.527480000
6	-2.270757000	-3.234833000	0.116877000
1	-3.040680000	-3.939625000	0.475818000
1	-1.303277000	-3.502258000	0.560824000
1	-2.187587000	-3.302540000	-0.975447000
6	-2.598250000	-1.680408000	1.966642000
1	-1.797309000	-2.317751000	2.363257000
1	-3.548174000	-2.047157000	2.390765000
6	-2.364125000	-0.238231000	2.387675000
1	-3.169381000	0.410664000	2.020509000
1	-2.384010000	-0.176747000	3.490248000
6	0.037807000	-0.241064000	2.683710000
1	-0.040759000	0.166285000	3.707795000
1	0.998559000	0.054094000	2.248962000
1	0.024082000	-1.334765000	2.725154000
6	-1.019898000	1.725291000	1.748047000
1	-0.778395000	2.183381000	2.723699000
1	-2.011649000	2.096225000	1.460882000
6	0.027492000	2.143539000	0.728257000
1	0.071246000	3.243653000	0.662154000
1	1.019443000	1.799694000	1.049035000
6	1.017178000	1.469523000	-1.393974000
1	0.811735000	0.957689000	-2.341931000
1	1.748592000	0.880016000	-0.827170000
1	1.440616000	2.469377000	-1.591548000
6	1.465611000	-2.190201000	-0.489940000
6	-5.041104000	2.122240000	-0.869529000
6	-3.793007000	2.692649000	-1.137945000
1	-3.697144000	3.739035000	-1.431368000
6	-1.259066000	2.321088000	-1.362907000
1	-1.131276000	3.405640000	-1.216696000
1	-1.113364000	2.116535000	-2.434985000
8	0.598128000	-1.546240000	0.218219000
6	2.582788000	-2.853598000	0.310900000
1	2.399898000	-3.939441000	0.297274000
1	2.552429000	-2.516927000	1.355394000
1	-5.945102000	2.730546000	-0.945658000
6	3.951040000	-2.578091000	-0.314639000
6	4.494616000	-1.255185000	-0.093369000

1	4.035502000	-2.898717000	-1.362946000
1	4.732606000	-3.158657000	0.246358000
6	5.287760000	-0.524576000	-1.023152000
1	-0.100329000	-1.558521000	-2.241103000
1	4.226170000	-0.763399000	0.853067000
6	6.226925000	0.593643000	-0.566935000
1	4.325585000	-0.077889000	-1.454420000
1	5.666138000	-1.140790000	-1.852684000
1	7.164658000	0.106296000	-0.252945000
1	6.472624000	1.194864000	-1.456117000
6	5.715690000	1.501519000	0.554172000
1	6.534364000	2.189129000	0.825826000
1	5.517595000	0.906880000	1.463797000
6	4.473394000	2.320841000	0.194842000
1	3.655985000	1.648457000	-0.111275000
1	4.698235000	2.944176000	-0.689018000
6	3.980342000	3.200711000	1.340536000
1	3.095794000	3.787463000	1.044814000
1	3.699660000	2.592591000	2.217289000
1	4.759069000	3.910780000	1.666653000

Optimized geometry of ^{cis}**III$_d$** [UB3LYP/def2SVP/GD3BJ/SMD (acetonitrile)]
Electronic energy: -2608.45156894 a.u.
Lowest frequency: 25.66 cm^{-1}

Atomic Number	x	y	z
26	-0.743331000	-0.295984000	0.205756000
8	0.788931000	-0.344114000	-1.114173000
7	-1.183805000	1.408289000	-0.607921000
6	-0.769992000	2.543488000	-0.016552000
8	-0.008205000	-1.784780000	1.006972000
7	-1.991054000	-1.034766000	-1.326218000
7	-2.411325000	-0.321162000	1.365181000
7	0.049577000	0.970072000	1.694507000
6	-2.375743000	2.613569000	-2.266682000
1	-2.994446000	2.611084000	-3.164619000
6	-1.931012000	1.411125000	-1.725288000
6	-2.111952000	0.057229000	-2.337099000
1	-1.307800000	-0.075550000	-3.077017000
1	-3.065536000	-0.024463000	-2.879042000
6	-1.535069000	-2.260659000	-2.018809000
1	-2.245077000	-2.516403000	-2.822194000
1	-1.483503000	-3.086811000	-1.303512000
1	-0.545435000	-2.083697000	-2.453602000
6	-3.311534000	-1.298973000	-0.677304000
1	-3.277928000	-2.316229000	-0.271768000
1	-4.112653000	-1.267755000	-1.431301000
6	-3.569016000	-0.300657000	0.427015000
1	-3.688360000	0.718248000	0.039266000
1	-4.492883000	-0.555146000	0.969046000
6	-2.492744000	-1.539537000	2.217700000
1	-3.453807000	-1.522933000	2.754281000
1	-1.669155000	-1.553348000	2.935549000
1	-2.409878000	-2.443151000	1.608244000
6	-2.368140000	0.890024000	2.223810000
1	-3.106032000	0.804684000	3.036825000
1	-2.647031000	1.761116000	1.618641000
6	-0.973574000	1.017407000	2.785588000
1	-0.861793000	1.949368000	3.359726000
1	-0.759549000	0.186577000	3.467287000
6	1.334838000	0.542918000	2.291568000
1	2.126410000	0.578356000	1.537923000
1	1.233596000	-0.478850000	2.670323000
1	1.605094000	1.224321000	3.114321000
6	0.302660000	-2.997996000	0.600097000
6	-2.005128000	3.804427000	-1.636098000
6	-1.179250000	3.778212000	-0.508138000
1	-0.850245000	4.695807000	-0.019389000
6	0.225450000	2.315758000	1.075575000
1	0.196217000	3.106931000	1.839460000
1	1.224071000	2.344608000	0.610564000
8	-0.506571000	-3.912004000	0.567839000
6	1.756780000	-3.172774000	0.211792000
1	1.938190000	-4.242715000	0.040687000
1	2.379000000	-2.850659000	1.063421000
1	-2.350196000	4.760519000	-2.034930000
6	2.171681000	-2.377774000	-1.036387000
6	2.151269000	-0.860094000	-0.913930000
1	1.572103000	-2.675976000	-1.911109000
1	3.209849000	-2.663059000	-1.263530000
6	3.046601000	-0.151651000	-1.926777000

1	0.864568000	0.499740000	-1.593494000
1	2.450182000	-0.574546000	0.100311000
6	4.547453000	-0.262078000	-1.627384000
1	2.764509000	0.916175000	-1.934982000
1	2.833882000	-0.543929000	-2.935465000
1	4.880255000	-1.301057000	-1.788573000
1	5.080762000	0.346556000	-2.377158000
6	4.966796000	0.182219000	-0.219887000
1	6.065408000	0.103448000	-0.144341000
1	4.569163000	-0.524490000	0.530492000
6	4.542656000	1.602644000	0.160479000
1	3.444539000	1.694910000	0.098465000
1	4.947548000	2.314387000	-0.581565000
6	4.992505000	2.002630000	1.563287000
1	4.671980000	3.026595000	1.815827000
1	4.569722000	1.324229000	2.323801000
1	6.090666000	1.962130000	1.660093000

Optimized geometry of $^{trans}\mathbf{III_d}$ [UB3LYP/def2SVP/GD3BJ/SMD (acetonitrile)]
Electronic energy: -2608.45188212 a.u.
Lowest frequency: 13.60 cm^{-1}

Atomic Number	x	y	z
26	1.125473000	0.472861000	0.170587000
8	-2.157672000	-0.351757000	0.031169000
7	2.527328000	-0.780139000	-0.417570000
6	2.943841000	-1.727436000	0.433918000
8	-0.197555000	1.581208000	0.667389000
7	1.474768000	1.260198000	-1.715415000
7	2.481370000	1.841640000	0.851351000
7	1.421132000	-0.525336000	1.964274000
6	4.022006000	-1.524198000	-2.100633000
1	4.406350000	-1.440765000	-3.117949000
6	2.988177000	-0.694454000	-1.672511000
6	2.193960000	0.239455000	-2.534627000
1	1.437798000	-0.372370000	-3.049927000
1	2.806670000	0.722606000	-3.309810000
6	0.228313000	1.662526000	-2.403372000
1	0.464147000	2.131756000	-3.372617000
1	-0.313971000	2.369960000	-1.766604000
1	-0.396574000	0.778318000	-2.578875000
6	2.337155000	2.460203000	-1.491368000
1	1.673454000	3.303931000	-1.269555000
1	2.887410000	2.705381000	-2.412750000
6	3.287048000	2.221276000	-0.337083000
1	3.996830000	1.415647000	-0.560849000
1	3.876131000	3.129134000	-0.131154000
6	1.885590000	3.063868000	1.445624000
1	2.700272000	3.725630000	1.779708000
1	1.245654000	2.804219000	2.291612000
1	1.262999000	3.580086000	0.711571000
6	3.265953000	1.127746000	1.890994000
1	3.853453000	1.842233000	2.489380000
1	3.977266000	0.451633000	1.401846000
6	2.299910000	0.371655000	2.778809000
1	2.839678000	-0.219010000	3.534695000
1	1.646963000	1.071437000	3.313449000
6	0.162732000	-0.783449000	2.699073000
1	-0.475588000	-1.458524000	2.116738000
1	-0.358828000	0.167121000	2.855207000
1	0.381798000	-1.254675000	3.671185000
6	-1.311672000	-1.163584000	-0.569560000
6	4.532364000	-2.464586000	-1.200911000
6	3.976479000	-2.590527000	0.076031000
1	4.326142000	-3.348379000	0.778145000
6	2.108136000	-1.818953000	1.674839000
1	2.684164000	-2.158920000	2.548106000
1	1.332104000	-2.575197000	1.480386000
8	-0.099329000	-0.962863000	-0.560985000
6	-2.028906000	-2.299623000	-1.235555000
1	-1.463928000	-3.235308000	-1.134529000
1	-2.087207000	-2.051743000	-2.310197000
1	5.351812000	-3.119420000	-1.504687000
6	-3.400448000	-2.272991000	-0.556618000
6	-3.559701000	-0.806435000	-0.148630000
1	-3.394038000	-2.909750000	0.341406000
1	-4.215887000	-2.601184000	-1.212691000
6	-4.311915000	-0.527024000	1.134918000

1	-1.051044000	1.134893000	0.553812000
1	-3.945070000	-0.201233000	-0.982302000
6	-5.780190000	-0.969446000	1.078133000
1	-4.244834000	0.550742000	1.352616000
1	-3.799709000	-1.055263000	1.956176000
1	-5.823717000	-2.068445000	0.993047000
1	-6.241685000	-0.720984000	2.048942000
6	-6.615541000	-0.348413000	-0.048164000
1	-7.643700000	-0.744109000	0.028300000
1	-6.239712000	-0.688999000	-1.029936000
6	-6.671825000	1.180722000	-0.036052000
1	-5.657034000	1.593500000	-0.171484000
1	-7.007931000	1.522085000	0.960182000
6	-7.592299000	1.751447000	-1.112691000
1	-7.612920000	2.853265000	-1.087239000
1	-7.262325000	1.447680000	-2.120947000
1	-8.628436000	1.394721000	-0.984112000

Optimized geometry of $^{cis}\mathbf{III_q}$ [UB3LYP/def2SVP/GD3BJ/SMD (acetonitrile)]
Electronic energy: -2608.45275589 a.u.
Lowest frequency: 21.97 cm^{-1}

Atomic Number	x	y	z
26	-0.642175000	-0.267904000	0.188850000
8	0.970767000	-0.241025000	-1.221434000
7	-1.434743000	1.245255000	-0.740707000
6	-1.187958000	2.486914000	-0.269956000
8	0.377733000	-1.517480000	1.068410000
7	-1.971033000	-1.363578000	-1.187408000
7	-2.427818000	-0.436726000	1.526475000
7	-0.095040000	1.288266000	1.583128000
6	-2.939489000	2.088960000	-2.373130000
1	-3.620570000	1.901163000	-3.203751000
6	-2.268222000	1.025917000	-1.777305000
6	-2.319920000	-0.388799000	-2.257783000
1	-1.577695000	-0.486622000	-3.066254000
1	-3.300131000	-0.629860000	-2.696610000
6	-1.390188000	-2.567390000	-1.820663000
1	-2.105383000	-2.989740000	-2.546080000
1	-1.171463000	-3.316688000	-1.054354000
1	-0.466020000	-2.297357000	-2.345135000
6	-3.176940000	-1.752098000	-0.398873000
1	-2.941196000	-2.697799000	0.103105000
1	-4.017207000	-1.942149000	-1.085222000
6	-3.564951000	-0.699941000	0.624481000
1	-3.856610000	0.240880000	0.138714000
1	-4.440892000	-1.054451000	1.193851000
6	-2.259624000	-1.536850000	2.499263000
1	-3.171648000	-1.630110000	3.113308000
1	-1.402758000	-1.338723000	3.150957000
1	-2.055731000	-2.479467000	1.981480000
6	-2.496169000	0.871119000	2.206884000
1	-3.171757000	0.824077000	3.078114000
1	-2.919056000	1.608294000	1.512341000
6	-1.111216000	1.282328000	2.679473000
1	-1.150404000	2.280951000	3.141275000
1	-0.751965000	0.582653000	3.442686000
6	1.254618000	1.111102000	2.160763000
1	2.002796000	1.153399000	1.361718000
1	1.309948000	0.139168000	2.662043000
1	1.461843000	1.917519000	2.883179000
6	0.671830000	-2.774570000	0.764488000
6	-2.719565000	3.378438000	-1.884639000
6	-1.829306000	3.586611000	-0.827822000
1	-1.633016000	4.584726000	-0.435078000
6	-0.142891000	2.551157000	0.798339000
1	-0.287795000	3.418952000	1.459260000
1	0.830069000	2.676975000	0.297424000
8	-0.119561000	-3.690967000	0.896905000
6	2.093796000	-2.961590000	0.275000000
1	2.269529000	-4.039014000	0.151837000
1	2.766965000	-2.595465000	1.068677000
1	-3.243093000	4.226579000	-2.330555000
6	2.429319000	-2.226571000	-1.035079000
6	2.338005000	-0.701129000	-0.990537000
1	1.801332000	-2.597085000	-1.861818000
1	3.464468000	-2.496096000	-1.292610000
6	3.249034000	0.011901000	-1.984781000

1	0.825378000	-0.084476000	-2.171084000
1	2.565021000	-0.355168000	0.023798000
6	4.744636000	-0.075583000	-1.649451000
1	2.939370000	1.069040000	-2.012432000
1	3.071731000	-0.399296000	-2.994732000
1	5.102380000	-1.105594000	-1.814694000
1	5.287878000	0.553575000	-2.375113000
6	5.114759000	0.354776000	-0.224172000
1	6.209469000	0.272998000	-0.106189000
1	4.686362000	-0.358495000	0.502713000
6	4.676910000	1.771869000	0.152505000
1	3.590406000	1.880537000	-0.005625000
1	5.153529000	2.494368000	-0.534465000
6	5.005923000	2.133629000	1.598593000
1	4.681511000	3.157806000	1.845173000
1	4.505313000	1.447206000	2.302737000
1	6.090212000	2.070352000	1.792273000

Optimized geometry of $^{trans}\mathbf{III_q}$ [UB3LYP/def2SVP/GD3BJ/SMD (acetonitrile)]
Electronic energy: -2608.45261641 a.u.
Lowest frequency: 7.58 cm^{-1}

Atomic Number	x	y	z
26	1.072245000	0.164009000	0.424418000
8	-2.217761000	-0.439358000	-0.037751000
7	2.467359000	-0.296672000	-0.938946000
6	3.194772000	-1.416912000	-0.781536000
8	-0.162588000	0.611799000	1.635574000
7	1.063153000	2.010055000	-0.811265000
7	2.555250000	1.263570000	1.595273000
7	2.014479000	-1.605641000	1.384326000
6	3.692945000	0.292986000	-2.889257000
1	3.856162000	0.984858000	-3.716550000
6	2.669714000	0.536014000	-1.976145000
6	1.672440000	1.651693000	-2.110865000
1	0.870506000	1.288021000	-2.772848000
1	2.121651000	2.532354000	-2.597186000
6	-0.305315000	2.529093000	-0.993638000
1	-0.296104000	3.468193000	-1.573128000
1	-0.753101000	2.711100000	-0.009219000
1	-0.912434000	1.788585000	-1.531421000
6	1.893838000	2.980270000	-0.050972000
1	1.234934000	3.477886000	0.671147000
1	2.281011000	3.755370000	-0.732147000
6	3.052229000	2.314139000	0.678166000
1	3.757481000	1.859184000	-0.028216000
1	3.609947000	3.080401000	1.242217000
6	1.996396000	1.870074000	2.818917000
1	2.791504000	2.419897000	3.350082000
1	1.585600000	1.095662000	3.473504000
1	1.179350000	2.552150000	2.567187000
6	3.585498000	0.257659000	1.940681000
1	4.239249000	0.640235000	2.742330000
1	4.220343000	0.089337000	1.061979000
6	2.932465000	-1.036115000	2.404420000
1	3.710531000	-1.772820000	2.663329000
1	2.340124000	-0.852340000	3.309544000
6	1.003956000	-2.472626000	2.018258000
1	0.316759000	-2.856746000	1.252754000
1	0.432919000	-1.884143000	2.746692000
1	1.482729000	-3.325701000	2.529193000
6	-1.410485000	-1.154362000	-0.789555000
6	4.488373000	-0.841863000	-2.718772000
6	4.233007000	-1.716194000	-1.661121000
1	4.824372000	-2.620639000	-1.512620000
6	2.743071000	-2.331709000	0.321171000
1	3.587926000	-2.904823000	0.735248000
1	2.049260000	-3.058020000	-0.130036000
8	-0.188216000	-1.005723000	-0.747964000
6	-2.173194000	-2.098135000	-1.667977000
1	-1.652637000	-3.060658000	-1.756346000
1	-2.207141000	-1.637734000	-2.671351000
1	5.303835000	-1.048339000	-3.415087000
6	-3.548748000	-2.141304000	-0.997733000
6	-3.641629000	-0.781367000	-0.301593000
1	-3.582426000	-2.946693000	-0.248001000
1	-4.371897000	-2.288217000	-1.707448000
6	-4.389380000	-0.728313000	1.013142000

1	-1.045630000	0.356160000	1.318929000
1	-3.988641000	-0.001872000	-0.995373000
6	-5.874160000	-1.090831000	0.872386000
1	-4.279983000	0.282207000	1.437756000
1	-3.905470000	-1.429004000	1.713732000
1	-5.961233000	-2.150740000	0.579349000
1	-6.332431000	-1.013321000	1.872904000
6	-6.675016000	-0.233016000	-0.114815000
1	-7.717802000	-0.596375000	-0.118505000
1	-6.303137000	-0.392096000	-1.143224000
6	-6.674551000	1.266083000	0.191630000
1	-5.642345000	1.655384000	0.151214000
1	-7.017528000	1.422603000	1.230721000
6	-7.551382000	2.069577000	-0.766325000
1	-7.534043000	3.145592000	-0.527835000
1	-7.210994000	1.954118000	-1.809643000
1	-8.601838000	1.734793000	-0.723869000

Optimized geometry of $^{trans}\mathbf{III}_{q,IRC}$ [UB3LYP/def2SVP/GD3BJ/SMD (acetonitrile)]
Electronic energy: -2608.44307461 a.u.
Lowest frequency: 16.26 cm^{-1}

Atomic Number	x	y	z
26	0.737179000	-0.576254000	-0.490449000
8	-1.087066000	1.588881000	-0.575212000
7	1.649588000	0.571871000	0.803207000
6	2.699623000	1.303498000	0.396486000
8	-0.436517000	-1.172290000	-1.684825000
7	-0.060041000	-1.369376000	1.280131000
7	2.135112000	-2.244004000	-0.340218000
7	2.141646000	0.242763000	-1.782369000
6	1.929365000	1.257049000	3.057354000
1	1.603590000	1.224403000	4.097314000
6	1.243375000	0.537915000	2.088725000
6	0.003506000	-0.275331000	2.291899000
1	-0.867841000	0.380928000	2.141902000
1	-0.062905000	-0.685016000	3.310450000
6	-1.458349000	-1.834642000	1.134860000
1	-1.810452000	-2.250481000	2.092298000
1	-1.502851000	-2.605195000	0.358157000
1	-2.098767000	-0.997315000	0.847955000
6	0.801604000	-2.538006000	1.685962000
1	0.295333000	-3.442158000	1.330679000
1	0.839510000	-2.592935000	2.783568000
6	2.208480000	-2.478902000	1.113087000
1	2.793212000	-1.668581000	1.567176000
1	2.726922000	-3.426691000	1.341263000
6	1.633691000	-3.427075000	-1.063556000
1	2.326884000	-4.275243000	-0.927992000
1	1.541672000	-3.203256000	-2.132308000
1	0.641998000	-3.711939000	-0.695484000
6	3.383213000	-1.739704000	-0.947090000
1	4.045837000	-2.569015000	-1.246043000
1	3.926747000	-1.144448000	-0.200168000
6	3.029081000	-0.901152000	-2.160529000
1	3.933981000	-0.510210000	-2.650812000
1	2.487212000	-1.506028000	-2.898001000
6	1.510066000	0.812412000	-2.997489000
1	0.810903000	1.604178000	-2.704025000
1	0.976000000	0.019601000	-3.532884000
1	2.286577000	1.234703000	-3.655130000
6	-0.571993000	2.814538000	-0.298475000
6	3.042723000	2.008760000	2.661564000
6	3.435108000	2.039841000	1.322856000
1	4.292537000	2.628030000	0.993805000
6	2.930342000	1.311560000	-1.078912000
1	3.998224000	1.208876000	-1.319757000
1	2.597274000	2.288588000	-1.450041000
8	0.572505000	3.088240000	-0.555885000
6	-1.632458000	3.670335000	0.346211000
1	-1.518692000	4.720594000	0.048649000
1	-1.475704000	3.603963000	1.437212000
1	3.606023000	2.575561000	3.405840000
6	-2.930776000	2.995710000	-0.084937000
6	-2.519143000	1.527849000	-0.230598000
1	-3.260744000	3.387799000	-1.059819000

1	-3.750176000	3.117322000	0.634529000
6	-3.249933000	0.736378000	-1.299436000
1	-0.888740000	-0.456854000	-2.163523000
1	-2.572298000	1.023590000	0.745512000
6	-4.756372000	0.606801000	-1.035688000
1	-2.796560000	-0.263502000	-1.369844000
1	-3.081135000	1.240426000	-2.265646000
1	-5.188038000	1.611926000	-0.896955000
1	-5.229839000	0.198761000	-1.944819000
6	-5.128868000	-0.282568000	0.157828000
1	-6.196440000	-0.131170000	0.395352000
1	-4.576685000	0.041517000	1.058007000
6	-4.885275000	-1.775817000	-0.075992000
1	-3.852141000	-1.936148000	-0.425201000
1	-5.537622000	-2.118962000	-0.899100000
6	-5.132466000	-2.626063000	1.167570000
1	-4.960229000	-3.696574000	0.969235000
1	-4.463125000	-2.328134000	1.992320000
1	-6.168891000	-2.516045000	1.529903000

Optimized geometry of ^{cis}**III**$_{s}$ [UB3LYP/def2SVP/GD3BJ/SMD (acetonitrile)]
Electronic energy: -2608.46477655 a.u.
Lowest frequency: 20.93 cm^{-1}

Atomic Number	x	y	z
26	0.631119000	0.391221000	0.247970000
8	-0.931462000	0.217001000	-1.140323000
7	1.395360000	-1.291914000	-0.762800000
6	1.170757000	-2.507541000	-0.239004000
8	-0.282610000	1.837607000	0.944660000
7	2.048987000	1.309222000	-1.227762000
7	2.428007000	0.422242000	1.513658000
7	0.083281000	-1.280541000	1.621160000
6	2.945819000	-2.175824000	-2.329292000
1	3.644794000	-2.012754000	-3.150568000
6	2.247119000	-1.102335000	-1.782411000
6	2.312566000	0.309598000	-2.292923000
1	1.529740000	0.419946000	-3.059793000
1	3.277412000	0.511317000	-2.783148000
6	1.477522000	2.534970000	-1.823836000
1	2.177675000	2.963902000	-2.560459000
1	1.292721000	3.274712000	-1.036549000
1	0.533641000	2.291028000	-2.327168000
6	3.278607000	1.642524000	-0.454782000
1	3.113239000	2.621838000	0.010774000
1	4.134527000	1.744058000	-1.141141000
6	3.586172000	0.602069000	0.604322000
1	3.819978000	-0.367648000	0.147903000
1	4.474031000	0.912396000	1.179544000
6	2.330370000	1.545579000	2.475091000
1	3.255056000	1.601009000	3.072794000
1	1.476018000	1.395106000	3.144284000
1	2.172038000	2.490686000	1.944863000
6	2.475051000	-0.880148000	2.223223000
1	3.170292000	-0.822890000	3.077170000
1	2.873488000	-1.635313000	1.534442000
6	1.093798000	-1.262907000	2.719152000
1	1.127046000	-2.248883000	3.209012000
1	0.746737000	-0.537777000	3.465387000
6	-1.270356000	-1.076217000	2.181086000
1	-2.008575000	-1.098619000	1.371463000
1	-1.313565000	-0.104163000	2.687158000
1	-1.513714000	-1.874898000	2.901439000
6	-0.786156000	3.033638000	0.664607000
6	2.732910000	-3.448768000	-1.793599000
6	1.835050000	-3.625109000	-0.737011000
1	1.656375000	-4.608024000	-0.299126000
6	0.128509000	-2.544839000	0.843845000
1	0.279196000	-3.406350000	1.512118000
1	-0.850257000	-2.676467000	0.356381000
8	-0.148261000	4.052359000	0.842628000
6	-2.203263000	3.008645000	0.133816000
1	-2.499638000	4.049040000	-0.057256000
1	-2.848400000	2.620404000	0.941244000
1	3.275308000	-4.306942000	-2.196140000
6	-2.429402000	2.166378000	-1.132069000
6	-2.324518000	0.651338000	-0.975073000
1	-1.758196000	2.487292000	-1.944474000

1	-3.451535000	2.387433000	-1.473169000
6	-3.176769000	-0.133747000	-1.967108000
1	-0.889993000	-0.535583000	-1.756344000
1	-2.606883000	0.370116000	0.047321000
6	-4.685457000	-0.070050000	-1.690509000
1	-2.848860000	-1.187163000	-1.928209000
1	-2.968298000	0.228516000	-2.988404000
1	-5.058129000	0.944742000	-1.908492000
1	-5.184020000	-0.736999000	-2.414189000
6	-5.105450000	-0.458263000	-0.266784000
1	-6.206012000	-0.401051000	-0.200644000
1	-4.728442000	0.291399000	0.451788000
6	-4.651121000	-1.849161000	0.181049000
1	-3.554876000	-1.931385000	0.088844000
1	-5.068679000	-2.606930000	-0.506299000
6	-5.049759000	-2.173308000	1.618546000
1	-4.712755000	-3.179702000	1.916181000
1	-4.606100000	-1.451186000	2.325243000
1	-6.144260000	-2.133895000	1.751500000

Optimized geometry of ^{trans}**III**$_s$ [UB3LYP/def2SVP/GD3BJ/SMD (acetonitrile)]
Electronic energy: -2608.45973212 a.u.
Lowest frequency: 12.71 cm^{-1}

Atomic Number	x	y	z
26	-1.091130000	-0.006998000	-0.624919000
8	2.198333000	-0.606234000	-0.046920000
7	-2.257434000	-0.022100000	1.146291000
6	-3.106692000	-1.041380000	1.333215000
8	0.148550000	-0.010206000	-1.956116000
7	-0.760046000	2.044753000	0.233795000
7	-2.745976000	1.012377000	-1.650138000
7	-2.405150000	-1.777254000	-0.934254000
6	-3.121377000	1.137652000	3.028827000
1	-3.103187000	2.011400000	3.681722000
6	-2.230829000	1.040005000	1.960866000
6	-1.137397000	2.031011000	1.666893000
1	-0.252672000	1.717165000	2.243281000
1	-1.409749000	3.041272000	2.011054000
6	0.650857000	2.453168000	0.077954000
1	0.799712000	3.484901000	0.439912000
1	0.926985000	2.394107000	-0.981344000
1	1.297500000	1.779075000	0.652996000
6	-1.639627000	2.938769000	-0.569345000
1	-1.090166000	3.199506000	-1.482542000
1	-1.822164000	3.876217000	-0.018893000
6	-2.964692000	2.287907000	-0.925012000
1	-3.552738000	2.076716000	-0.023577000
1	-3.558227000	2.980894000	-1.544736000
6	-2.372205000	1.264057000	-3.059124000
1	-3.206375000	1.761203000	-3.581717000
1	-2.137665000	0.320603000	-3.564052000
1	-1.481079000	1.898533000	-3.106097000
6	-3.916179000	0.104752000	-1.567618000
1	-4.686851000	0.409501000	-2.295302000
1	-4.358669000	0.201325000	-0.568550000
6	-3.497495000	-1.328886000	-1.842200000
1	-4.366673000	-1.999785000	-1.745719000
1	-3.126341000	-1.422073000	-2.870577000
6	-1.634234000	-2.872668000	-1.557834000
1	-0.802669000	-3.156221000	-0.899785000
1	-1.228562000	-2.529293000	-2.517181000
1	-2.275761000	-3.754144000	-1.727705000
6	1.404253000	-1.307434000	0.739728000
6	-4.030615000	0.095708000	3.230091000
6	-4.026538000	-1.013684000	2.379612000
1	-4.724945000	-1.840017000	2.518680000
6	-2.922492000	-2.200126000	0.390821000
1	-3.855508000	-2.773984000	0.279354000
1	-2.176501000	-2.871049000	0.843876000
8	0.181844000	-1.199153000	0.682774000
6	2.195331000	-2.174351000	1.674454000
1	1.713389000	-3.152313000	1.803866000
1	2.195135000	-1.664709000	2.654211000
1	-4.747856000	0.149344000	4.052117000
6	3.581710000	-2.192329000	1.026801000
6	3.624248000	-0.862595000	0.270426000
1	3.660623000	-3.028462000	0.314929000
1	4.399980000	-2.271751000	1.752949000
6	4.411351000	-0.824738000	-1.021888000

1	1.089964000	-0.126186000	-1.752615000
1	3.913344000	-0.037982000	0.938823000
6	5.911681000	-1.076540000	-0.818564000
1	4.249112000	0.154146000	-1.500731000
1	3.998960000	-1.589439000	-1.701051000
1	6.060409000	-2.112873000	-0.470886000
1	6.397749000	-1.014541000	-1.807009000
6	6.619342000	-0.120451000	0.149887000
1	7.685110000	-0.405736000	0.197122000
1	6.229928000	-0.262683000	1.174220000
6	6.519878000	1.360634000	-0.221619000
1	5.461941000	1.675847000	-0.222947000
1	6.878990000	1.498463000	-1.257924000
6	7.310122000	2.265322000	0.721426000
1	7.221467000	3.326279000	0.435864000
1	6.950600000	2.168862000	1.760257000
1	8.382857000	2.007339000	0.719057000

10. References

1. Serrano-Plana, J.; Oloo, W. N.; Acosta-Rueda, L.; Meier, K. K.; Verdejo, B.; García-España, E.; Basallote, M. G.; Münck, E.; Que, L.; Company, A.; Costas, M., Trapping a Highly Reactive Nonheme Iron Intermediate That Oxygenates Strong C—H Bonds with Stereoretention. *J. Am. Chem. Soc.* **2015,** *137*, 15833-15842.
2. Harman, D. G.; Ramachandran, A.; Gracanin, M.; Blanksby, S. J., The Loss of Carbon Dioxide from Activated Perbenzoate Anions in the Gas Phase: Unimolecular Rearrangement via Epoxidation of the Benzene Ring. *J. Org. Chem.* **2006,** *71*, 7996-8005.
3. Fan, R.; Serrano-Plana, J.; Oloo, W. N.; Draksharapu, A.; Delgado-Pinar, E.; Company, A.; Martin-Diaconescu, V.; Borrell, M.; Lloret-Fillol, J.; García-España, E.; Guo, Y.; Bominaar, E. L.; Que, L., Jr.; Costas, M.; Münck, E., Spectroscopic and DFT Characterization of a Highly Reactive Nonheme FeV–Oxo Intermediate. *J. Am. Chem. Soc.* **2018,** *140*, 3916-3928.
4. Haynes, S. W.; Sydor, P. K.; Corre, C.; Song, L.; Challis, G. L., Stereochemical Elucidation of Streptorubin B. *J. Am. Chem. Soc.* **2011,** *133*, 1793-1798.
5. Cianfanelli, M.; Olivo, G.; Milan, M.; Klein Gebbink, R. J. M.; Ribas, X.; Bietti, M.; Costas, M., Enantioselective C–H Lactonization of Unactivated Methylenes Directed by Carboxylic Acids. *J. Am. Chem. Soc.* **2020,** *142*, 1584-1593.
6. Cussó, O.; Garcia-Bosch, I.; Font, D.; Ribas, X.; Lloret-Fillol, J.; Costas, M., Highly Stereoselective Epoxidation with H2O2 Catalyzed by Electron-Rich Aminopyridine Manganese Catalysts. *Org. Lett.* **2013,** *15*, 6158-6161.
7. Frisch, M. J.; Trucks, G. W.; Schlegel, H. B.; Scuseria, G. E.; Robb, M. A.; Cheeseman, J. R.; Scalmani, G.; Barone, V.; Petersson, G. A.; Nakatsuji, H.; Li, X.; Caricato, M.; Marenich, A. V.; Bloino, J.; Janesko, B. G.; Gomperts, R.; Mennucci, B.; Hratchian, H. P.; Ortiz, J. V.; Izmaylov, A. F.; Sonnenberg, J. L.; Williams; Ding, F.; Lipparini, F.; Egidi, F.; Goings, J.; Peng, B.; Petrone, A.; Henderson, T.; Ranasinghe, D.; Zakrzewski, V. G.; Gao, J.; Rega, N.; Zheng, G.; Liang, W.; Hada, M.; Ehara, M.; Toyota, K.; Fukuda, R.; Hasegawa, J.; Ishida, M.; Nakajima, T.; Honda, Y.; Kitao, O.; Nakai, H.; Vreven, T.; Throssell, K.; Montgomery Jr., J. A.; Peralta, J. E.; Ogliaro, F.; Bearpark, M. J.; Heyd, J. J.; Brothers, E. N.; Kudin, K. N.; Staroverov, V. N.; Keith, T. A.; Kobayashi, R.; Normand, J.; Raghavachari, K.; Rendell, A. P.; Burant, J. C.; Iyengar, S. S.; Tomasi, J.; Cossi, M.; Millam, J. M.; Klene, M.; Adamo, C.; Cammi, R.; Ochterski, J. W.; Martin, R. L.; Morokuma, K.; Farkas, O.; Foresman, J. B.; Fox, D. J. *Gaussian 16 Rev. C.01*, Wallingford, CT, 2016.
8. Becke, A. D., Density-functional thermochemistry. III. The role of exact exchange. *J. Chem. Phys.* **1993,** *98*, 5648-5652.
9. Lee, C.; Yang, W.; Parr, R. G., Development of the Colle-Salvetti correlation-energy formula into a functional of the electron density. *Phys. Rev. B* **1988,** *37*, 785-789.
10. Grimme, S.; Ehrlich, S.; Goerigk, L., Effect of the damping function in dispersion corrected density functional theory. *J. Comput. Chem.* **2011,** *32*, 1456-1465.

11. Grimme, S.; Antony, J.; Ehrlich, S.; Krieg, H., A consistent and accurate ab initio parametrization of density functional dispersion correction (DFT-D) for the 94 elements H-Pu. *J. Chem. Phys.* **2010,** *132*.
12. Becke, A. D.; Johnson, E. R., Exchange-hole dipole moment and the dispersion interaction revisited. *J. Chem. Phys.* **2007,** *127*.
13. Schäfer, A.; Horn, H.; Ahlrichs, R., Fully optimized contracted Gaussian basis sets for atoms Li to Kr. *J. Chem. Phys.* **1992,** *97*, 2571-2577.
14. Grimme, S., Supramolecular Binding Thermodynamics by Dispersion-Corrected Density Functional Theory. *Chem. Eur. J.* **2012,** *18*, 9955-9964.
15. Li, Y.-P.; Gomes, J.; Mallikarjun Sharada, S.; Bell, A. T.; Head-Gordon, M., Improved Force-Field Parameters for QM/MM Simulations of the Energies of Adsorption for Molecules in Zeolites and a Free Rotor Correction to the Rigid Rotor Harmonic Oscillator Model for Adsorption Enthalpies. *J. Phys. Chem. C* **2015,** *119*, 1840-1850.
16. Weigend, F.; Ahlrichs, R., Balanced basis sets of split valence, triple zeta valence and quadruple zeta valence quality for H to Rn: Design and assessment of accuracy. *Phys. Chem. Chem. Phys.* **2005,** *7*, 3297-3305.
17. Mondal, B.; Neese, F.; Bill, E.; Ye, S., Electronic Structure Contributions of Non-Heme Oxo-Iron(V) Complexes to the Reactivity. *J. Am. Chem. Soc.* **2018,** *140*, 9531-9544.
18. G. Luchini, J. Alegre-Requena, I. Funes-Ardoiz, R. Paton, F1000Research 2020, 9.
